# Optimal fractional order automatic generation control including an excitation system for hybrid interconnected grids

**DOI:** 10.1038/s41598-025-10539-4

**Published:** 2025-07-22

**Authors:** Awadh Ba Wazir, Sultan Alghamdi, Abdullah Ali Alhussainy, Abdulraheem H. Alobaidi, N. Gowtham

**Affiliations:** 1 Department of Electrical and Computer Engineering, King Abdulaziz University, 21589 Jeddah, Saudi Arabia; 2 Center of Research Excellence in Renewable Energy and Power Systems, King Abdulaziz University, 21589 Jeddah, Saudi Arabia; 3 Department of Electrical Engineering, College of Engineering, University of Prince Mugrin, 42241 Madinah, Saudi Arabia; 4 Department of Electrical and Electronics Engineering, Manipal Institute of Technology Bengaluru, Manipal Academy of Higher Education, Manipal, India

**Keywords:** AGC-AVR loops, Fractional order controller, Hybrid optimization algorithm, Hybrid AC/DC power system, Disturbance rejection, Energy science and technology, Engineering

## Abstract

The growing complexity of hybrid interconnected power systems (IPSs) demands the implementation of advanced control strategies to optimize performance and improve system reliability. This study introduces a new approach by utilizing a novel advanced controller design to stabilize a combination of both frequency and voltage circuits (i.e., automatic generation control (AGC) and automatic voltage regulator (AVR) for a hybrid AC/DC IPS. A novel hybrid optimization technique was employed to estimate the parameters of the proposed cascaded fractional order proportional integral-fractional order proportional integral derivative double derivative (CFOPI-FOPIDD^2^) controller via the assistance of a proposed objective function. The hybrid technique consists of two different types of algorithms: the secretary bird optimization algorithm and pattern search. The objective function is created utilizing the integral of time square error (ITSE), overshoot, undershoot, and settling time, with suitable weight factors. Initially, the suggested design of AGC-AVR for a hybrid dual-area IPS, including renewable energy sources and electric vehicles, was investigated. The outcomes indicated that the CFOPI-FOPIDD^2^ controller surpasses the current AGC-AVR controllers. For example, The CFOPI-FOPIDD^2^ controller enhances AGC-AVR performance by 60.5% over the DO-FOPI-PIDD^2^ controller and 81% over the adaptive fuzzy PID (AFPID) controller. Moreover, a comparison analysis was carried out between the CFOPI-FOPIDD^2^, PID, and FOPID controllers under various conditions. As expected, CFOPI-FOPIDD^2^ showed its superiority over those controllers. The proposed approach was examined in the AGC-AVR system, where AVR is a double-input single-output (DISO) and in a hybrid six-area IPS with nonlinearity. The outcomes demonstrated that the suggested approach can effectively handle these types of IPS.

## Introduction

### Background

Automatic Generation Control (AGC) is a critical automation in electrical networks designed. It helps maintain power system stability by regulating the system’s frequency and balancing load and generation in real-time. So, it is essential for ensuring reliable and secure power grid operations. On the other hand, Automatic Voltage Regulator (AVR) is utilized in power generation systems to automatically keep a steady output voltage level from generators, regardless of load variations. AVR is essential for ensuring stable electrical supply and protecting electrical equipment from voltage fluctuation. Due to the weak interaction between the both AGC and AVR loops, the two parameters—frequency and voltage—are controlled independently^1^. However, when AGC is combined with an excitation system, it enhances the overall stability and voltage control in the power system by integrating voltage regulation with frequency and power balance control. So, this integrated management is crucial for the reliable function of modern electrical grids^2^.

The main crucial element in the AGC-AVR loops is the controller. Without the controller or with poor controller the AGC-AVR system fails to reset the voltage and frequency fluctuations to zero. So, the controller plays a crucial role in improving the stability, response time, and overall performance of the system. In this regard, various controllers have been proposed by researchers to optimize the combined AGC-AVR. These different types of controllers that are used for enhancing the AGC-AVR performance are include traditional controllers like the proportional-integral-derivative (PID) controller, flexible controllers like fuzzy logic (FL) controller, hybrid controllers like the FL-PID controller, and advanced controllers like the fractional-order PID (FOPID) controller. On the other hand, the controller in the AGC-AVR loops is required to be properly adjusted for achieving the desired balance between fast response and stability for both frequency and voltage regulation. Poor tuning can result in oscillations, instability, or slower response times. Therefore, the researchers applied optimization algorithms to fine-tune various controllers for the AGC-AVR system^3^.

Optimization methods like particle swarm optimization (PSO), genetic algorithms (GA), etc., are methods utilized for selecting the most efficient solution for addressing an issue within a set of possible solutions, usually by minimizing or maximizing an objective function. An objective function serves as the criterion for optimization, whether it’s minimizing error, maximizing efficiency, or finding the best combination of parameters in a system. The optimization process works for determining the decision variables’ values that achieve the best result according to the objective function. So, to obtain a robust AGC-AVR loops design, the efficacy of the selected controller, optimization method, and objective function must be considered.

### Literature review

Recent electrical networks incorporate a wide range of elements, that involve high voltage direct current (HVDC), renewable energy sources (RESs), energy storage systems (ESSs), microgrids (MGs), electric vehicles (EVs), smart grids (SGs), distributed generations (DGs), and conventional elements like thermal units. Therefore, for the dependable function of recent electrical networks, powerful frequency and voltage control is desired, which is what AGC-AVR provides. In this regard, recent years have seen substantial research on the appropriate design of AGC and AVR control loops. The core aspect of these designs is to substitute the classical integral (I) controller in the secondary loop of the AGC and the classical PID controller of the AVR with more robust and efficient controllers. These controllers could be divide to four types which are: classical structures of PID controllers, improved structures of PID controllers, advanced controllers, and intelligent controllers. These controllers are applied in three domains—AGC, AVR, and combined AGC-AVR systems—through the use of optimization algorithms. Optimization algorithms serve as a foundation for numerous engineering studies due to their effectiveness in identifying optimal solutions to complex problems. In recent years, various optimization techniques have been proposed, and hybrid methods have also been incorporated into control strategies. For example, references^4,5^ demonstrate the application of such hybrid optimization techniques.

Extensive research has been conducted in the area of AGC, leading to the development of numerous frequency regulation methods. Examples include, but are not limited to, such in^6^ PSO, GA and simulated annealing (SA) algorithms were used for setting the AGC system’s classic I controller in a dual-region interconnected power system (IPS). Also, PSO was used in^7^ for tuning fuzzy-I controller for optimal AGC in dual-region IPS. A comparative analysis for GA, SA and pattern search (PS) algorithms based Fuzzy-I controller For AGC of dual-region IPS was presented in^8^. In^9^, a hybrid optimization algorithm combining PSO-oriented BFOA (HBFO) was proposed to optimally tune a PID controller for AGC in a hybrid dual-region IPS^10^. proposed a Grasshopper optimization algorithm (GOA)-based tuning of a PDF plus (1 + PI) controller for AGC in a three-area IPS. Based on competitive algorithm (ICA) a cascade PDF(1 + PI) optimized controller was proposed in^11^ for AGC of a multi-MG. In^12^, a FO fuzzy-based PID (FOPID) controller, tuned using the Sunflower optimization (SFO), was proposed for modern IPS. An improvised Moth swarm algorithm (iMSA) was proposed in^13^ for tuning an advanced hybrid FO type-2 fuzzy PID (FO-T2F-PID) controller for AGC of an isolated MG. In^14^, an improved equilibrium optimization-based fuzzy tilted double integral derivative with filter (F-TIDF-2) controller has been proposed for AGC of an isolated MG. A hybrid moth flame optimization (MFO) and PS (h-MFO-PS) algorithm was used to optimally tune an adaptive fuzzy PID (A-FLC-PID) controller for two-area six-unit IPS in^15^. In^16^, Whale optimization algorithm (WOA) and SA (h-WOA-SA) were used to tune an interval type-2 fuzzy FO PID-IT2F-FO-PID controller for AGC in a hybrid dual-region IPS.

On the other hand, a wide range of techniques has been utilized to enhance the effectiveness of AVR loop. In^17^, PSO was employed to tune a PID controller for AVR loop. A novel hybrid method was proposed in^18^ for tuning the proportional-integral-double derivative (PIDD^2^) controller, combining two distinct optimization algorithms SA and Gorilla troops optimizer (GTO). In^19^, A cutting-edge hybrid technique, known as the PSO–African Vultures Optimization Algorithm (PSO–AVOA), was employed to tune PID, PIDD^2^, and FOPID controllers for the AVR loop. Equilibrium optimizer (EO) based PI controller with anti-windup protection was used in^20^ for optimal AVR loop. In^21^, a novel hybrid approach, the SA–cooperation search algorithm (SA–CSA), was applied to the AVR system of a synchronous machine.

To here, the cited literature has primarily addressed frequency and voltage control independently. Studies that consider the simultaneous regulation of both (combined AGC-AVR) are relatively limited and can be classified based on the type of controller proposed, as follows:

### Optimized classical PID controller based AGC-AVR

In this strategy researchers used optimization algorithms to tune the classical structures of PID controllers. These optimized PID performed well than those that tuned by trial and error or by Ziegler-Nichols (ZN) method. Such in^22,23^ a differential evolution- artificial electric field (DE-AEFA) algorithm used for setting the AGC-AVR system’s classic PID controller in a multi-region interconnected power system (IPS). Also, for the same purpose a multi-objective nonlinear threshold accepting (MONLTA) method used in^24^. The firefly algorithm (FA) used in^25^ for deregulated two-area IPS. In^26^, the gradient-based optimizer (GBO) algorithm used for multi-area IPS. Also, the PID controller optimized using the sine cosine algorithm (SCA) for multi-source-muti-area IPS in^27^.

### Optimized improved PID controller based AGC-AVR

As part of this approach, researchers tuned several structures derived from PID controllers using optimization methods for optimal design of AGC-AVR for IPS. Such as, a grey wolf optimizer (GWO) based the PIDD^2^ controller^28^, hybrid flower pollinated algorithm-pathfinder algorithm (ℎFPAPFA) based PID acceleration (PIDA) controller^29^, doctor and patient optimization technique (DPO) tuned PIDA regulator^30^, Archimedes optimization algorithm (AOA), learner performance-based behavior optimization (LPBO), and modified particle swarm optimization (MPSO) tuned PI-PD controller^31^, a dandelion optimizer (DO) based PI-PD controller^32^, and one-to-one-based optimizer (OOBO) based PI-PD controller^33^.

### Advanced controllers based AGC-AVR

Advanced control techniques were implemented in the AGC-AVR system to achieve better results than the PID regulator structures. Such in^34^, the tilt-integral-derivative with filter (TIDF) controller utilized in AGC-AVR loops of three-region IPS and the controller’s parameters were tuned through the Harris hawks optimization (HHO) algorithm. Also, via HHO, the integral of 2 degrees of freedom minus tilt-derivative with filter (2DOFI-TDF) regulator proposed for enhancing the AGC-AVR system for three-area IPS in^35^. The FO tilt derivative with a filter cascaded to FO proportional derivative with a filter (CFOTDNFOPDN) regulator tuned via artificial flora algorithm (AFA) was proposed in^36^ to improve the AGC-AVR system of a dual-area IPS. Also, via AFA, a new PD with filter cascade FOPID with filter (CPDN-FOPIDN) regulator was employed in AGC-AVR loops of a three-area IPS^37^. In^38^, the DO algorithm was employed for optimizing the suggested FOPI–PIDD^2^ regulator to stabilize the frequency as well as voltage in two-area IPS. The 2DOF-TID with fractional derivative and filter (2DOF-TID^μ^) controller adapted via coot method (COA) was proposed in^39^ for a two interconnected MG (IMG) operated by sustainable and RESs.

### Intelligent controllers based AGC-AVR

Because advanced regulators are complicated and uncommon in the industrial sector. As a result, experts believed that PID controllers need to be improved due to their widespread use and the limitations of complex control methods^40^. Combining FL with PID improves the dynamic performance of AGC-AVR since FL is considered an intelligent controller capable of dealing with uncertainty and nonlinearity. Regarding to this, a combination artificial electric field method (HAEFA) was used for tuning the FL-PID regulator of AGC-AVR model on dual-region IPS^41^. In^2^, the FL-PIDD^2^ controller, whose settings are adjusted with GBO, was proposed on dual-region hybrid various source IPS. In addition, a hybrid configuration of FL and advanced controllers was used in the AGC-AVR domain. Such in^42^, a novel cascaded FL-PD and TID (CFPD-TID) controller tuned by AFA was proposed for a three-area hybrid IPS. In^43^, AEFA was used to tune FL-2DOF-TIDF controller for AGC-AVR of IMG.

In addition to the capabilities of the controller, several other factors contribute to enhancing the performance of the AGC-AVR loops. Such as, by incorporation of flexible AC transmission systems (FACTS) like interline power flow controller (IPFC) and unified power flow controller (UPFC), and ESSs like redox flow batteries (RFBs), superconducting magnetic energy storage (SEMS), capacitive energy storage (CES), ultra-capacitors (UCs), and battery energy storage system (BESS), and flywheel energy storage system (FESS). Further, the high integration of RESs such as photovoltaics (PV) and wind energy generators (WEG) as well as the limitations of thermal and mechanical movements including the generation rate constraint (GRC), governor dead band (GDB), boiler dynamics (BD), and communication time delay (CTD) were considered in the design of AGC-AVR. Also, the objective function significantly impacts the AGC-AVR system efficiency, as the controller’s actions depend on its gains, and an effectively designed objective function yields appropriate gains. The most common objective functions are: the integral square error (ISE), the integral time absolute error (ITAE), the integral time square error (ITSE), and the integral absolute error (IAE). The total of the deviations in the system’s frequency, tie line power, and terminal voltage is the error here. The limitation of these objective functions is that they cannot precisely define desired AGC-AVR responses, like the desired settling-time, overshoot, etc. But they do guarantee the system stability. A concise summary of the literature regarding AGC-AVR is shown in Table 1.

**Table 1:** A concise summary of the literature regarding combined AGC-AVR loops.

Refs.	Type	Optimizer	Controller	Covered area	Generation in all areas	Improver	Nonlinear	RESs	ESSs
^2^	IPS	GBO	FL-PIDD^2^	2	6	–	GRC and GDB	PV and WEG	–
^22^	IPS	DE-AEFA	PID	2	6	HVDC	GRC	–	–
^23^	IPS	DE-AEFA	PID	2	6	HVDC and IPFC	GRC	PV and WEG	REB
^24^	IPS	NLTA	PID	2	2	–	–	–	2
^25^	IPS	FA	PID	2	4	–	GRC, GDB, and CTD	–	–
^26^	IPS	GBO	PID	4	20	–	–	PV	–
^27^	IPS	SCA	PID	2	6	IPFC	GRC and CTD	PV and WEG	REB
^28^	IPS	GWO	PIDD2	2	6	UPFC	GRC, GDB	–	SMES
^29^	IPS	ℎFPAPFA	PIDA	1	1	–	–	–	–
^30^	IMG	DPO	PIDA	2	10	–	–	PV	FESS and BESS
^31^	IPS	AOA, LPBO, and MPSO	PI-PD	2.3	2.3	–	–	–	–
^32^	IPS	DO	PI-PD	2,3	6	–	GRC, GDB and BD	–	–
^33^	IPS	OOBO	PI-PD	4	20	–	GRC, GDB and BD	PV and WEG	–
^34^	IPS	HHO	TIDF	3	6	–	GRC, GDB and BD	–	–
^35^	IPS	HHO	(2DOFI-TDF	3	6	–	GRC, GDB	–	–
^36^	IPS	AFA	CFOTDNFOPDN	3	4	–	GRC, GDB and CTD	PV	–
^37^	IPS	AFA	CPDN-FOPIDN	3	6	HVDC	GRC, GDB	–	SMES, REB and CES
^38^	IPS	DO	FOPI–PIDD^2^	2	8	EV	GRC, GDB and CTD	PV and WEG	BESS
^39^	IMG	COA	2DOF-TID^μ^	2	12	UPFC	GRC	PV and WEG	SMES and BESS
^41^	IPS	HAEFA	FL-PID	2	6	–	–	–	SMES, REB, FESS and UC
^42^	IPS	AFA	CFPD-TID	3	6	HVDC	GRC, GDB	–	REB
^43^	IMG	AEFA	FL-2DOF-TIDF	3	6	–	–	WEG	–

### Research gap and motivation

Taking into account the aforementioned, the AGC-AVR management mechanism is of considerable importance and was discussed in numerous works. Despite several approaches for AGC-AVR were suggested, it is noted that the concern of communication delays was not addressed in the majority of papers. In extreme cases, communication delays can even cause instability. Also, it is noted that all papers concerning controller structure for the AGC-AVR system regard AVR loop as a single input single output (SISO), which the generator voltage is the output and the generator reference voltage is the input. However, the potential changes in the excitation voltage should be considered in the regulator structure of the AGC-AVR loops. On the other hand, no research has been conducted on the integration of AGC-AVR control loops in a wide-region multi-source IPS, such as a six-area multi-source IPS. The present investigation addresses this gap by examining the AGC-AVR system where AVR as SISO and as a double input single output (DISO) with consider the issue of communication delays. Also, the current investigation examines a six-area AC/DC IPS including seven generation units per area.

### Challenges and contributions

The incorporation of RESs poses many obstacles owing to their intrinsic variability and intermittency. Also, the involvement of various nonlinearities raises the system’s complexity. Moreover, the AGC-AVR loops must have the suitable speed to adjust the grid frequency, power, as well as voltage to match nominal values when the changes occur. These challenges require an efficient control mechanism to preserve the system’s stability and reliability. Therefore, the goal of this paper is to present an effective design of AGC including an excitation system for hybrid AC/DC IPS with nonlinear structures. In this regard, a novel controller configuration utilizing cascaded FOPI-FOPIDD^2^ is proposed. Also, a novel hybrid method utilizing the secretary bird optimization algorithm (SBOA) and the pattern search (PS) is utilized for tuning the controller’s variables with an aid of a novel objective function. In view of the aforementioned, the main paper’s contributions are:

- Novel design of AGC-AVR using a proposed cascaded FOPI-FOPIDD^2^ (CFOPI-FOPIDD^2^) controller.
  - For a hybrid dual-are multiple-sources IPS, containing RESs and EVs.
  - For a hybrid six-are multiple -sources IPS, containing RESs, ESSs and DG units.
- A new objective function and a new hybrid optimization method using SBOA and PS (hSBOA-PS) are utilized for optimizing the CFOPI-FOPIDD^2^ controller’ parameters.
- Unlike literature, the AGC-AVR system is examined by considering the AVR both as SISO and as a DISO.
- Testing and validation.
  - The CFOPI-FOPIDD^2^ controller supremacy against PID, and FOPID controllers.
  - The effect of time-varying CTD on the AGC-AVR loop’s efficiency.
  - The effectiveness of the CFOPI-FOPIDD^2^ controller in AGC-AVR_DISO_ loops.
  - The efficacy of the CFOPI-FOPIDD^2^ regulator on the large-area IPS with BD, GRC and GDB nonlinearity.

This paper’s remaining sections are arranged as follows: Sect. 2 describes the AGC-AVR loops. Section 3 explains the controller structure and deviations management. Section 4 describes the proposed objective function while the proposed optimization algorithm is presented in Sect. 5. The simulation outcomes of the test system-1 are covered in Sect. 6. On the other hand, Sect. 7 elaborates the results for the test system-2. Section 8 summarizes the paper and suggests a perspective for additional investigation.

## Automatic Generation Control Including an Excitation System (AGC-AVR)

**Fig. 1:**
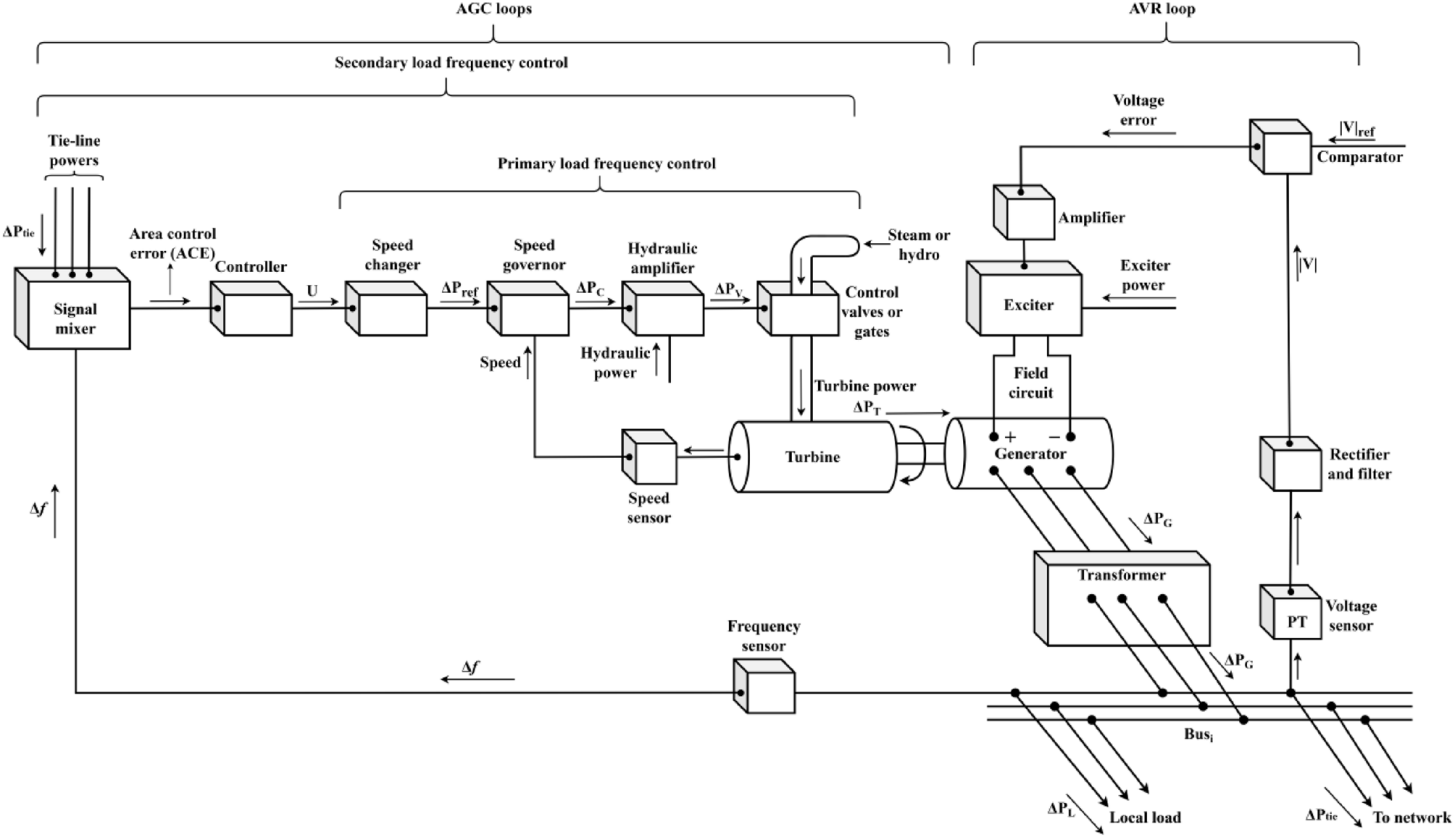
AGC-AVR loops of a synchronous generator.

In IPS, AGC and AVR work together to coordinate frequency and voltage management, ensuring that both active and reactive power are properly handled. Figure 1 shows the coupling between AGC-AVR loops. AGC manages the frequency by adjusting generator outputs through the load frequency control (LFC) loops. Also, it facilitates the maintenance of power balance across several regions by regulating the transmission along tie-lines between them. As shown in Fig. 1, by modifying generator speeds, primary frequency control responds to frequency variations instantly. On the basis of variations in frequency and tie line power, secondary management modifies the generating levels in real-time. On the other hand, the AVR loop’s goal is to regulate the generator’s excitation system for controlling the voltage level at the generator’s output. The generator’s output voltage decreases as the generator’s reactive power load increases. This is measured using a potential-transformer (PT) and the voltage is then rectified and assessed to the dc set point signal. After that, the exciter field is controlled and the exciter terminal voltage is raised via amplified error signal. As a result, the generated electromagnetic field (emf) rises as the generator field current increases.

### AGC mathematical model

**Fig. 2:**
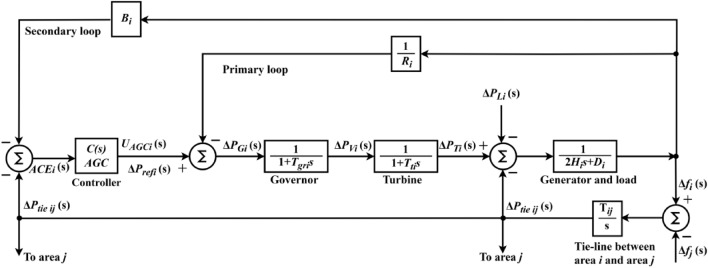
Generalized AGC loops of IPS Model.

The linearized AGC loop model is demonstrated by Fig. 2, and the mathematical modeling of AGC system is available here in details^1^. To facilitate frequency domain investigations, the components are represented using transfer functions. The AGC loop’s elements include the controller, speed governing, turbine, as well as generator and load.

In Fig. 2, ${\text{ACE}}_{i}$ is an area control error. ${B}_{i}$ is the frequency bias parameter. ${R}_{i}$ is the governor speed regulation parameter (Hz/p.u.). ${\Delta P}_{refi}$ is the output from the controller. ${T}_{gri}$ is the speed governor time constant in seconds. ${T}_{ti}$ is the turbine time constant in seconds. ${H}_{i}$ denotes the inertia constant, and ${D}_{i}$ is the frequency dependency of load (p.u./Hz). $\Delta {P}_{Gi}$ is the output command of the speed governor (p.u.)*.* $\Delta {P}_{Vi}$ is the change in the governor valve position (p.u.). $\Delta {P}_{Ti}$ is the change in turbine output power (p.u.). $\Delta {P}_{Li}$ is the load demand change (p.u.). ${T}_{ij}$ is the tie-line coefficient. $\Delta {P}_{tie ij}$ is the incremental change in tie line power (p.u.). $\Delta {f}_{i}$ is the system frequency deviation in Hz. ${U}_{AGCi}$ is the controller signal. $i$ and $j$ are subscripts referred to the corresponding area.

The speed governor mechanism functions as a comparator, with $\Delta {P}_{Gi}$ being the difference between ${\Delta P}_{refi}$ and the power $\frac{1}{{R}_{i}}\Delta {f}_{i}$. The speed governor’s hydraulic amplifier translates $\Delta {P}_{Gi}$ to $\Delta {P}_{Vi}$. Equation (1) shows the governor’s transfer function^1^.

1 $${G}_{Gi}\left(s\right)=\frac{\Delta {P}_{Vi}\left(s\right)}{\Delta {P}_{Gi}\left(s\right)}=\frac{1}{1+s{T}_{gri}}$$

The turbine, often known as the primary mover, generates mechanical power. So, the turbine model relates $\Delta {P}_{Ti}$ to $\Delta {P}_{Vi}$ and its transfer function is represented by Eq. (2)^1^.

2 $${G}_{Ti}\left(s\right)=\frac{\Delta {P}_{Ti}\left(s\right)}{\Delta {P}_{Vi}\left(s\right)}=\frac{1}{1+s{T}_{ti}}$$

The generator equation is obtained using the swing equation, as Eq. (3) shows^1^.

3 $$\Delta {f}_{i}\left(s\right)=\frac{1}{2{H}_{i}s}\left(\Delta {P}_{Ti}\left(s\right)-\Delta {P}_{ei}\left(s\right)\right)$$

Since the load of the grid is composed from multiple electric devices, Eq. (4) provides the speed load characteristic of this composite load. Equation (5) illustrates the transfer function for the generator and load model^1^.

4 $$\Delta {P}_{ei}=\Delta {P}_{Li}+{D}_{i}\Delta {f}_{i}$$

5 $${G}_{GLi}\left(s\right)=\frac{1}{{D}_{i}+s2{H}_{i}}=\frac{{K}_{psi}}{1+s{T}_{psi}}$$

$\Delta {P}_{ei}$ is the change in electrical power. ${K}_{psi}$ refers to the generator and load gain. ${T}_{psi}$ represents the time constant of the generator and load model. ${K}_{psi}$ $=\frac{1}{{D}_{i}}$ and ${T}_{psi}$ $=\frac{2{H}_{i}}{{f}_{i}^\circ {D}_{i}}$. $D$ $=\frac{\Delta {P}_{L}}{\Delta f^\circ }$. ${P}_{L}$ represents the nominal load and $f^\circ$ represents the steady state frequency.

### AVR mathematical model

**Fig. 3:**
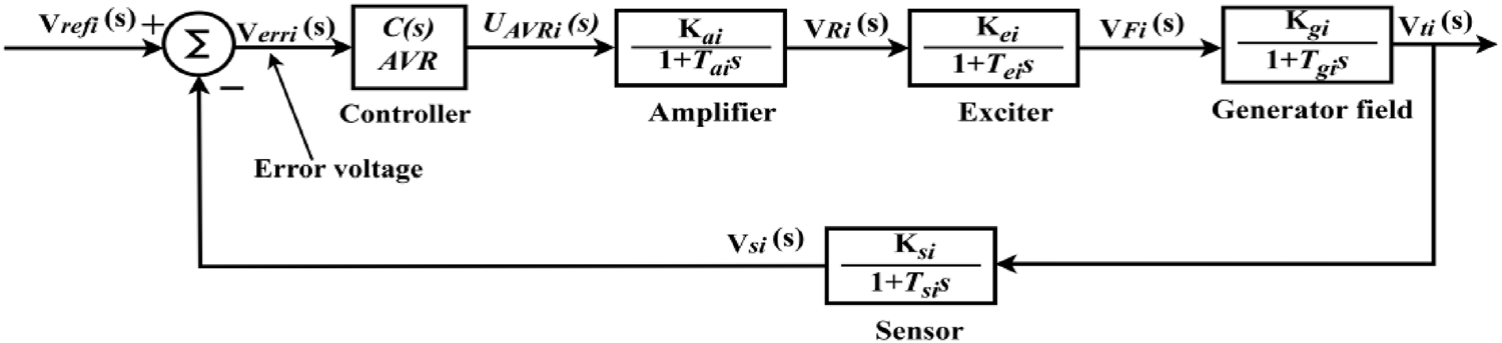
Generalized AVR loop of a synchronous generator.

Figure 3 represents the linearized AVR loop model, and its mathematical modeling is explained here^1^. The AVR loop elements include the controller, amplifier, exciter, generator field, and sensor. In Fig. 3, ${V}_{ti}$ represents the generator terminal voltage, ${V}_{refi}$ represents the reference voltage, ${V}_{erri}$ is the error voltage ($|{V}_{ti}-{V}_{refi}|$), ${V}_{Ri}$ is the amplifier voltage, ${V}_{Fi}$ is the field voltage, ${V}_{si}$ is the sensor voltage, and ${U}_{AVRi}$ is the controller signal.

The amplifier model can be represented by the transfer function illustrated in Eq. (6)^1^.

6 $$\frac{{V}_{Ri}\left(s\right)}{{V}_{erri}\left(s\right)}=\frac{{K}_{ai}}{1+s{T}_{ai}}$$

where ${K}_{ai}$ and ${T}_{ai}$ represent the gain and time constants of the amplifier. Equation (7) below indicates the exciter model’s transfer function^1^.

7 $$\frac{{V}_{Fi}\left(s\right)}{{V}_{Ri}\left(s\right)}=\frac{{K}_{ei}}{1+s{T}_{ei}}$$

${K}_{ei}$ and ${T}_{ei}$ represent the exciter’s gain and time constants. Equation (8) below shows the transfer function of the generator field model^1^.

8 $$\frac{{V}_{ti}\left(s\right)}{{V}_{Fi}\left(s\right)}=\frac{{K}_{gi}}{1+s{T}_{gi}}$$

${K}_{gi}$ and ${T}_{gi}$ represent the generator field’s gain and time constants. The sensor model represented via the transfer function illustrated in Eq. (9)^1^.

9 $$\frac{{V}_{si}\left(s\right)}{{V}_{ti}\left(s\right)}=\frac{{K}_{si}}{1+s{T}_{si}}$$

${K}_{si}$ and ${T}_{si}$ are the gain and time constants of the sensor.

### AGC-AVR mathematical model

Changes in real power have the greatest impact on system frequency, but when the modest effect of voltage on real power is taken into account, the following linearized equation (Eq. 10) obtains^1^.

10 $$\Delta {P}_{ei}(s){=P}_{si}\Delta {\delta }_{i}(s)+{K}_{1i}{E}_{i}(s)$$

${P}_{s}$ represents the synchronizing power constant, $\Delta \delta$ represents the change in the power angle, and ${K}_{1}$ represents $\Delta {P}_{e}$ for a small change in the stator emf ($E$). Also, including the modest effect of $\delta$ on the generator ${V}_{t}$, Eq. (11) is obtained^1^.

11 $$\Delta {V}_{ti}{\left(s\right)={K}_{3}{E}_{i}\left(s\right)-K}_{2}\Delta {\delta }_{i}(s)$$

where ${K}_{2}$ is the change in the terminal voltage ($\Delta {V}_{t}$) for a small $\Delta \delta$ at constant $E$, and ${K}_{3}$ is $\Delta {V}_{t}$ for a small change in $E$ at constant $\delta$. In addition, the influence of $\delta$ in the generator field transfer function can be expressed as Eq. (12)^1^.

12 $$E(s)=\frac{{K}_{gi}}{1+s{T}_{gi} }\left({{V}_{Fi}(s)-K}_{4}\Delta {\delta }_{i}(s)\right)$$

**Fig. 4:**
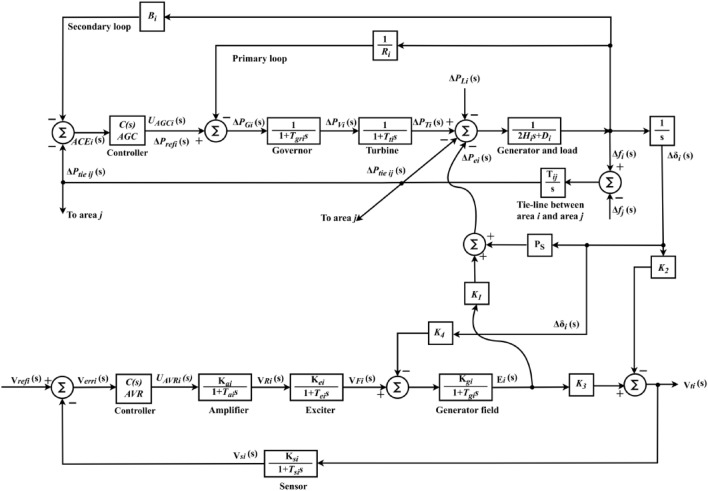
Generalized AGC-AVR loops of IPS Model.

${K}_{4}$ is coupling coefficient. Figure 4 depicts a linearized model of the AGC-AVR systems, that includes the preceding equations in the AGC and AVR systems^1^.

## Deviations management and controller structure

The controller is responsible for automatically restoring frequency and voltage fluctuations to zero in AGC-AVR Loops. Thus, the controller is provided in the second loop of AGC to remove the frequency and tie line deviations. Also, the regulator is added to the forward path in the AVR loop to reduce the steady-state error (SSE) to zero and remove the oscillations.

**Fig. 5:**
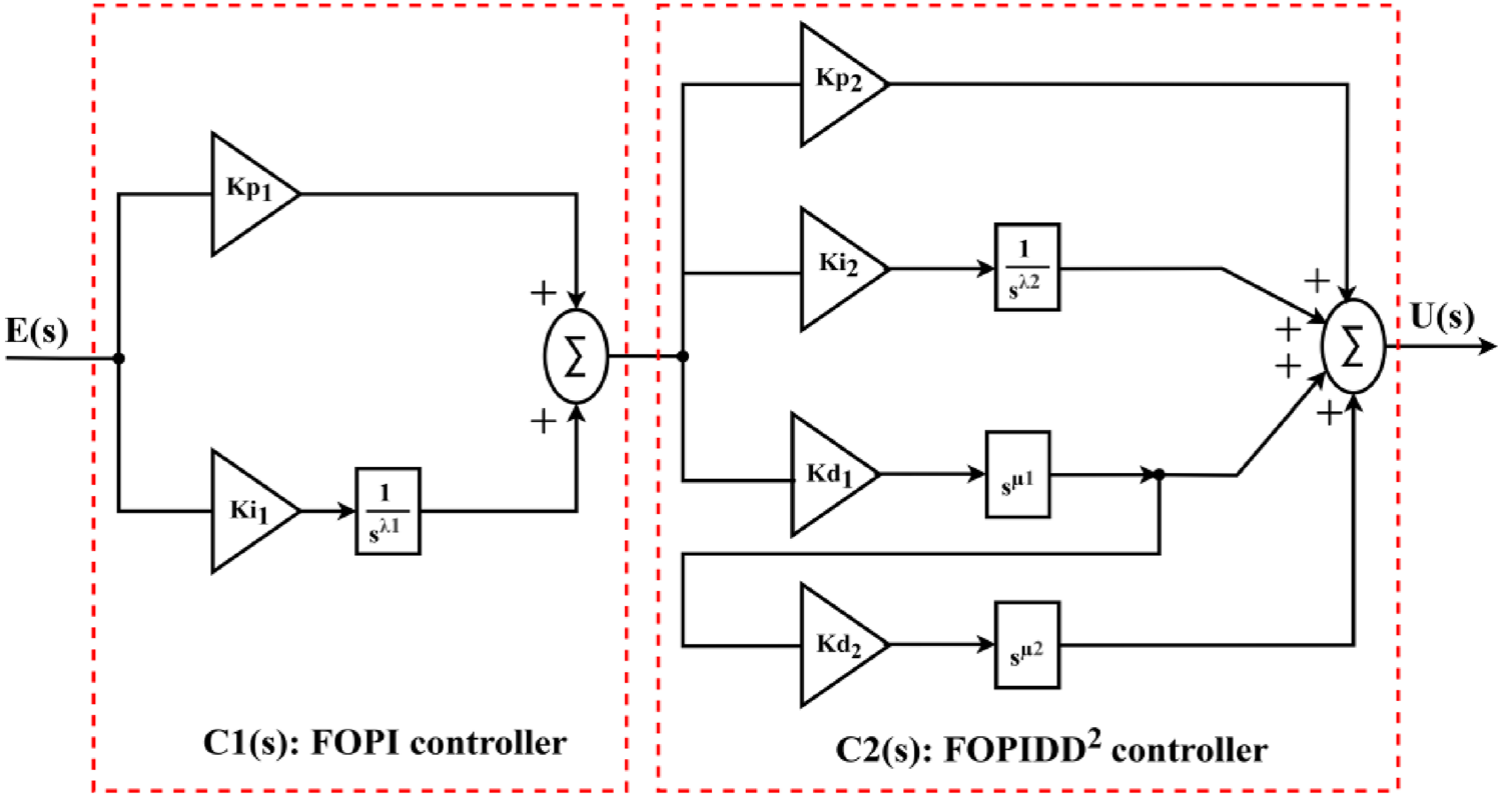
CFOPI-FOPIDD^2^ controller structure.

The proposed controller CFOPI-FOPIDD^2^ is used to design an optimal AGC-AVR system. So, it is equipped in the AGC’s second loop and before the amplifier in the AVR loop. Figure 5 showed the CFOPI-FOPIDD^2^ regulator configure. It is composed of two cascaded controllers $C1(s)$ and $C2(s)$ that are referred to FOPI and FOPIDD^2^ respectively. $E(s)$ is the input error signal and $U\left(s\right)$ represents the output control command. In AGC, $E(s)$ is $\text{ACE}(\text{s})$ while $U(s)$ is ${\Delta P}_{ref}(s)$ or ${U}_{AGC}(s)$. However, in AVR, $E(s)$ represents ${V}_{err}(s)$ while $U(s)$ is ${U}_{AVR}(s)$. ${K}_{\text{p}1}$, ${K}_{\text{i}1}$, ${K}_{p2}$, ${K}_{i2}$, ${K}_{\text{d}1}$, and ${K}_{d2}$ are the coefficients of the CFOPI-FOPIDD^2^. ${\uplambda }_{1}$ and ${\uplambda }_{2}$ are the amounts of integration while ${\mu }_{1}$ and ${\mu }_{2}$ are the amounts of amount of derivation.

**Fig. 6:**
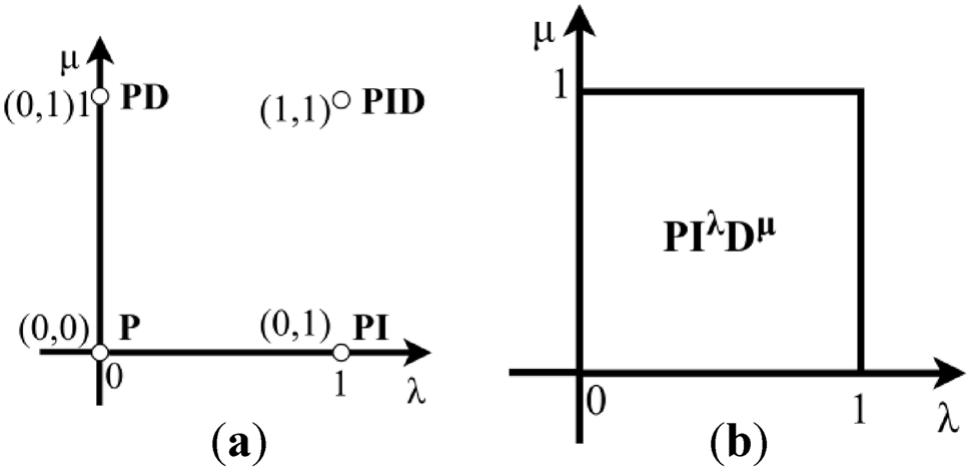
Controller representation in the λ-μ-plane: (**a**) PID controller; (**b**) FOPID controller.

As shown in Fig. 6, the FOPID regulator is a generalization for the PID regulator that extends it from a point to the whole λ-μ-plane. This generalization allows for much more freedom in controller design, resulting in deeper management for processes or plants^44^. On the other hand, the PIDD^2^ allows for higher response qualities than those of systems with PID controllers^18^. Moreover, the cascade control is one technique used to boost system performance, and it is expected that this approach will increase the system’s effectiveness^45^. Also, expanding the number of controller parameters contributes to greater flexibility in the tuning process, allowing for finer adjustments and improved adaptation to varying system dynamics and performance requirements. The CFOPI-FOPIDD^2^ controller has 10 parameters that provide more tuning knobs to shape the system’s response. These advantages have motivated us to use the proposed CFOPI-FOPIDD^2^ controller.

According to Figs. 4 and 5, $\text{ACE}(\text{s})$ serves as error input for the $C1\left(s\right)$ controller and can be expressed as Eq. (13)^1^.

13 $${\text{ACE}}_{ i}(\text{s})=\sum {\Delta P}_{tie ij}\left(s\right)+{B}_{i}\Delta {f}_{i}(s)$$

ACE_i_ consists of a linear combination of frequency and tie-line error. It monitors variations in area load and facilitates effective system regulation. A negative ACE indicates the need to increase generation, whereas a positive ACE signifies that generation should be reduced. When the ACE is zero, the system is considered to be in a steady-state equilibrium. This control can automatically modify the reference power set point. The output of $C1$ controller, is given by Eq. (14).

14 $${U}_{ C1i}(s)={ACE}_{ i}\left(s\right)\cdot {K}_{p1}+{K}_{i1}\frac{{\text{ACE}}_{ i}(s)}{{s}^{\lambda }}$$

${U}_{ C1i}(s)$ operates as the $C2\left(s\right)$ controller’s signal input. Consequently, the $C2\left(s\right)$ controller’s output (${U}_{ C2i}(c)$) is expressed as Eq. (15).

15 $${U}_{ C2i}\left(s\right)={U}_{ C1i}\left(s\right)\cdot {K}_{p2}+{K}_{i2}\frac{{U}_{ C1i}\left(s\right)}{{s}^{\lambda }}+{U}_{ C1i}\left(s\right)\cdot {K}_{d1}\cdot {s}^{\mu }+({U}_{ C1i}\left(s\right)\cdot {K}_{d1}\cdot {s}^{\mu })\cdot {K}_{d2}\cdot {s}^{\mu }$$

${U}_{ C2i}\left(s\right)$ is operating as the control input (${U}_{AGCi}(s)$) for the AGC loop. On order to obtain excellent ${U}_{AGCi}(s)$, the coefficients ${K}_{\text{p}1}$, ${K}_{\text{i}1}$, ${K}_{p2}$, ${K}_{i2}$, ${K}_{\text{d}1}$, ${K}_{d2}$, ${\uplambda }_{1}$, ${\uplambda }_{2}$, ${\mu }_{1}$ and ${\mu }_{2}$ must be carefully tuned, in order to maintain the ACE at zero to ensure that the scheduled values of system frequency and tie-line power are preserved. In this paper, these coefficients are tuned via hSBOA-PS algorithm with guidance from the proposed fitness function. Using the same procedure, the ${U}_{AVRi}\left(s\right)$ is obtained.

## The proposed fitness function

The optimum solution for a problem can be found using optimization techniques, which typically include maximizing or minimizing an objective function. An objective function is the criterion for optimization, whether it’s minimizing error, optimizing efficiency, or identifying the best combination for system’s parameters. So, the optimization process works for determining the decision variables’ values that achieve the best result in accordance with the fitness function. In the present paper, the coefficients ${K}_{\text{p}1}$, ${K}_{\text{i}1}$, ${K}_{p2}$, ${K}_{i2}$, ${K}_{\text{d}1}$, ${K}_{d2}$, ${\uplambda }_{1}$, ${\uplambda }_{2}$, ${\mu }_{1}$ and ${\mu }_{2}$ are obtained using hSBOA-PS algorithm by minimizing the objective function. The suggested objective function of this work ($\text{Obj}$) is represented by Eq. (16).

16 $${{Obj=w}_{1}\cdot SST}_{AGC\_AVR}+{w}_{2}\cdot {SOS}_{AGC}+{w}_{3}\cdot {\left|SUS\right|}_{AGC}+{w}_{4}\cdot {SOS}_{AVR}+{w}_{5}\cdot {SUS}_{AVR}+{{w}_{6}\cdot ITSE}_{AGC\_AVR}$$

17 $${\text{ITSE}}_{\text{AGC}\_\text{AVR}}=\underset{0}{\overset{t}{\int }}\left({{|\Delta f}_{i}|}^{2}+{{|\Delta f}_{j}|}^{2}{{+|\Delta P}_{tie ij}|}^{2}+{{|\Delta V}_{ti}|}^{2}+{{|\Delta V}_{tj}|}^{2}\right)\cdot t dt$$

**Fig. 7:**
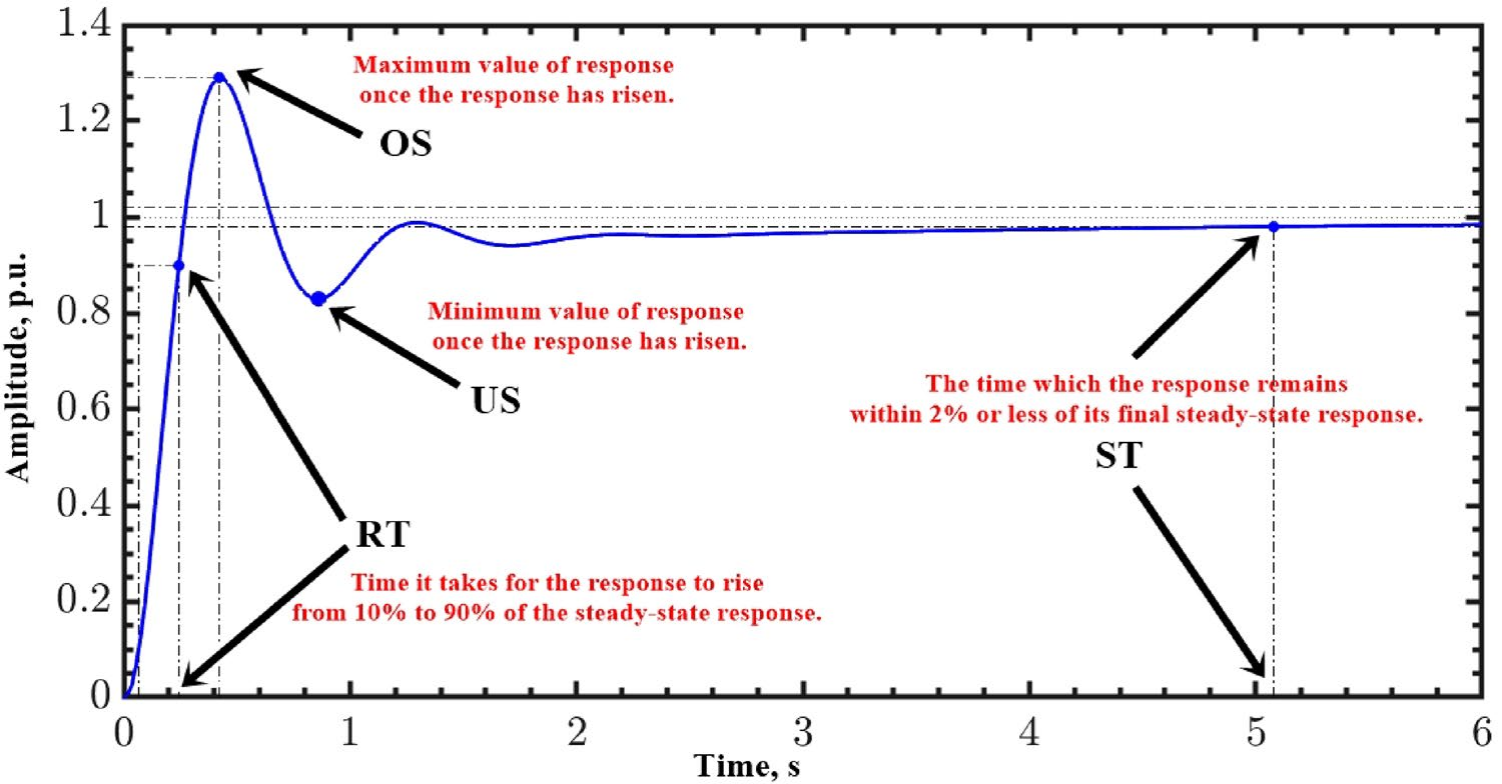
Specifications of transient response.

${\text{SST}}_{\text{AGC}\_\text{AVR}}$ represents the sum of ${\Delta f}_{i}$, ${\Delta f}_{j}$, ${\Delta P}_{tie ij}$, ${V}_{ti}$, and ${V}_{tj}$ settling times. ${\text{SOS}}_{\text{AGC}}$ and ${\text{SUS}}_{\text{AGC}}$ represents the sum of ${\Delta f}_{i}$, ${\Delta f}_{j}$, and ${\Delta P}_{tie ij}$ peak overshoot and undershoot, respectively. ${\text{SOS}}_{\text{AVR}}$ and ${\text{SUS}}_{\text{AVR}}$ represents the sum of ${V}_{ti}$ and ${V}_{tj}$ peak overshoot and undershoot, respectively. Here, ${w}_{1}$, ${w}_{2}$, ${w}_{3}$, ${w}_{4}$, ${w}_{5}$, and ${w}_{6}$ are weighting factors used to ensure that the elements of $\text{Obj}$ are competitive during the optimization procedure. Often, the optimization method focuses on minimizing the largest elements of $\text{Obj}$. Thus, the chosen weights ensure that the numerical values ​​of each element on the right-hand side of Eq. (16) are approximately of the same order of magnitude. Based on the numerous trial runs of the optimization strategy, these factors were selected as (${w}_{1}=1$, ${w}_{2}$, ${w}_{4}$, ${w}_{5}$ and ${w}_{6}$ $=1000$, and ${w}_{3}=100$). These factors make the elements on of $\text{Obj}$ approximately of the same order of magnitude. Figure 7 shows the peak overshoot ($OS$), peak undershoot ($US$), settling-time ($ST$), and rise-time ($RT$) for the step response_._

In fact, the ITAE and ITSE have been widely employed as objective functions in previous studies, where both have shown satisfactory performance. However, our empirical results indicated that including ITSE in the objective function yields slightly better performance compared to including ITSE. Thus, ITSE is included to the proposed objective function. To demonstrate this, the proposed objective function (Obj) will be compared with an objective function that incorporates ITAE (${\text{Obj}}_{2}$), which is represented by Eq. (18).

18 $${{Ob{j}_{2}=w}_{7}\cdot SST}_{AGC\_AVR}+{w}_{8}\cdot {SOS}_{AGC}+{w}_{9}\cdot {\left|SUS\right|}_{AGC}+{w}_{10}\cdot {SOS}_{AVR}+{w}_{11}\cdot {SUS}_{AVR}+{{w}_{12}\cdot ITAE}_{AGC\_AVR}$$

19 $${\text{ITAE}}_{\text{AGC}\_\text{AVR}}=\underset{0}{\overset{t}{\int }}\left(\left|\Delta {f}_{i}\right|+\left|\Delta {f}_{j}\right|+\left|\Delta {P}_{tie ij}\right|+\left|\Delta {V}_{ti}\right|+\left|\Delta {V}_{tj}\right|\right)\cdot t dt$$

Based on the numerous trial runs of the optimization method the factors selected as (${w}_{7}=1$, ${w}_{8}$, ${w}_{10}$, ${w}_{11}$ $=1000$, and ${w}_{9}=100$, and ${w}_{12}=10$). These factors make the elements on of ${\text{Obj}}_{2}$ approximately of the same order of magnitude.

## Proposed optimization strategy

The study presents a maiden algorithm. It consists of two different types of algorithms: SBOA^45^ and PS^46^. SBOA starts with a preliminary set of individuals (solutions) continuously creates new ones through repeated iterations. However, PS is considered as an optimization method that yields a one solution, which is then utilized in iterations for generation and replacement processes. This work investigates the hybridization of the SBOA and PS algorithms using the commonly referred to as relay collaboration. technique. So, in this hybrid method, SBOA is used to create the PS algorithm’s initial point ${x}_{o}$ in order to provide an excellent first answer solution. The goal from the hybrid methods is to achieve faster convergence than other methods used for dealing with the similar issue, specially, in complex problems. Also, to guarantee that the optimal solution is obtained in a few code’s runs.

### Secretary Bird Optimization Algorithm (SBOA)

The SBOA method is a recently developed optimization strategy inspired by the adaptive survival instincts exhibited by secretary birds within their native environment. It is separated by two parts: exploration and exploitation. The SBOA’s exploration part mimics secretary birds pursuing snakes, whereas the exploitation part mimics secretary birds flight fleeing predators^45^.

#### SBOA initialization

The positions occupied by each secretary bird in the search space represent the decision variables’ values. Here the decision variables are ${K}_{\text{p}1}$, ${K}_{\text{i}1}$, ${K}_{p2}$, ${K}_{i2}$, ${K}_{\text{d}1}$, ${K}_{d2}$, ${\uplambda }_{1}$, ${\uplambda }_{2}$, ${\mu }_{1}$ and ${\mu }_{2}$. Before phase one, the initial solutions need to be created. So, the Secretary Birds’ positions are initially set at random using Eq. (20)^45^.

20 $${X}_{i,j}=l{b}_{j}+r\times \left(u{b}_{j}-l{b}_{j}\right),i=\text{1,2},\dots ,N,j=\text{1,2},\dots , \, {\text{Dim}}$$

The ${i}^{th}$ secretary bird’s place is defined by ${X}_{i}$, ${X}_{i,j}$ indicates the value of the ${j}^{th}$ decision variable got by the ${i}^{th}$ secretary bird. The lowest and upper limits of decision variables are expressed by $l{b}_{j}$ and $u{b}_{j}$, and $r$ is a value randomly chosen within the range of 0 and 1. $N$ indicates the total secretary birds’ number, and $Dim$ indicates the total decision variables’ number.

The possible solutions of decision variables ${K}_{\text{p}1}$, ${K}_{\text{i}1}$, ${K}_{p2}$, ${K}_{i2}$, ${K}_{\text{d}1}$, ${K}_{d2}$, ${\uplambda }_{1}$, ${\uplambda }_{2}$, ${\mu }_{1}$ and ${\mu }_{2}$ that are generated at random can be represented by Eq. (21)^45^.

21 $$X={\left[\begin{array}{cccccc}{x}_{\text{1,1}}& {x}_{\text{1,2}}& \cdots & {x}_{1,j}& \cdots & {x}_{1,{\text{Dim}} \, }\\ {x}_{\text{2,1}}& {x}_{\text{2,2}}& \cdots & {x}_{2,j}& \cdots & {x}_{2, \text{Dim}}\\ \vdots & \vdots & \ddots & \vdots & \ddots & \vdots \\ {x}_{i,1}& {x}_{i,2}& \cdots & {x}_{i,j}& \cdots & {x}_{i,\text{Dim}}\\ \vdots & \vdots & \ddots & \vdots & \ddots & \vdots \\ {x}_{N,1}& {x}_{N,2}& \cdots & {x}_{N,j}& \cdots & {x}_{N, \text{Dim}}\end{array}\right]}_{N\times \, {\text{Dim}}}$$

The decision variables’ values that suggested via every secretary bird are employed to assess the objective function ($\text{Obj}$), which can be represented by a vector as Eq. (22), where ${\text{Obj}}_{\text{i}}$ is the value of $\text{Obj}$ that the ${i}^{th}$ secretary bird obtained^45^.

22 $$\text{Obj}={\left[\begin{array}{c}{\text{Obj}}_{1}\\ \vdots \\ {\text{Obj}}_{i}\\ \vdots \\ {\text{Obj}}_{N}\end{array}\right]}_{N\times 1}={\left[\begin{array}{c}\text{Obj}\left({X}_{1}\right)\\ \vdots \\ \text{Obj}\left({X}_{i}\right)\\ \vdots \\ \text{Obj}\left({X}_{N}\right)\end{array}\right]}_{N\times 1}$$

The solution that obtains the best value of $\text{Obj}$ is selected to start phase 1. In phases 1 and 2, each iteration entails determining the optimal possible solutions (secretary bird locations) as well as updating the $\text{Obj}$ values.

#### Phase 1: exploration

23 $$\text{While} ct<\frac{1}{3}mt,{x}_{i,j}^{\text{new }P1}={x}_{i,j}+\left({x}_{\text{random\_1 }}-{x}_{\text{random\_2 }}\right)\times {R}_{1}$$

24 $${X}_{i}=\left\{\begin{array}{c}{X}_{i}^{{\text{new}},P1}, \, {\text{if}} \, { \text{Obj}}_{i}^{{\text{new}},P1}<{\text{Obj}}_{i}\\ {X}_{i}, \, {\text{else}}\end{array}\right.$$

This phase simulates the secretary birds’ foraging strategy. It includes three stages which are: locating prey, exhausting its energy, and attacking it. The stages are characterized by three equal time intervals: $ct<\frac{1}{3}mt, \frac{1}{3}mt<ct<\frac{2}{3}mt\text{ and }\frac{2}{3}mt<ct<mt$. The secretary birds’ locations, as well as the best possible solution, are updated at each stage of the exploration phase. The secretary bird’s location through the search for prey stage could be computationally defined utilizing Eqs. (23) and (24)^45^.

$ct$ and $mt$ are the current iteration and maximum iteration numbers. ${x}_{\text{random\_1}}$ and ${x}_{\text{random\_2}}$ are the first stage iteration’s random potential solutions, while the new potential of the ${i}^{th}$ secretary bird is indicated via ${X}_{i}^{{\text{new}},P1}$. $R1$ is a $1\times {\text{Dim}}$ array created at random inside the range [0, 1]. ${x}_{i,j}^{\text{new }P1}$ represents the ${j}^{th}$ dimension’s value of ${X}_{i}^{{\text{new}},P1}$, while ${\text{Obj}}_{i}^{{\text{new}},P1}$ represents its $\text{Obj}$ value.

The secretary bird’s location during the diminishing prey’s energy stage could be computationally determined via Eqs. (25) and (26)^45^.

25 $$\text{While} \frac{1}{3}mt<ct<\frac{2}{3}mt,{x}_{i,j}^{\text{new }P1}={x}_{\text{bess }}+exp((ct/mt)\wedge 4)\times (RB-0.5)\times \left({x}_{\text{best }}-{x}_{i,j}\right)$$

26 $${X}_{i}=\left\{\begin{array}{l}{X}_{i}^{\text{new },P1}, \, {\text{if}} \, {Obj}_{i}^{\text{new },P1}<{Obj}_{i}\\ {X}_{i},\text{ else}\end{array}\right.$$

$RB=randn(1, \, {\text{Dim}} \, )$ that is a randomly $1\times {\text{Dim}}$ array inside a typical normal distribution having mean of zero and standard deviation of one. ${x}_{\text{bess}}$ is the best available location of the secretary bird.

27 $$\text{While }ct>\frac{2}{3}mt,{x}_{ij}^{new}P1={x}_{\text{best }}+\left(\left(1-\frac{ct}{mt}\right)\wedge \left(2\times \frac{ct}{mt}\right)\right)\times {x}_{ij}\times RL$$

28 $${X}_{i}=\left\{\begin{array}{l}{X}_{i}^{\text{new },P1}, \, {\text{if}} \, {\text{Obj}}_{i}^{\text{new },P1}<{\text{Obj}}_{i}\\ {X}_{i}, \, {\text{else}}\end{array}\right.$$

29 $$RL=0.5\times s\times \frac{u\times \sigma }{|v{|}^{\frac{1}{\eta }}}$$

30 $$\sigma ={\left(\frac{\Gamma (1+\eta )\times sin\left(\frac{\pi \eta }{2}\right)}{\Gamma \left(\frac{1+\eta }{2}\right)\times \eta \times 2\left(\frac{\eta -1}{2}\right)}\right)}^{\frac{1}{\eta }}$$

The secretary bird’s location through the attacking prey stage could be mathematically defined utilizing Eqs. (27) and (28)^45^.

$RL$ is the weighted Levy flight which is used to improv the accuracy of the optimization and is obtained using Eq. (29)^45^. The fixed values for $s$ and $\eta$ are 0.01 and 1.5. The values for $u$ and $v$ are selected at random from the interval [0, 1]. $\sigma$ is obtained using Eq. (30)^45^, $\Gamma$ is the gamma function and $\eta =1.5$.

#### Phase 2: exploitation

This phase simulates the secretary bird’s strategy for escaping from the predators such as eagles and foxes. It includes two stages: flee or fly away and camouflage by the environment. The idea behind the development of SBOA is that the likelihood of either of these two stages occurring is equal. Equation (31) is used for computationally model both of the evasive methods which secretary birds performed, whereas Eq. (32) is used to specify this updated condition^45^.

31 $${x}_{i,j}^{\text{new }P2}=\left\{\begin{array}{c}{P}_{1}:{x}_{\text{best }}+(2\times RB-1)\times {\left(1-\frac{ct}{mt}\right)}^{2}\times {x}_{i,j},\text{ if }r\text{ and }<{r}_{i}\\ {P}_{2}:{x}_{i,j}+{R}_{2}\times \left({x}_{\text{random }}-K\times {x}_{i,j}\right),\text{ else}\end{array}\right.$$

32 $${X}_{i}=\left\{\begin{array}{l}{X}_{i}^{\text{new },P2},\text{ if }{Obj}_{i}^{\text{new },P2}<{Obj}_{i}\\ {X}_{i},\text{ else}\end{array}\right.$$

33 $$K=\text{round}(1+\text{rand}(\text{1,1}))$$

$r$ = 0.5. ${R}_{2}$ is a random creation of $1 \times \text{ Dim}$ array from the normal distribution. ${x}_{\text{random}}$ is the current iteration’s random potential solution. $K$ is a random choice of an integer 1 or 2, that could be obtained by employing Eq. (33)^45^. $\text{rand}(\text{1,1})$ means generating a random number between 0 and 1.

### Pattern Search (PS)

PS approaches are considered a kind of direct search approach used to solve issues in nonlinear optimization^46^. It is computationally efficient, has a straightforward notion, and is simple to implement. Furthermore, it has a versatile and balanced operator to improve global search and optimize local search^47^. It produces a sequence of points, which could or could not reach the optimum solution. It starts by encircling the initial points with a collection of points known as a mesh. Here, the initial points are established via SBOA. The mesh is generated via the addition of an initial or current point to a scalar that is a multiple for a group of vectors known as a pattern. A point in the mesh is selected as the current point for the subsequent iteration (poll) if its $\text{Obj}$ value is the best.

**Fig. 8:**
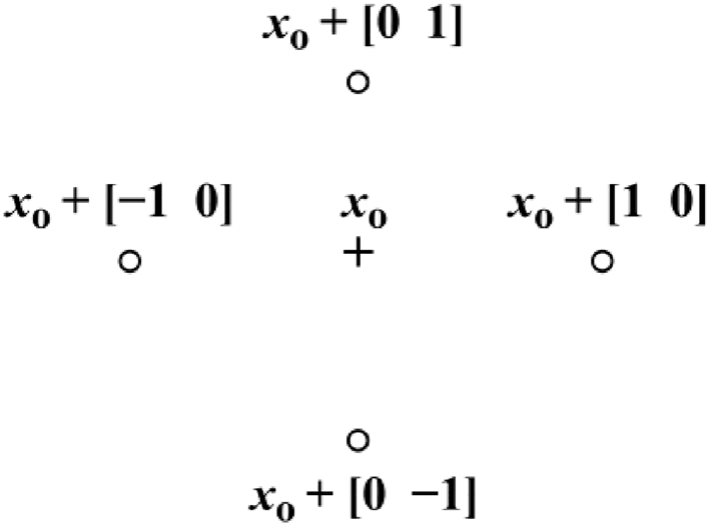
Pattern search mesh points and the pattern.

In the initial iteration, the mesh size is set to 1 and the pattern vectors are created as $[0 1]+{x}_{o}$, $[1 0]+{x}_{o}$, $[-1 0]+{x}_{o}$, and $[0 -1]+{x}_{o}$. Additionally, new mesh points are calculated as seen in Fig. 8. After that, the created trial points’ $\text{Obj}$ is computed until a value less than ${x}_{o}$ occurs. The poll is successful if there is a position where $\text{Obj}$ (${x}_{1}$) < $\text{Ob}$ j (${x}_{o}$), and the algorithm designates this point as a source point. After a successful poll, PS method proceeds to poll two by multiplying the existing mesh size by two (referred to as the expansion factor). So, following new points are $:2\times [0 1]+{x}_{1}$, $2\times [1 0]+{x}_{1}$, $2\times [-1 0]+{x}_{1}$, and $2\times \left[0 -1\right]+{x}_{1}$. Once a value less than ${x}_{1}$ is generated, ${x}_{2}$ is defined, the size of the mesh increases by two, and the iterations proceed. On the other hand, the size of the mesh decreases via multiplying by a reduction weigh, and the current point remains unchanged if the poll fails at any point (i.e., $\text{Obj}$ (${x}_{1}$) > $\text{Obj}$ (${x}_{o}$)). This operation is repeated until the minimum $\text{Obj}$ is reached or a terminating condition is satisfied.

### Hybrid SBOA and PS (hSBOA-PS) based AGC-AVR

**Fig. 9:**
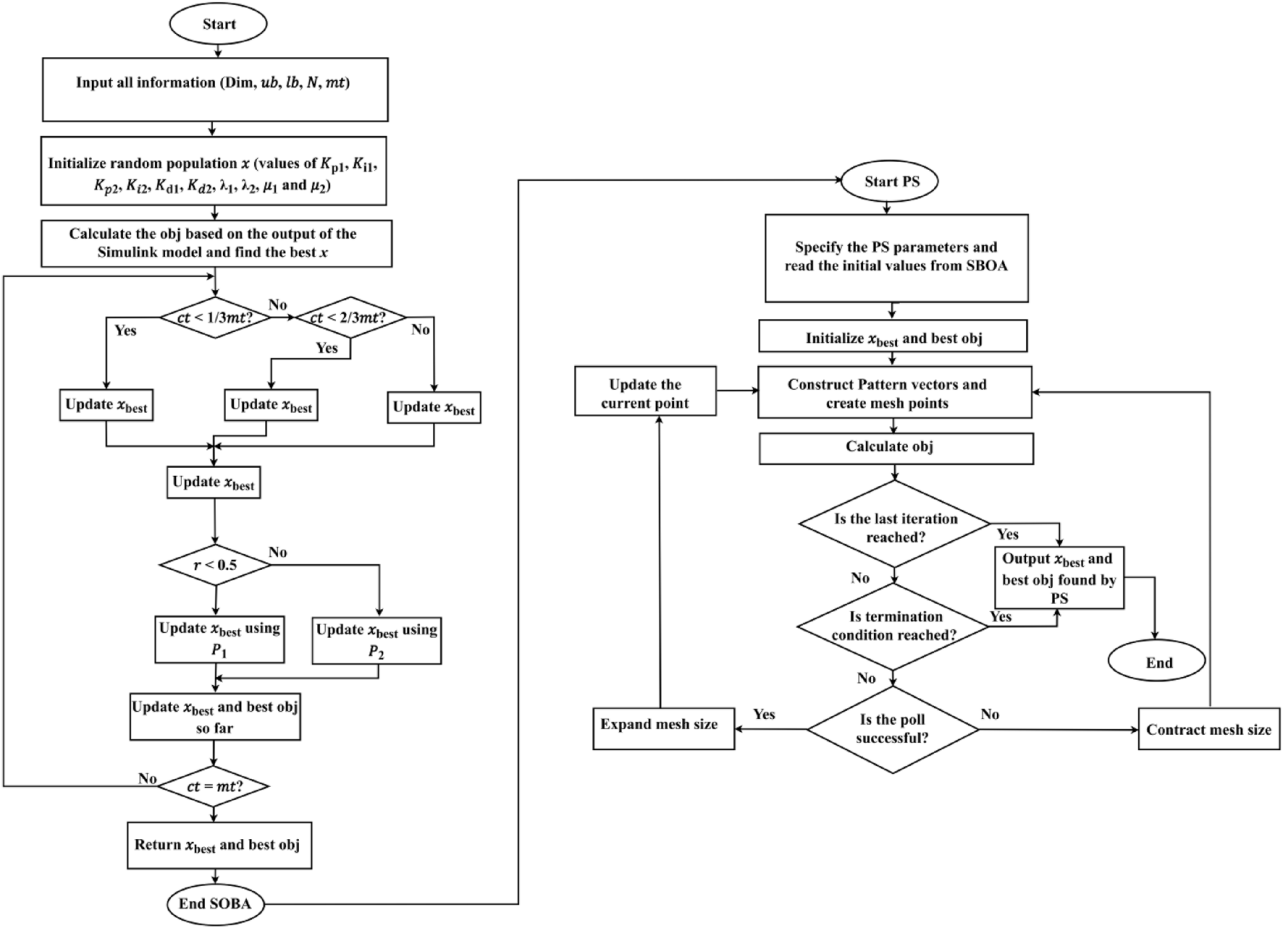
The hSBOA-PS’s flow chart.

**Fig. 10:**
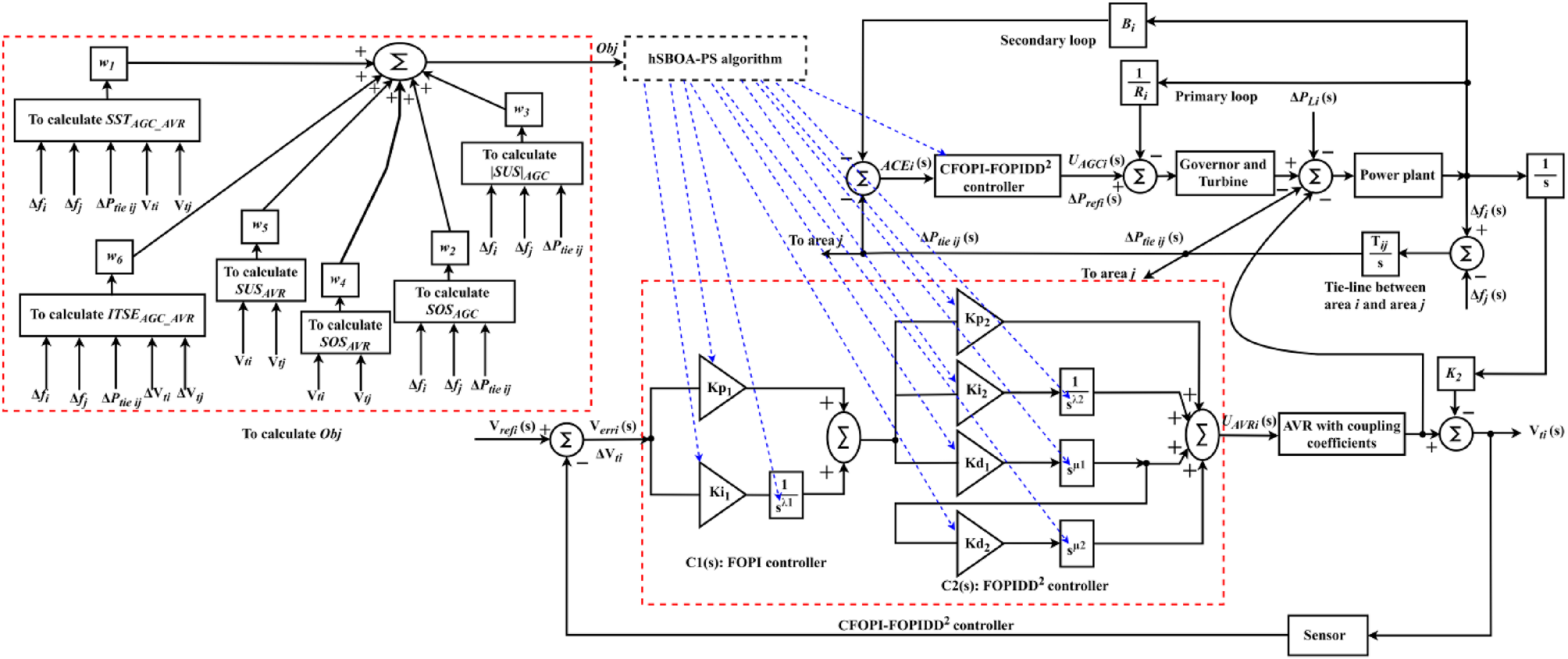
An explanation of the suggested tuning method.

Algorithm 1 shows the *h*SBOA-PS’s pseudocode for getting the optimal ${K}_{\text{p}1}$, ${K}_{\text{i}1}$, ${K}_{p2}$, ${K}_{i2}$, ${K}_{\text{d}1}$, ${K}_{d2}$, ${\uplambda }_{1}$, ${\uplambda }_{2}$, ${\mu }_{1}$ and ${\mu }_{2}$ and Fig. 9 represents the hSBOA-PS’s flow chart. Figure 10 represents the general structure for the suggested tuning strategy. The test systems are carried out utilizing the Simulink-MATLAB program. The Simulink software is used for implementing the test systems. The hSBOA-PS code is done in MATLAB script file, and $\text{Obj}$ is computed via hSBOA-PS using the Simulink model results. The hSBOA-PS code is ran several runs, and the best run (which obtained the best $\text{Obj}$) is chosen as the optimum values of ${K}_{\text{p}1}$, ${K}_{\text{i}1}$, ${K}_{p2}$, ${K}_{i2}$, ${K}_{\text{d}1}$, ${K}_{d2}$, ${\uplambda }_{1}$, ${\uplambda }_{2}$, ${\mu }_{1}$ and ${\mu }_{2}$.

**Algorithm 1:**
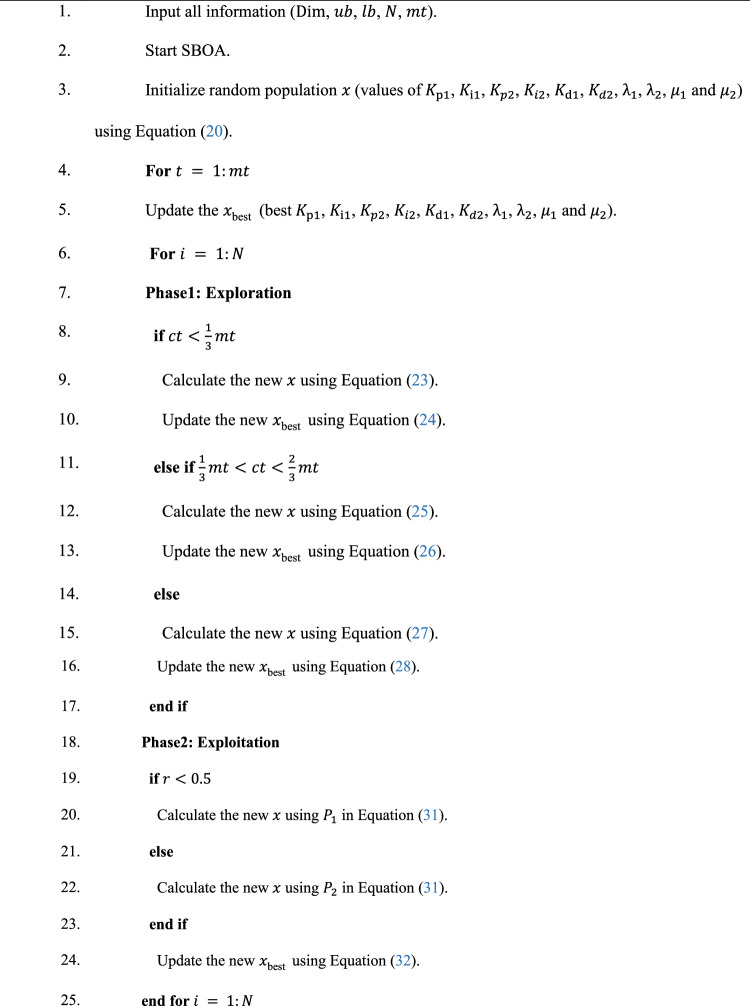

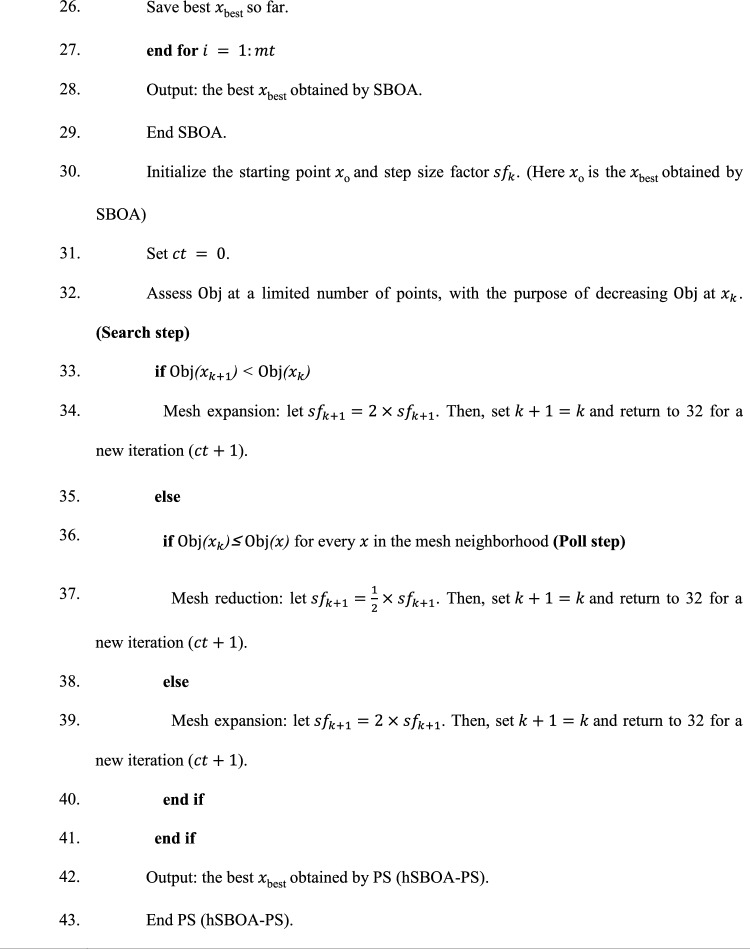
Pseudocode of hSBOA-PS.

### Computational complexity hSBOA-PS

Different algorithms require varying amounts of time to optimize the same problems; thus, evaluating an algorithm’s computational complexity is essential for assessing its execution efficiency^45^. According to the standard rules for analyzing time complexity using Big-O notation^45^, the time complexity for the random initialization of the population is $O(N)$. During the solution update phase, the computational complexity is $O(mt\times N)+O(mt\times N\times Dim)$, accounting for both identifying the best solutions and updating the positions of all individuals. Consequently, the overall computational complexity of the SBOA algorithm can be expressed as $O(N\times (mt\times Dim+1))$. PS is typically a local search that evaluates neighboring points of a current solution to find an improvement. Thus, the computational complexity of the PS can be expressed as $O(m{t}_{PS}\times Dim)$. $m{t}_{PS}$ is the maximum number of PS iterations. Here, the PS is applied after SBOA, so the computational complexity hSBOA-PS can be expressed $O(N\times (mt\times Dim+1))+O(m{t}_{PS}\times Dim)$. Also, the iterations of hSBOA-PS are equally distributed between the SBOA and PS phases.

## Simulation results and discussion for test system-1

**Fig. 11:**
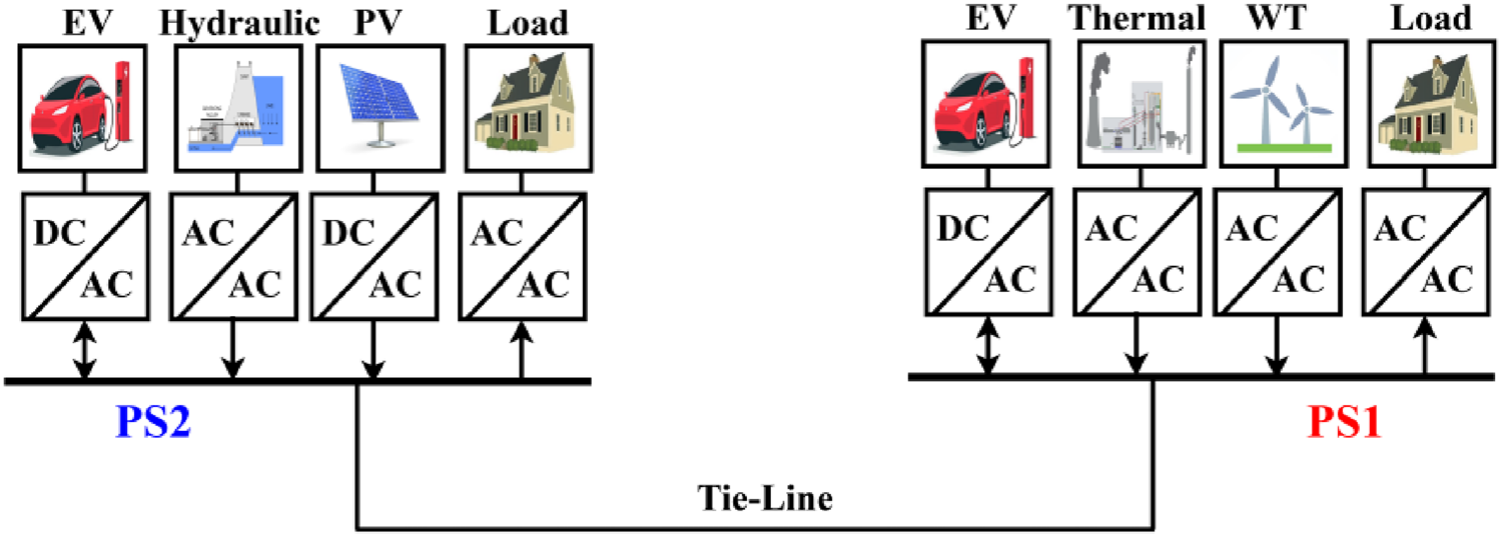
Hybrid dual-area IPS under study.

**Fig. 12:**
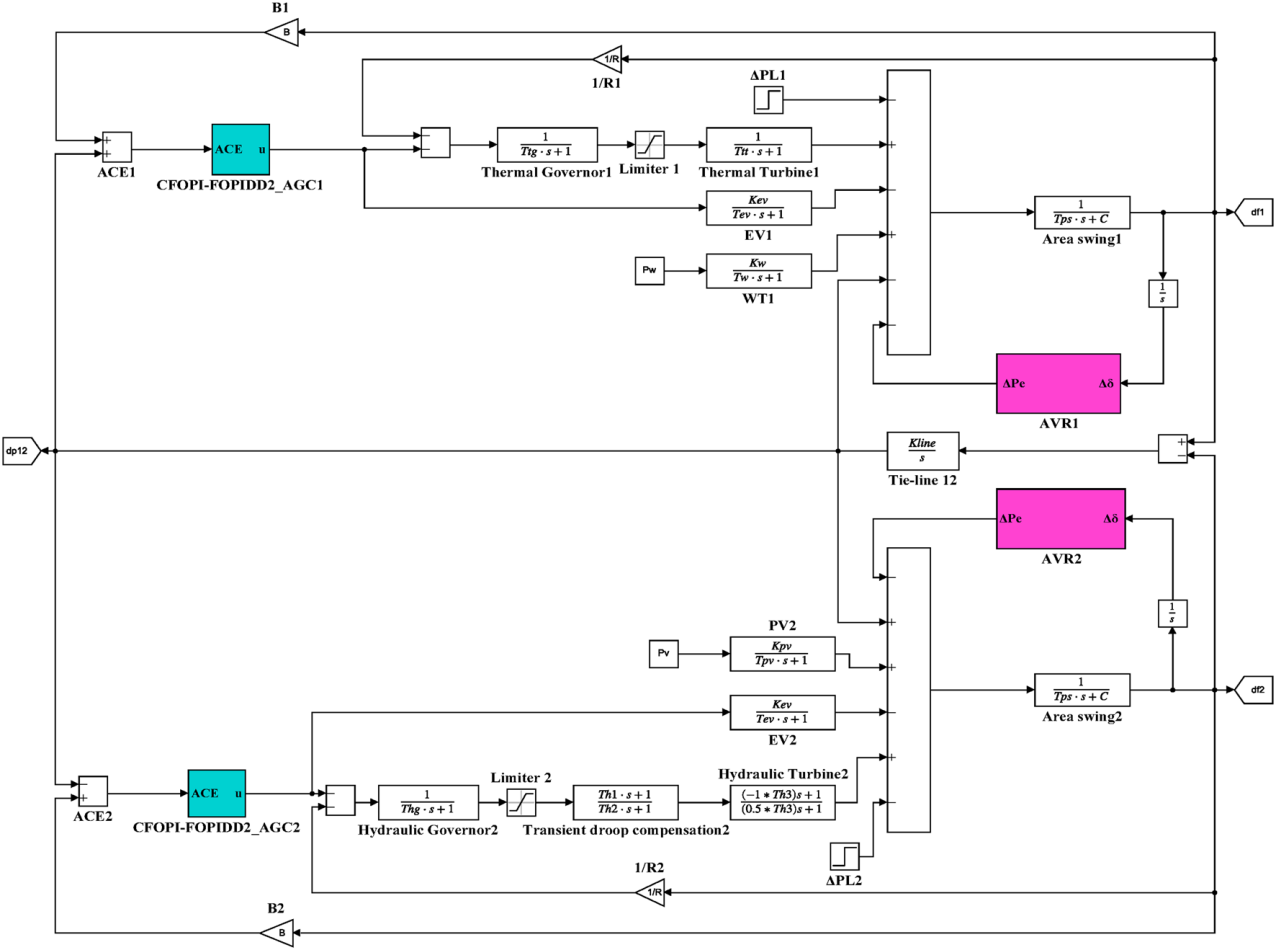
AGC-AVR Simulink model of the dual-area IPS under study.

Here, the MATLAB software simulation is used for evaluating the effectiveness of the proposed CFOPI–FOPIDD^2^ controller using hSBOA-PS algorithm under various scenarios. So, the suggested control technique is investigated on a hybrid dual-region IPS that is shown in Fig. 11. Area 1 contains thermal unit, wind farm (WT), and EVs, while area 2 contains hydraulic unit, PV, and EVs. Table 2 illustrates the transfer function for the IPS components, and the AGC-AVR Simulink model is depicted in Fig. 12.

**Table 2:** Hybrid dual-area IPS under study^38^.

Unit	Model	Transfer Function	Parameter Value
Non-reheat Thermal	Governor	$\frac{1}{{T}_{tg}\cdot s+1}$	${T}_{tg}=0.08\text{ s}$
	Limiter	–	$\text{Min}=-0.5$ and $\text{Max}=0.5$
	Turbine	$\frac{1}{{T}_{tt}\cdot s+1}$	${T}_{tt}=0.3\text{s}$
Hydraulic	Governor	$\frac{1}{{T}_{hg}\cdot s+1}$	${T}_{hg}=41.6 s$
	Limiter	–	$\text{Min}=-0.5$ and $\text{Max}=0.5$
	Transient droop compensation	$\frac{{T}_{h1}\cdot s+1}{{T}_{h2}\cdot s+1}$	${T}_{h1}=5\text{ s}$ and ${T}_{h2}=0.513\text{ s}$
	Turbine	$\frac{-{T}_{h3}\cdot s+1}{{0.5.T}_{h3}\cdot s+1}$	${T}_{h3}=1\text{ s}$
AVR	Amplifier	$\frac{{K}_{a}}{{T}_{a}\cdot s+1}$	${K}_{a}=10$ and ${T}_{a}=0.1\text{ s}$
	Exciter	$\frac{{K}_{e}}{{T}_{e}\cdot s+1}$	${K}_{e}=1$ and ${T}_{e}=0.4\text{ s}$
	Generator	$\frac{{K}_{g}}{{T}_{g}\cdot s+1}$	${K}_{g}=1$ and ${T}_{g}=1\text{ s}$
	Sensor	$\frac{{K}_{s}}{{T}_{s}\cdot s+1}$	${K}_{s}=1$ and ${T}_{s}=0.01\text{ s}$
	AVR coupling coefficients	${K}_{1}$, ${K}_{2}$, ${K}_{3}$, ${K}_{4}$,${K}_{5}$	$1.5$, $0.3$, $0.1$, $1.4$,$0.5$
Other models	PV	$\frac{{K}_{pv}}{{T}_{pv}\cdot s+1}$	${K}_{pv}=1$ and ${T}_{pv}=1.3\text{ s}$
	WT	$\frac{{K}_{w}}{{T}_{w}\cdot s+1}$	${K}_{w}=1$ and ${T}_{w}=1.5\text{ s}$
	EV	$\frac{{K}_{ev}}{{T}_{ev}\cdot s+1}$	${K}_{ev}=1$ and ${T}_{ev}=1\text{ s}$
	Area swing	$\frac{1}{{T}_{ps}\cdot s+\text{C}}$	$C=0.00833$ and ${T}_{ps}=0.1667\text{ s}$
	T-line	$\frac{{K}_{LINE}}{s}$	${K}_{LINE}=0.5434$
	Frequency bias	$\text{B}$	$0.4249 \text{MW}/\text{Hz}$
	Speed droop	$\text{R}$	2.4 Hz/MW

### hSBOA-PS testing

**Fig. 13:**
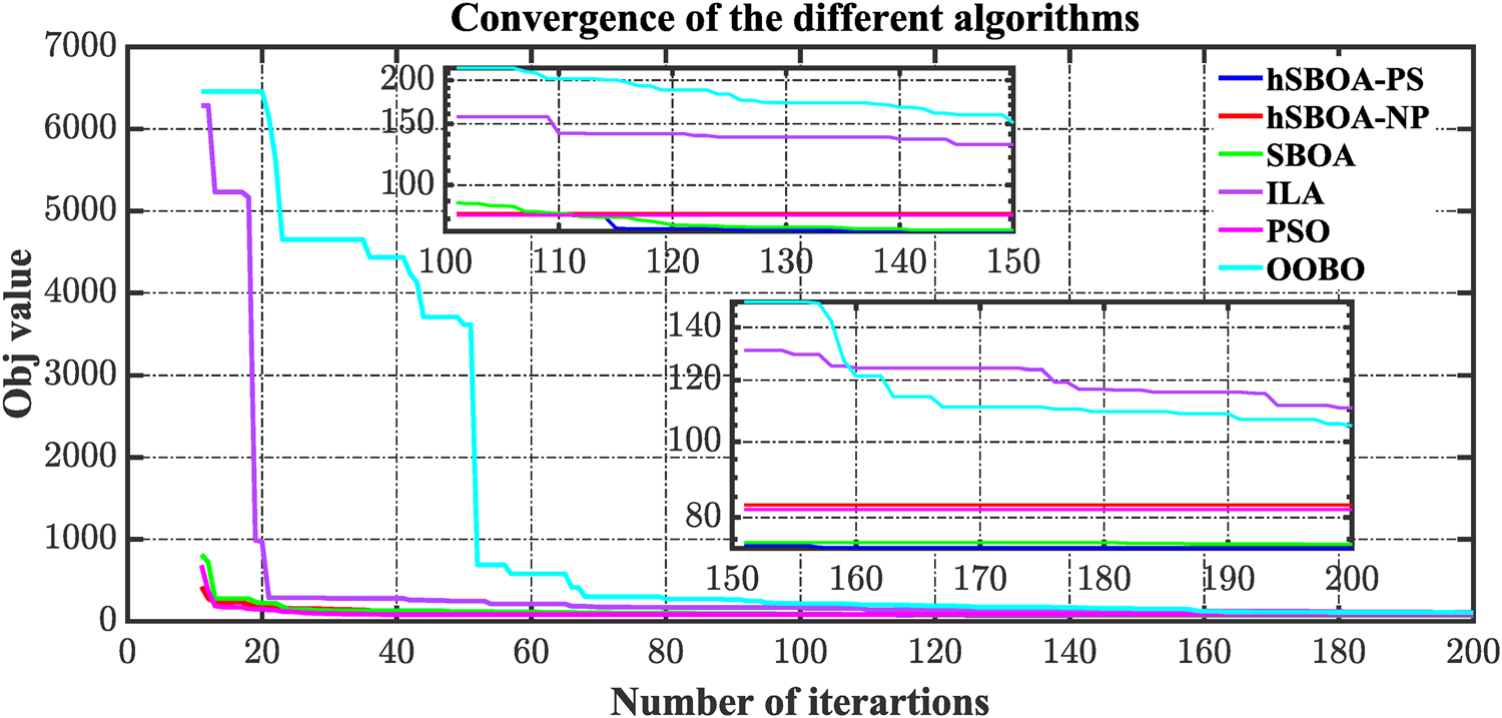
The tendency convergence of the different algorithms.

The purpose of this investigation is to show the ability of hSBOA-PS for finding the suitable ${K}_{\text{p}1}$, ${K}_{\text{i}1}$, ${K}_{p2}$, ${K}_{i2}$, ${K}_{\text{d}1}$, ${K}_{d2}$, ${\uplambda }_{1}$, ${\uplambda }_{2}$, ${\mu }_{1}$ and ${\mu }_{2}$ that obtain the minimum $\text{Obj}$ with fast converge. The optimization is done on the basis of the circumstance of 5% step load disturbance (SLD) occurs in area two at t = 0 s with a time-fixed desired voltage (TFTV) equals to 1 p.u. Additionally, the performance of hSBOA-PS is compared with hSBOA-nonlinear programming (NP), SBOA, the incomprehensible but intelligible-in-time logics technique (ILA), PSO, and OOBO. The population and iterations are set to 20 and 200, respectively, for both algorithms, and separate runs are carried out. Given that the total number of iterations is set to 200, the hSBOA-PS algorithm transitions to the PS phase starting from iteration 101. Because the iterations are distributed equally between the SOBA and PS. Figure 13 illustrates the convergence tendency of the different algorithms, which demonstrates the evolution of the optimization procedure to achieve the lowest value of Obj, thereby selecting the best-tuned **C**FOPI-FOPIDD^2^ controller. Based on Fig. 13, the proposed hSBOA-PS has the lowest values of $\text{Obj}$ over time. So, hSBOA-PS surpassed the other algorithms.

Tables 3 and 4 present a statistical analysis of the results obtained from the different algorithms. Based on the results presented, the hSBOA-PS demonstrates good statistical performance compared to the other methods. On the other hand, based on the execution time, the hSBOAPS algorithm achieves a 2.53% reduction in computational complexity compared to the SBOS algorithm.

**Table 3:** Statistical comparison of different algorithms using $\text{Obj}$.

Metric	hSBOA-PS	hSBOA-NP	SBOA	ILA	PSO	OOBO
Best	73.01	82.97	73.96	110.53	81.91	104.87
Median	82.90	82.97	87.04	157.23	81.91	216.93
Mean	97.20	101.46	107.96	401.95	91.98	1235.2
Worst	421.98	421.98	812.89	6285.6	682.73	6457.7
Standard deviation	43.86	41.74	80.45	1077.5	50.94	1991.8
Execution time (s)	51,480.77	57,962.96	52,817.95	57,738.43	25,246.44	25,637.50

**Table 4:** Statistical comparison of different algorithms using $\text{Obj}$ values over iterations 101–200.

Metric	hSBOA-PS	hSBOA-NP	SBOA	ILA	PSO	OOBO
Best	73.01	82.97	73.96	110.53	81.91	104.87
Median	73.69	82.97	74.29	130.82	81.91	150.90
Mean	74.86	82.97	76.27	130.61	81.91	150.55
Worst	82.97	82.97	89.09	157.23	81.91	216.94
Standard deviation	3.18	1.0634 × 10^−4^	3.85	12.57	5.9707 × 10^−4^	37.97

Table 5 presents the transient performance for different objective functions using hSBOA-PS. Table 5 clearly indicates that utilizing $\text{Obj}$ as the objective function yields a little superior performance compared to ${\text{Obj}}_{2}$.

**Table 5:** Transient performance for different objective functions using hSBOA-PS.

Performance index	${\text{SST}}_{\text{AGC}\_\text{AVR}}$	${\text{SOS}}_{\text{AGC}}$	${\text{SUS}}_{\text{AGC}}$	${\text{SOS}}_{\text{AVR}}$	${\text{SUS}}_{\text{AVR}}$
$\text{Obj}$	**7.8722**	0.33892	**0.019465**	**0**	**0**
${\text{Obj}}_{2}$	8.2815	**0.30147**	0.017758	**0**	5.6579 × 10^−5^

The figures in bold are the best.

### Case studies simulation results

Here, the efficiency of the suggested hSBOA-PS-CFOPI-FOPIDD^2^ controller on IPS depicted in Fig. 12 is tested using simulation in MATLAB program for different conditions. The elements influencing the AGC-AVR’s functionality and cause disturbances in the grid are load fluctuations, changes in the generating units’ power, CTD, and uncertainty of parameters. So, the following scenarios are examined:

- Scenario 1: SLD and TFDV impact.
- Scenario 2: Time-varying CTD impact.
- Scenario 3: Random load disturbance (RLD) and time-varying desired voltage (TVDV) impact.
- Scenario 4: Solar irradiance variation impact.
- Scenario 5: Wind speed variation impact.
- Scenario 6: Consideration of all RESs as well as RLD and TVDV.
- Scenario 7: Consideration of AVR system as DISO.
- Scenario 8: System parameter variation impact.

The parameters of CFOPI-FOPIDD^2^ controller which are obtained in the first scenario will be used in the rest of the scenarios to demonstrate the controller’s adaptivity. Also, the performance CFOPI-FOPIDD^2^ controller will be compared with FOPID and PID controllers. It is important to mention that in these simulations, the solver ode4 (Runge–Kutta) is utilized with a fixed-step size of 0.001.

#### Scenario 1: SLD and TFDV impact

The change in load in the IPS is typically executed through the connection or disconnection of fixed values. So, the load changes are modeled using the step input. Also, the ${V}_{ref}$ or the desired voltage is modeled by step input. In this scenario, a 5% SLD is occurred in region two at t = 0 s. Also, the ${V}_{ref}$ is considered as 1 p.u. for both areas. Additionally, the wind and solar units’ electricity generation are inaccessible. Table 6 lists the controllers’ settings which hSBOA-PS achieved. Table 7 contains the dynamic specifications for the Test System-1 utilizing various controllers. According to Table 7, the hSBOA-PS-CFOP-FOPIDD^2^ controller demonstrated its supremacy over other controllers for the majority of the dynamic specifications. Furthermore, the hSBOA-PS-CFOP-FOPIDD2 controller improves ITSE by 60.5% compared to the DO-FOPI-PIDD^2^ controller, and by 81% compared to the adaptive fuzzy PID (AFPID) controller.

**Table 6:** Suggested parameters for the different controllers gotten via hSBOA-PS.

Controller	${K}_{\text{p}1}$/${K}_{\text{p}}$	${K}_{\text{i}1}$/${K}_{\text{i}}$	${K}_{p2}$	${K}_{i2}$	${K}_{\text{d}1}$/${K}_{\text{d}}$	${K}_{d2}$	${\lambda }_{1}$	${\uplambda }_{2}$	${\mu }_{1}$	${\mu }_{2}$
AGC										
CFOPI-FOPIDD^2^	1.9998	1.9991	2	1.8916	2	0.0989	0.2172	0.7254	0.9500	0.9074
FOPID	0.1703	1.4498	–	–	2	–	0.4678	–	0.9500	–
PID	2	0.8315	–	–	2	–	–	–	–	–
AVR										
CFOPI-FOPIDD^2^	1.1329	0.9006	1.1260	1.4605	1.3715	0.0998	0.8573	0.2014	0.9500	0.9500
FOPID	0.5214	0.8396	–	–	0.9681	–	0.7043	–	0.9499	–
PID	2	0.3415	–	–	0.5797	–	–	–	–	–

**Table 7:** AGC-AVR dynamic characteristics utilizing various controllers during first scenario.

Performance index	hSBOA-PS-CFOPI-FOPIDD^2^ (proposed)	hSBOA-PS-FOPID	hSBOA-PS-PID	hSBOA-PS-AFPID	DO-FOPI-PIDD^2^^38^	DO-FOPD-PIDD^2^^38^	TD-TI-PIDD^2^^38^
$$ST$$ (s)							
${\Delta f}_{1}$	2.2749	2.5934	4.7302	2.5126	**2.1**	6	10
${\Delta f}_{2}$	**1.8671**	2.2216	2.5065	2.1746	3.2	8.5	11
${\Delta P}_{tie12}$	3.3511	12.7717	2.9520	**2.3049**	2.8	10	13
${Vt}_{1}$	**0.1917**	2.2802	1.3036	1.9114	0.67	1.7	1.6
${Vt}_{2}$	**0.1873**	2.2990	1.2904	1.9139	1.22	1.16	1.38
$$RT$$ (s)							
${Vt}_{1}$	**0.0996**	0.1877	0.5687	0.1664	0.14	0.84	1.02
${Vt}_{2}$	**0.0995**	0.1871	0.5456	0.1661	0.172	0.17	0.19
$$OS$$ (Hz)							
${\Delta f}_{1}$	**5.4665 × 10**^**−6**^	9.67 × 10^−4^	0.0071	0.0036	1 × 10^−5^	0.004	0.02
${\Delta f}_{2}$	8.0583 × 10^−4^	0.0184	0.0079	0.0041	**0**	**0**	**0**
$$OS$$ (p.u.)							
${\Delta P}_{tie12}$	0.0169	0.0568	0.0476	0.0327	**0.006**	0.002	0.011
${Vt}_{1}$	**1**	**1**	**1**	**1**	1.059	**1**	1.014
${Vt}_{2}$	**1**	**1**	**1**	**1**	1.052	1.049	1.059
$$US$$ (Hz)							
${\Delta f}_{1}$	**− 0.1189**	− 0.3105	− 0.2610	− 0.1805	− 0.133	− 0.513	− 0.42
${\Delta f}_{2}$	− 0.2198	− 0.5948	− 0.3972	− 0.2811	**− 0.147**	− 0.31	− 0.24
$$US$$ (p.u.)							
${\Delta P}_{tie12}$	− 2.4461 × 10^−4^	**0**	− 8.39 × 10^−4^	− 2.92 × 10 − ^4^	− 0.006	− 0.081	0.011
${Vt}_{1}$	**1**	0.8289	**1**	**1**	–	–	–
${Vt}_{2}$	**1**	0.8396	**1**	**1**	–	–	–
$\text{ITSE}$	**0.0135**	0.2003	0.2204	0.07311	0.0342	0.2136	0.2357

The figures in bold are the best.

#### Scenario 2: Time-varying CTD impact

**Fig. 14:**
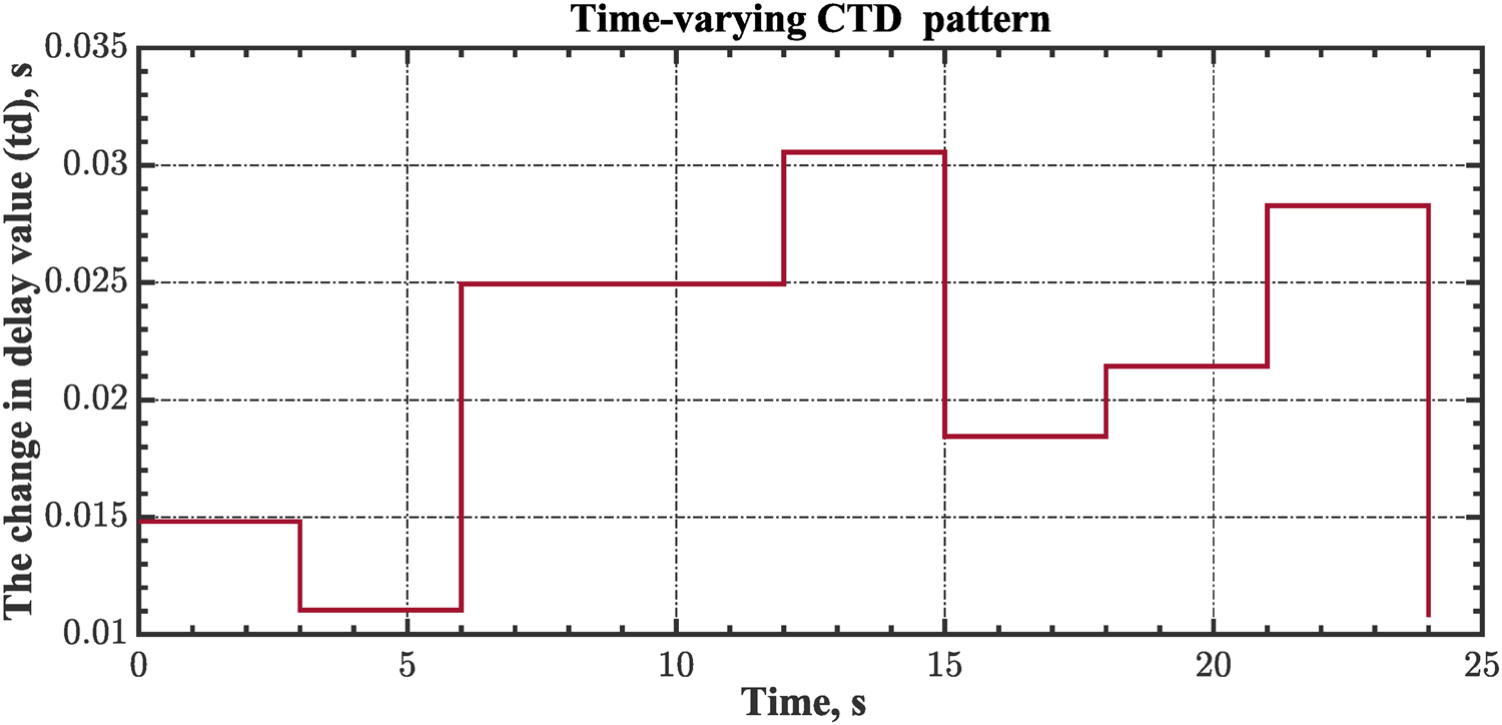
The time-varying CTD pattern.

**Fig. 15:**
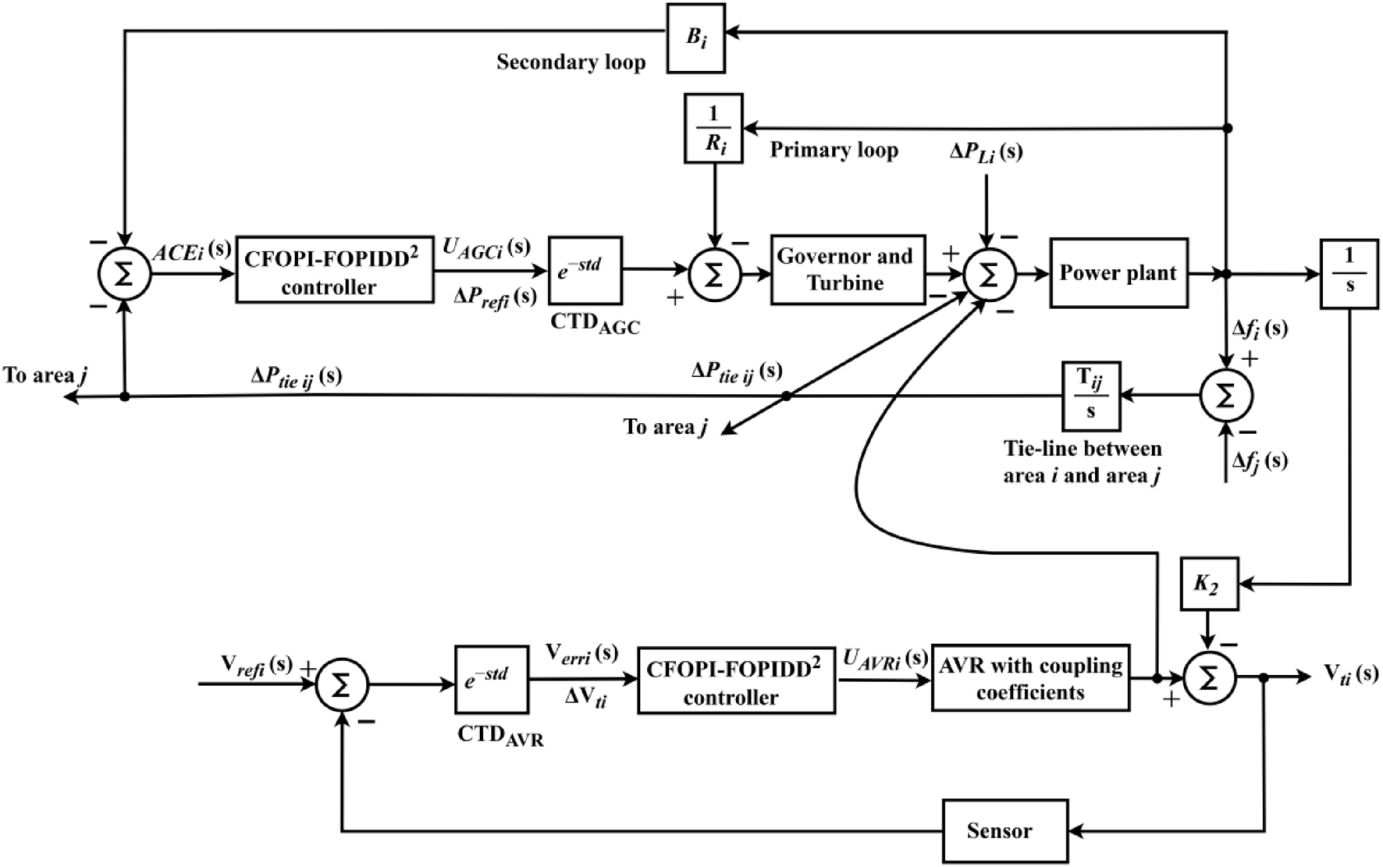
AGC-AVR loops with CTD blocks of IPS Model.

**Fig. 16:**
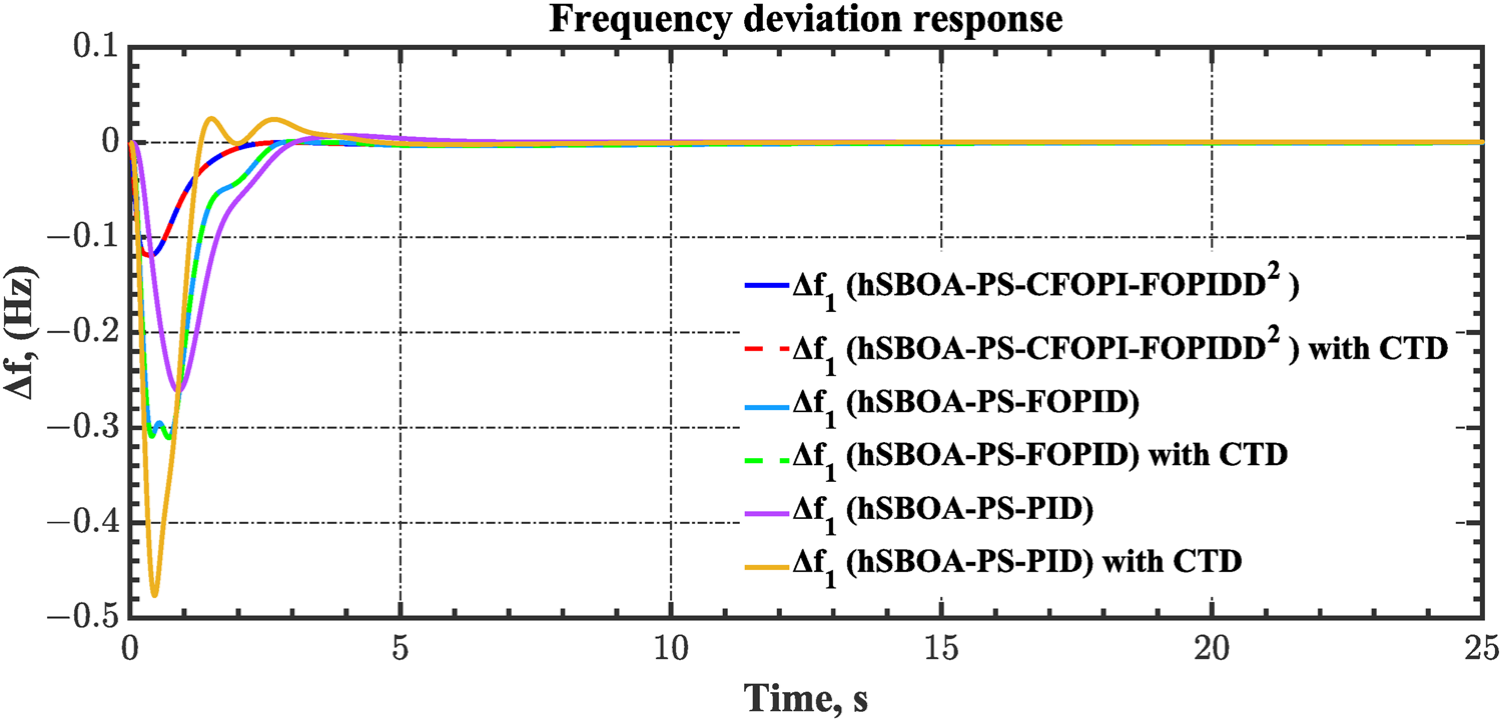
Area-1’s frequency deviation responses according to the second scenario.

**Fig. 17:**
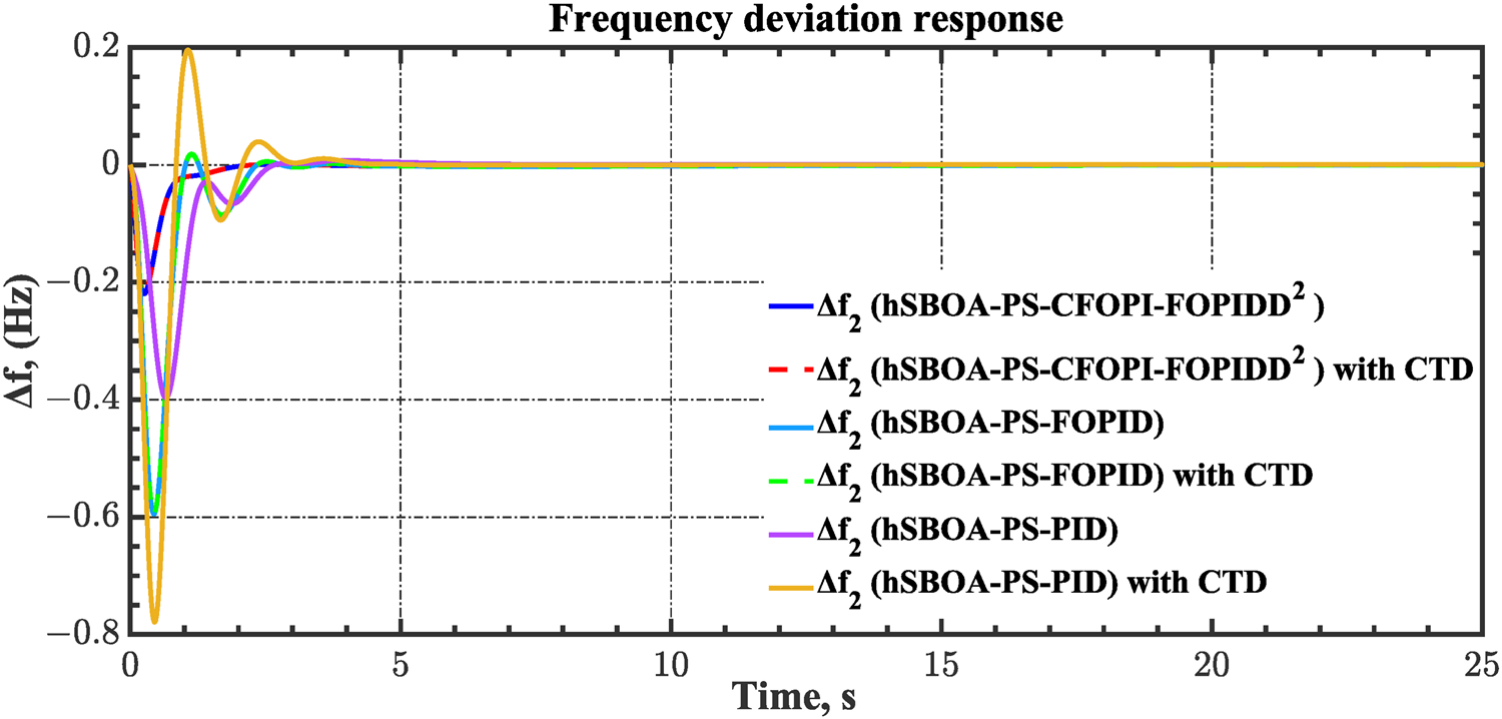
Area-2’s frequency deviation responses according to the second scenario.

**Fig. 18:**
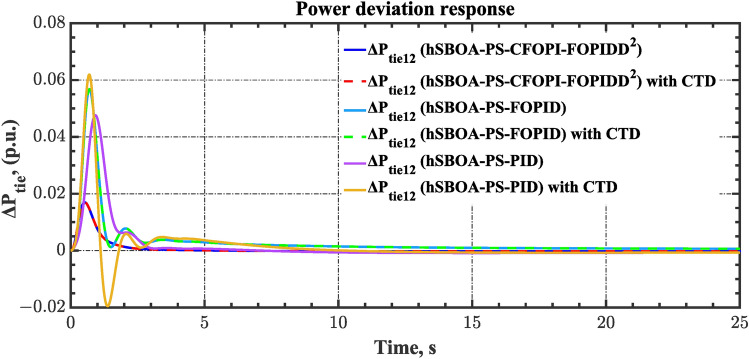
Power deviation responses according to the second scenario.

**Fig. 19:**
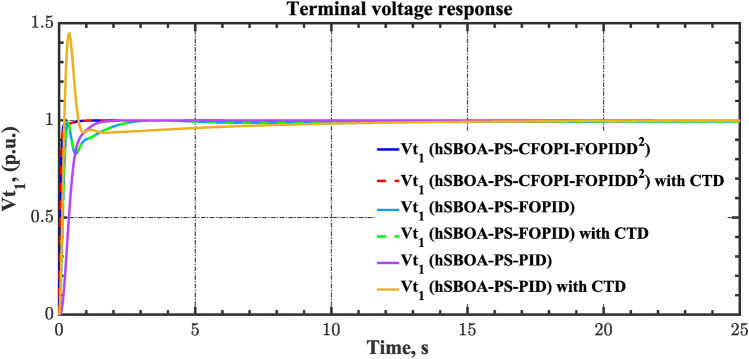
Terminal voltage responses for area-1 according to the second scenario.

**Fig. 20:**
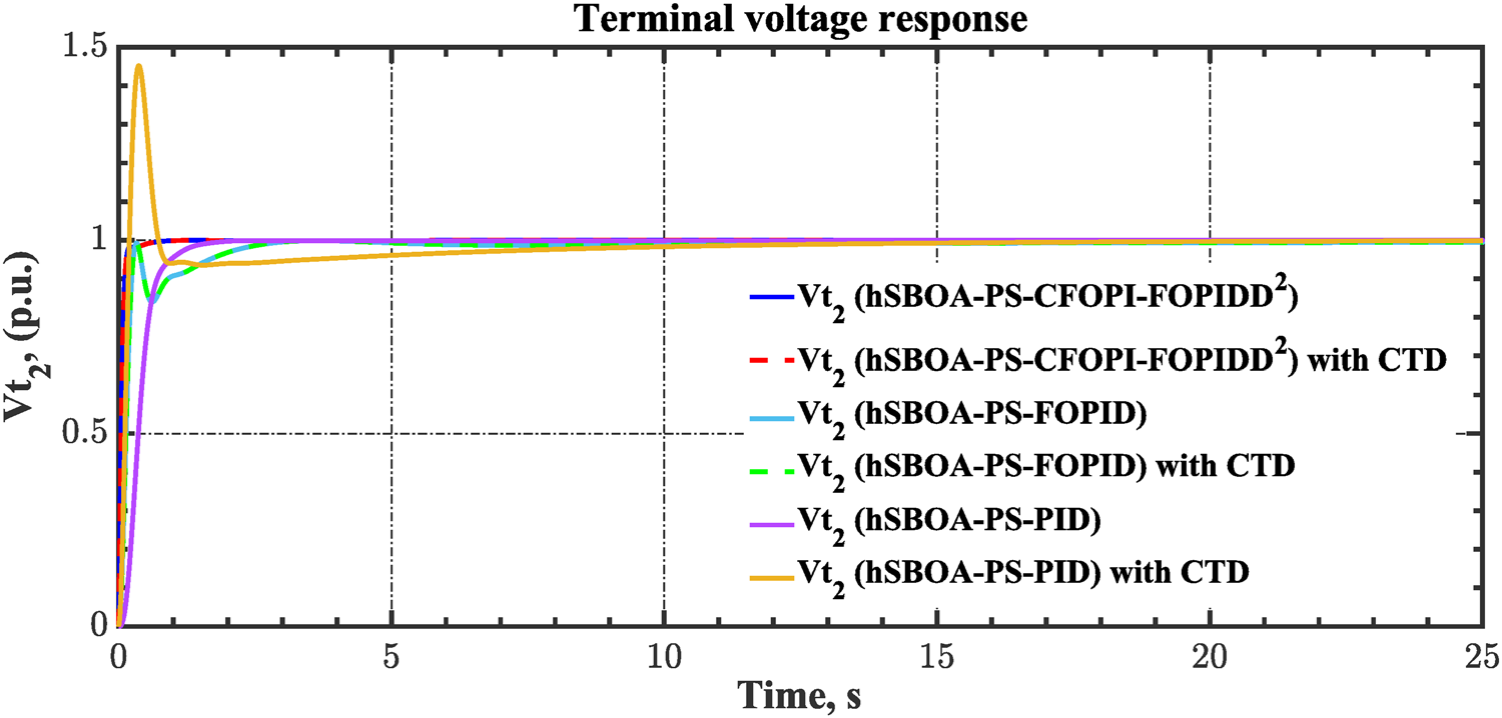
Terminal voltage responses for area-2 according to the second scenario.

Load dispatch centre (LDC) is located at a considerable distance from the power sources. The power sources capture signals from the LDC, which in turn regulate their power output. As a result, there is a CTD between the LDC and power sources^36^. So, when dynamically modeling the power system, it is essential to consider the CTD. The time-varying CTDs are further realistic and prevalent in actual networks compared to regular CTDs, as system reaction or data transmission delays are often affected by multiple factors. These factors include grid connection delays, mechanical systems, control mechanisms, and environmental conditions^48^. Here, the AGC-AVR loops are equipped with a time-varying CTD that follows the pattern depicted in Fig. 14. Figure 15 showed the AGC-AVR loops with CTD^27^. In this scenario, a 5% SLD is occurred in region two at t = 0 s. Also, the ${V}_{ref}$ is considered as 1 p.u. for both areas. Additionally, the wind and solar units’ electricity generation are inaccessible. Table 8 summarizes the ITSE values obtained via three different controllers in accounting of the effect of CTD. Figures 16, 17, 18, 19 and 20 present the system’s dynamic responses utilizing the three regulators. Table 8 and the Figs. 16, 17, 18, 19 and 20 show that when a PID controller is utilized, the CTDs have a bigger impact on the AGC-AVR loops and cannot be ignored. However, the CFOPI-FOPIDD^2^ and FOPID controllers are efficient and successfully mitigate the impact of CTDs.

**Table 8:** ITSE value under CTD influence utilizing various controllers.

Controller	ITSE					The total ITSE
	${\Delta f}_{1}$	${\Delta f}_{2}$	${\Delta P}_{tie12}$	${\Delta V}_{1}$	${\Delta V}_{2}$	
hSBOA-PS-CFOPI-FOPIDD^2^	**0.005336**	**0.005472**	**0.0001155**	**0.001301**	**0.001298**	**0.01352**
hSBOA-PS-FOPID	0.053	0.06606	0.001601	0.04021	0.03949	0.2004
hSBOA-PS-PID	0.0634	0.1115	0.001586	0.09164	0.09366	0.3618

The figures in bold are the best.

#### Scenario 3: RLD and TVDV impact

**Fig. 21:**
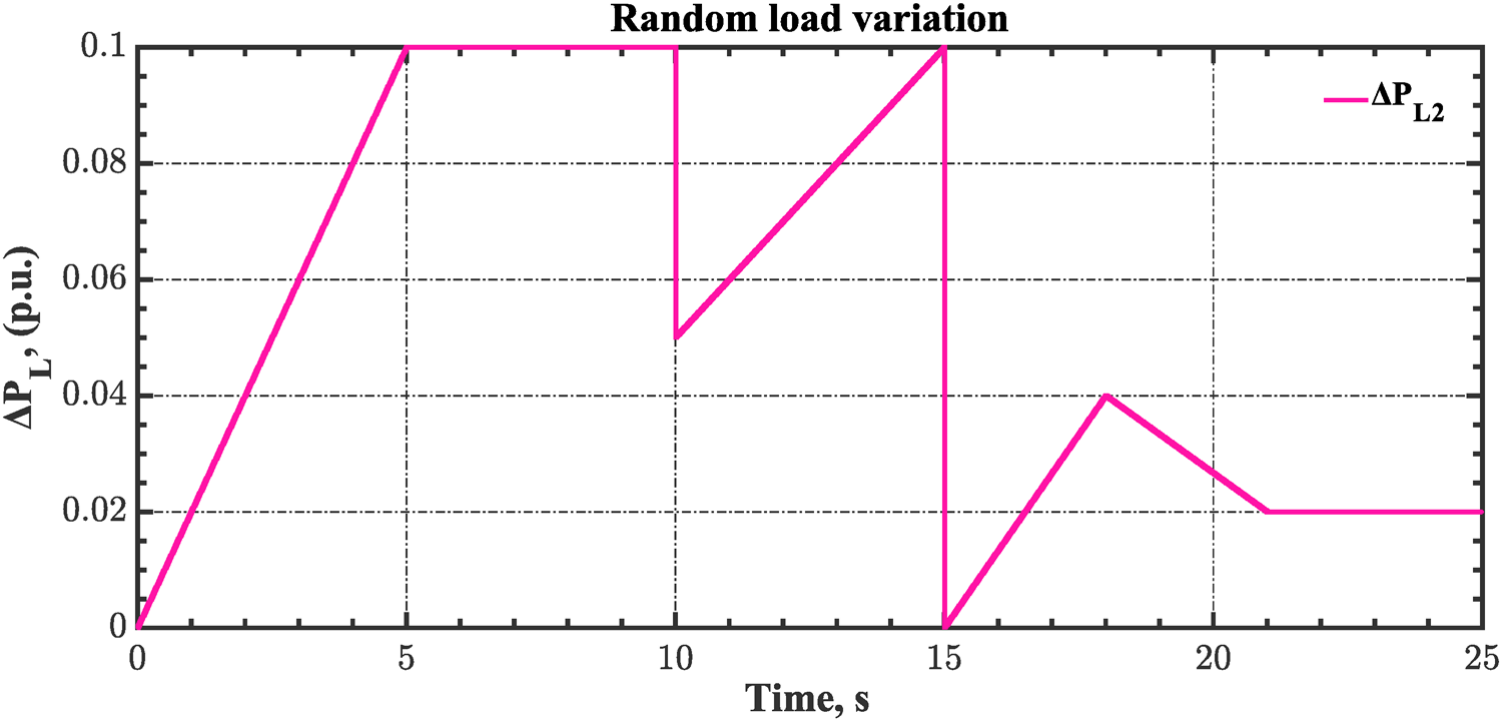
RLD pattern.

**Fig. 22:**
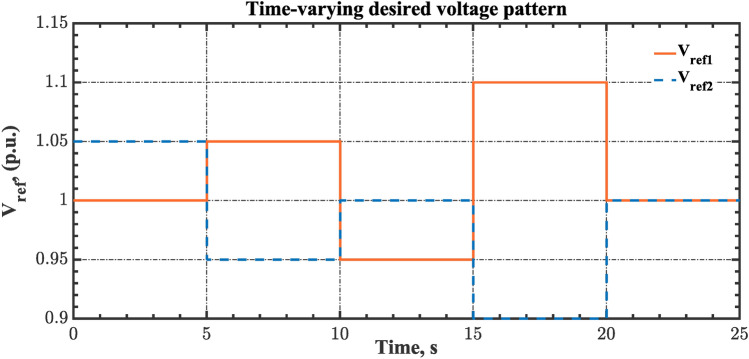
TVDV pattern.

**Fig. 23:**
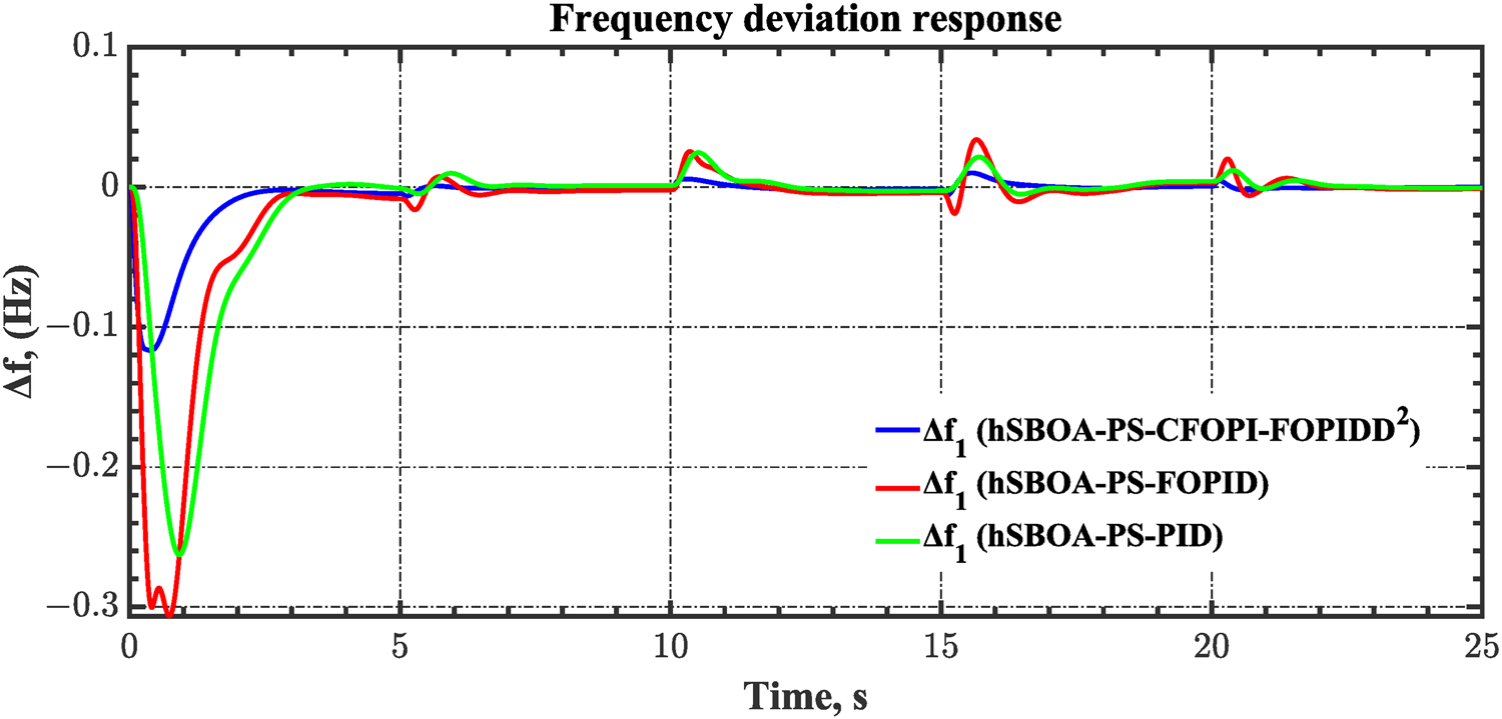
Area-1’s frequency deviation responses according to the third scenario.

**Fig. 24:**
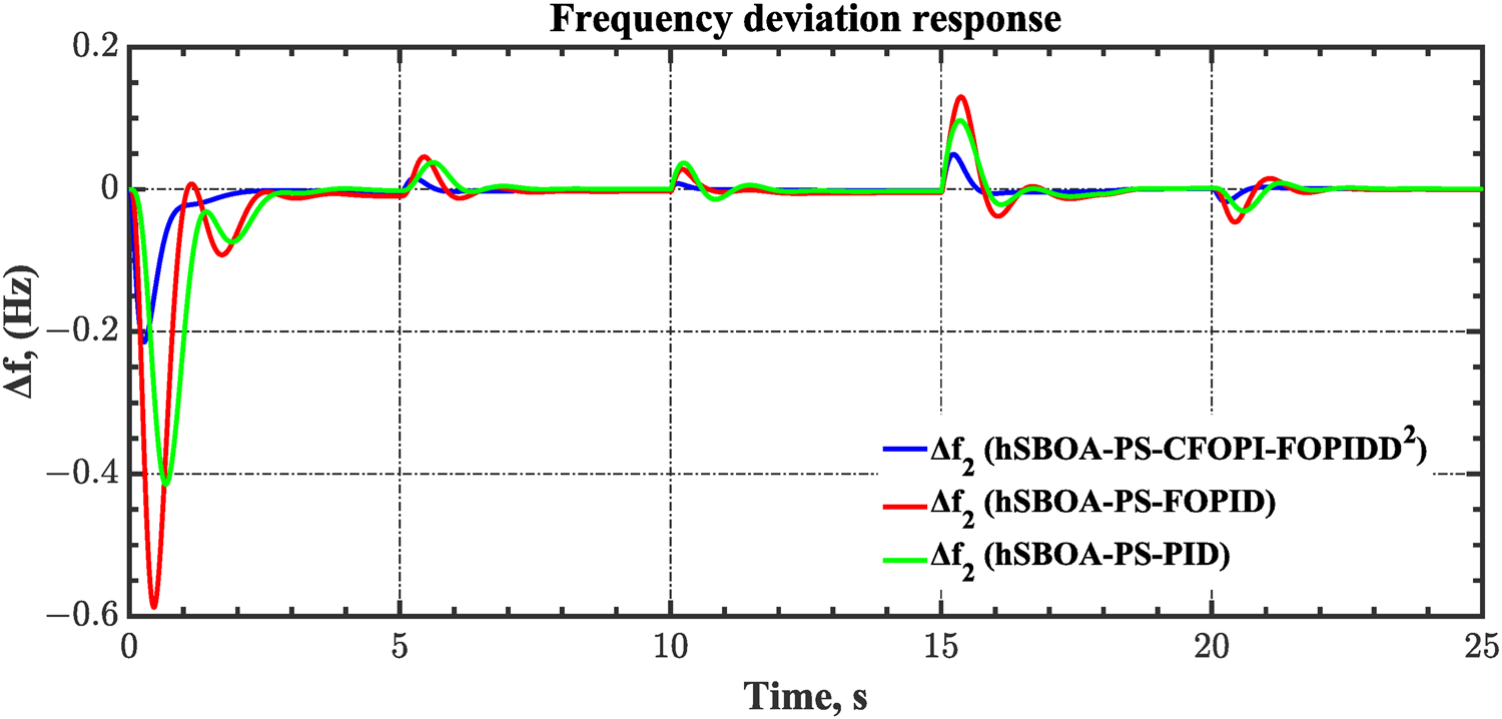
Area-2’s frequency deviation responses according to the third scenario.

**Fig. 25:**
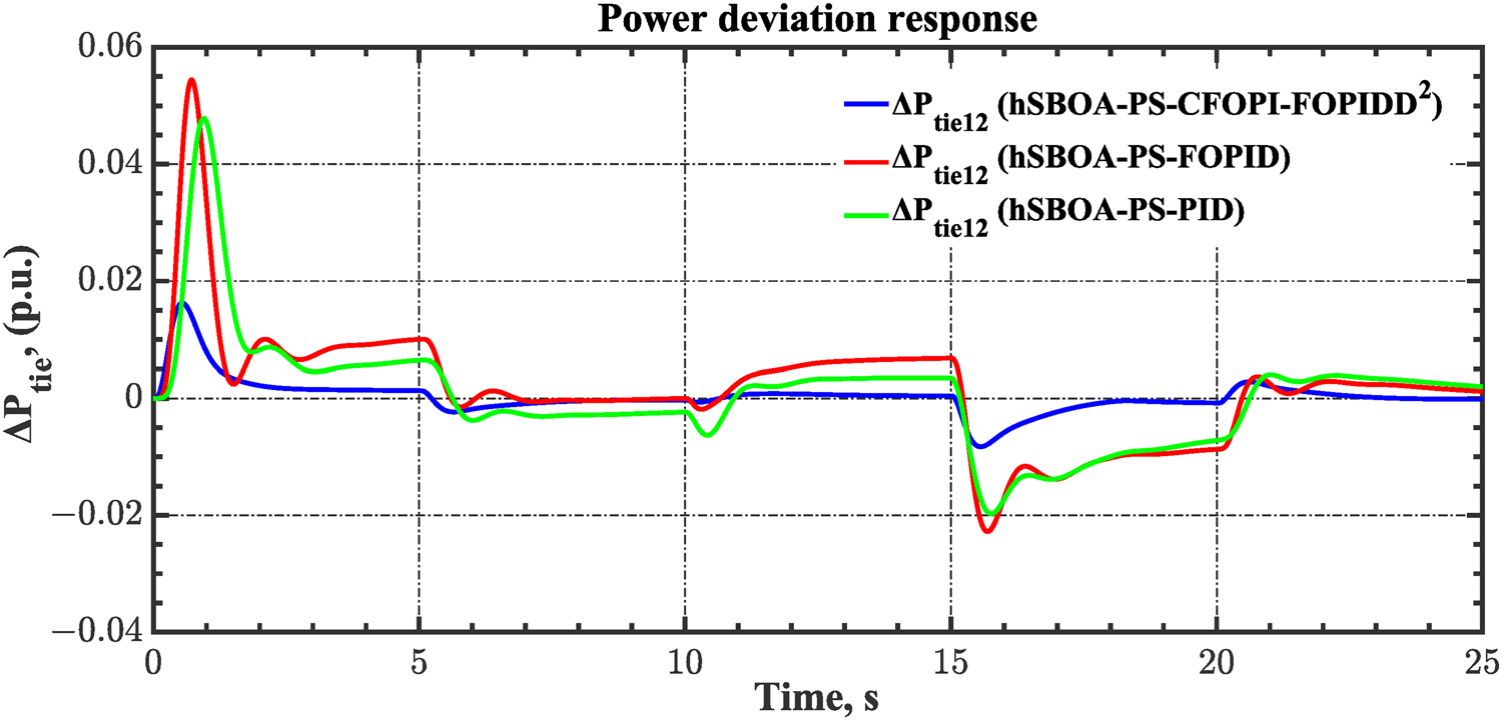
Power deviation according to the third scenario.

**Fig. 26:**
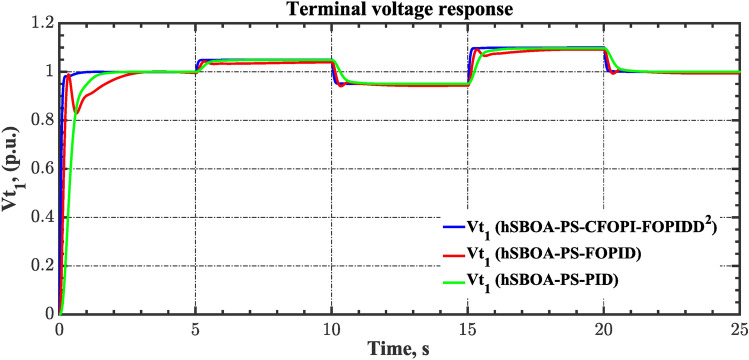
Terminal voltage responses for area-1 according to the third scenario.

**Fig. 27:**
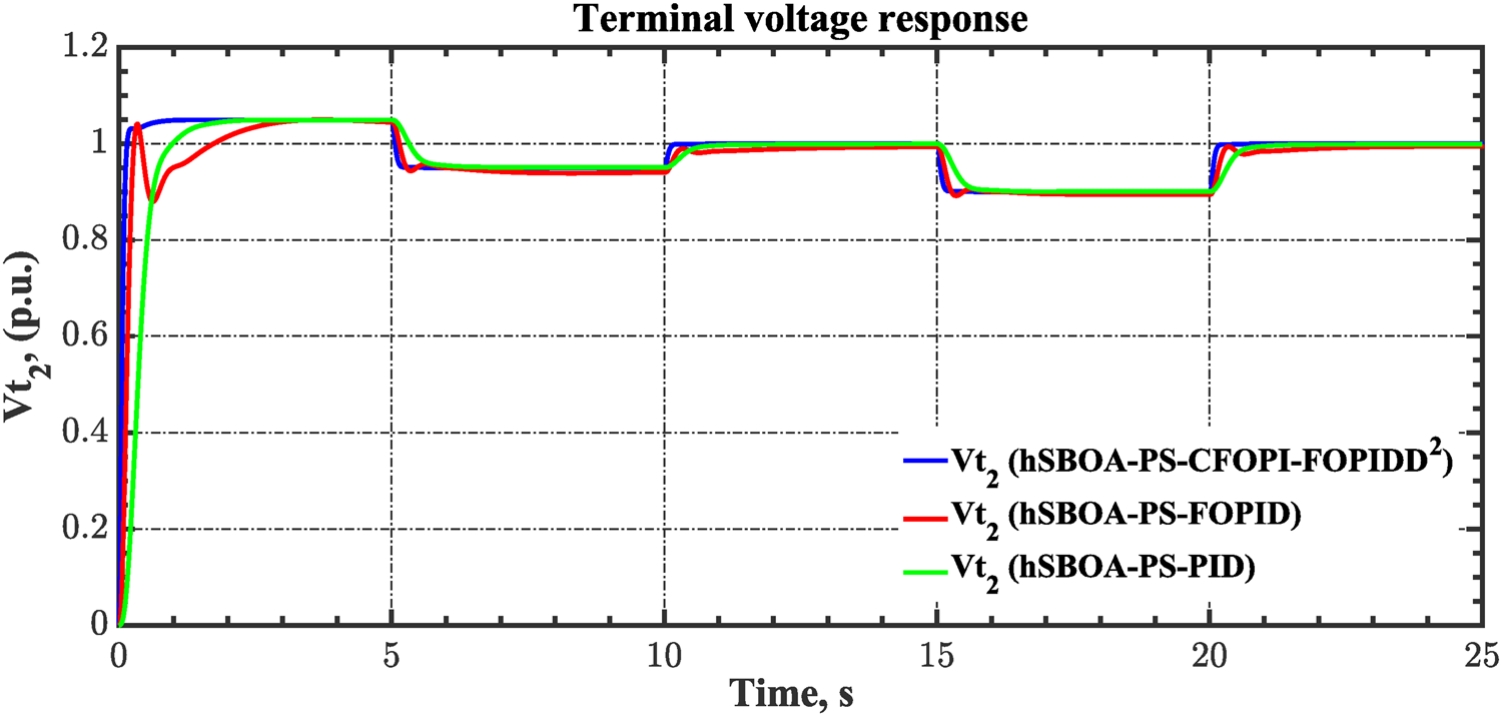
Terminal voltage responses for area-2 according to the third scenario.

In this scenario, the suggested AGC-AVR loops’ performance is evaluated using the simultaneous effects of RLD and TVDV. Figures 21 and 22 show the pattern of RLD and TVDV, respectively. Furthermore, electricity generation from wind and solar systems is inaccessible in this scenario. Table 9 presents the ITSE values achieved by the various controllers under this case. On the other hand, Figs. 23, 24, 25, 26 and 27 present the system’s dynamic responses utilizing the three regulators. Based on Table 9, the suggested controller was able to produce an ITSE value that is roughly 6.6 times less than the FOPID regulator and 7.9 times less than the PID regulator. So, that illustrates that the suggested controller outperformed other controllers in relation to voltage, frequency, and power tie. It is important to note that the superior performance of the CFOPI-FOPIDD2 controller comes at the cost of increased implementation complexity. A key drawback of this controller lies in its cascaded structure, which integrates two FO controllers. This configuration results in a high number of tuning parameters, rendering classical tuning methods impractical. Nevertheless, the advantages offered by the CFOPI-FOPIDD2 controller outweigh these limitations, making it a compelling choice for robust AGC-AVR loops.

**Table 9:** ITSE value under scenario 3 influence utilizing various controllers.

Controller	ITSE					The Total ITSE
	${\Delta f}_{1}$	${\Delta f}_{2}$	${\Delta P}_{tie12}$	${\Delta V}_{1}$	${\Delta V}_{2}$	
hSBOA-PS-CFOPI-FOPIDD^2^	**0.006814**	**0.01898**	**0.001243**	**0.02931**	**0.01989**	**0.07624**
hSBOA-PS-FOPID	0.06855	0.1918	0.01762	0.1263	0.09631	0.5006
hSBOA-PS-PID	0.06571	0.1414	0.01568	0.2149	0.1645	0.6022

The figures in bold are the best.

As can be observed from Figs. 23, 24, 25, 26 and 27, the CFOPI-FOPIDD^2^ controller took less time to return the responses to the desired values than the FOPID and PID controllers. Furthermore, utilizing the CFOPI-FOPIDD^2^ controller resulted in smaller peak amplitudes of the deviations. This indicates that the suggested method improved the stability and performance of AGC-AVR loops.

#### Scenario 4: Solar irradiance variation impact

**Fig. 28:**
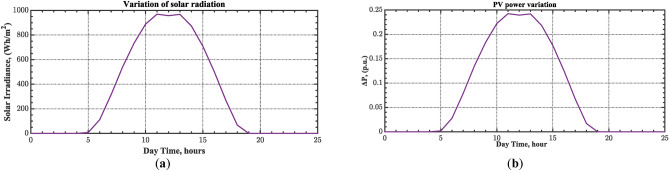
Actual solar profile (**a**) Solar radiation, (**b**) PV power.

**Fig. 29:**
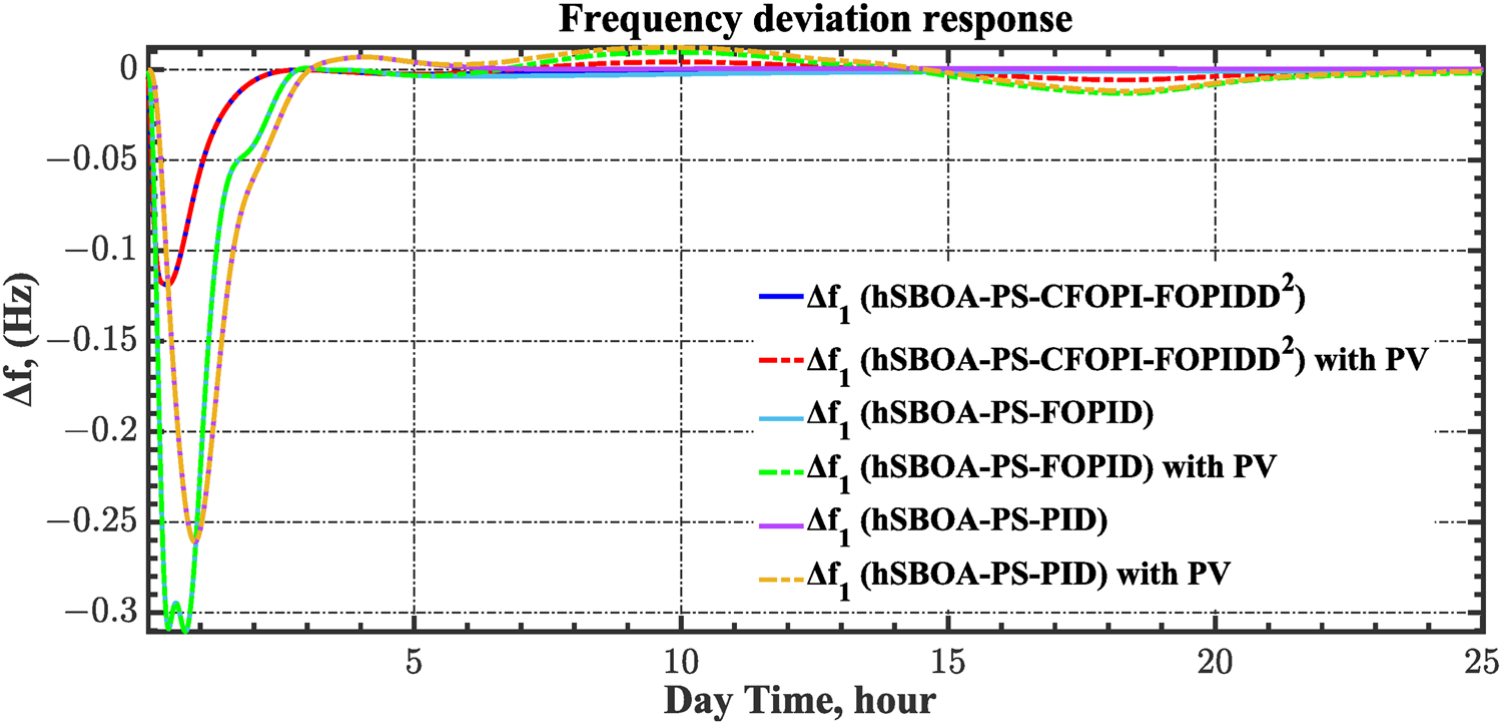
Area-1’s frequency deviation responses according to the fourth scenario.

**Fig. 30:**
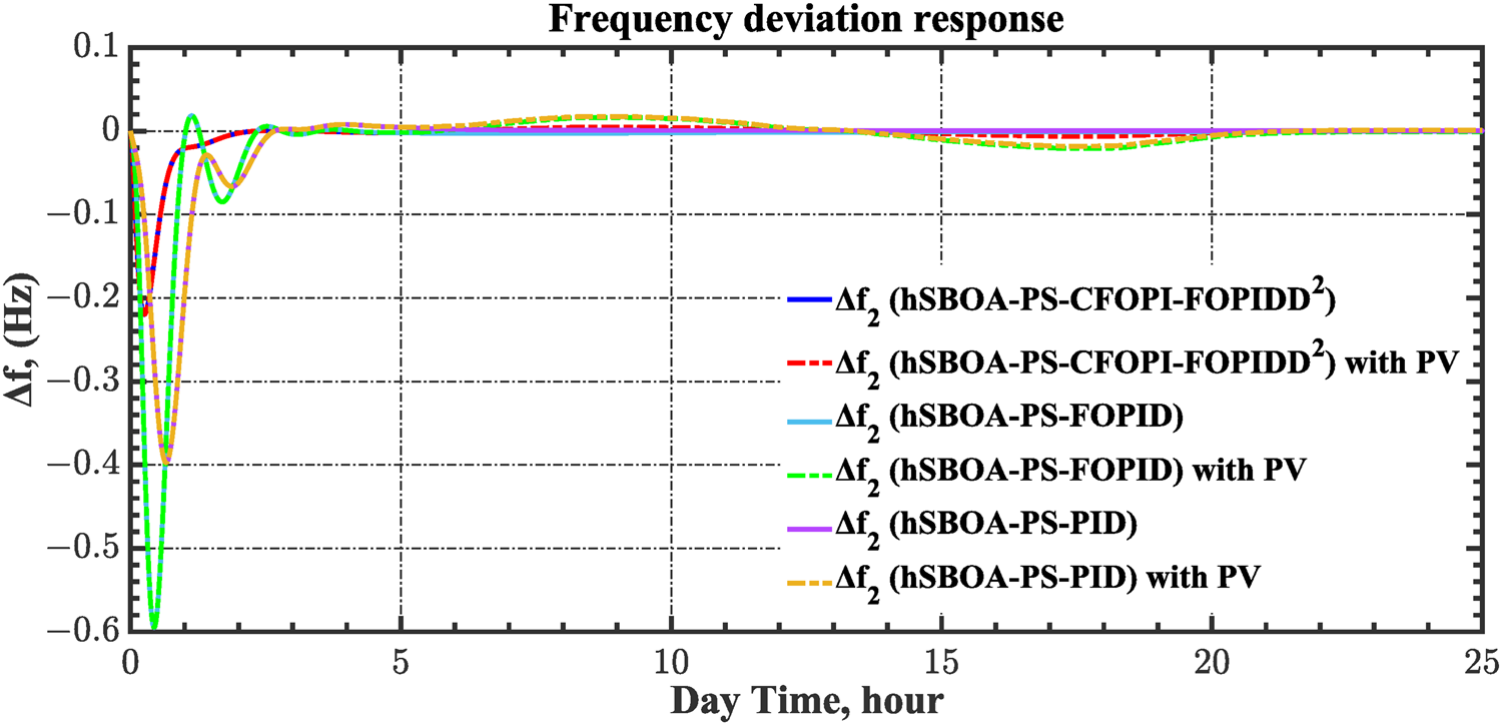
Area-2’s frequency deviation responses according to the fourth scenario.

**Fig. 31:**
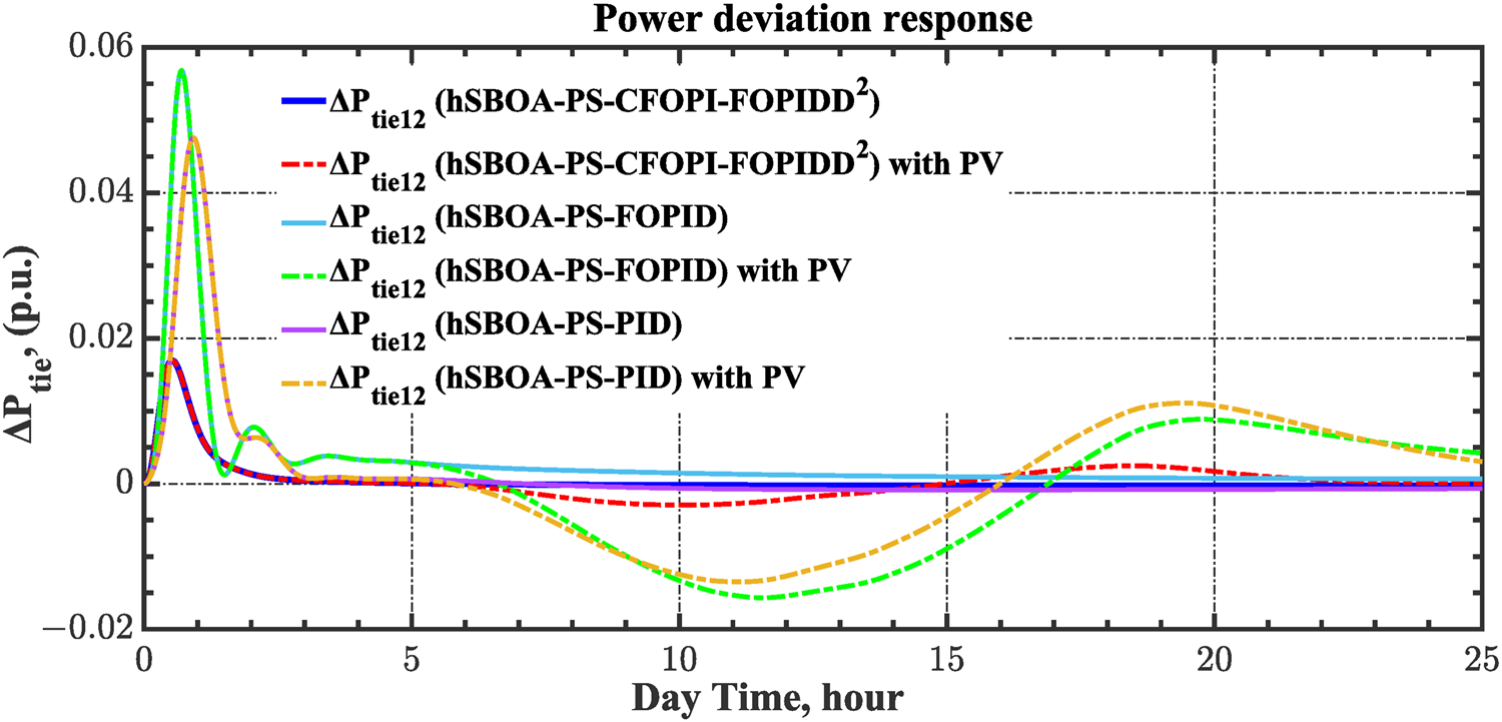
Power deviation responses according to the fourth scenario.

**Fig. 32:**
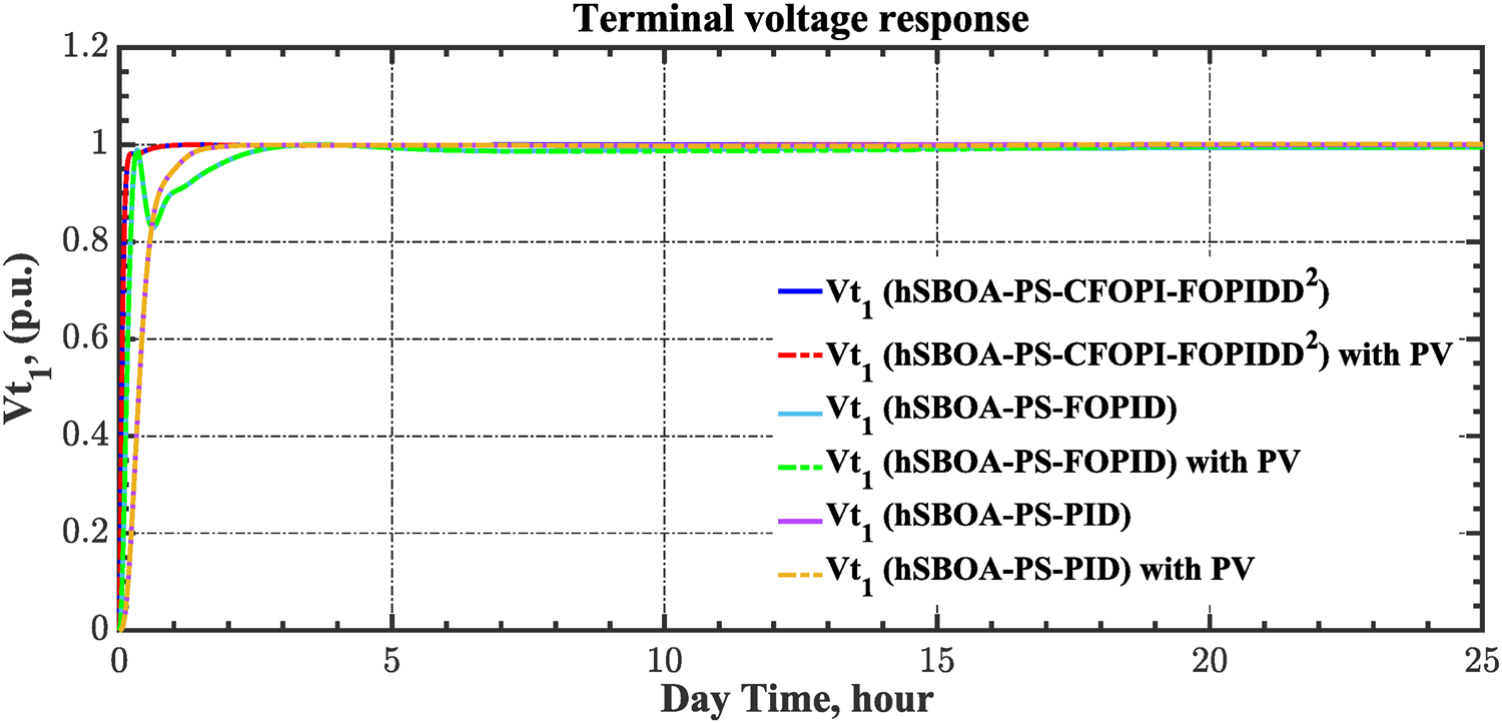
Terminal voltage responses for area-1 according to the fourth scenario.

**Fig. 33:**
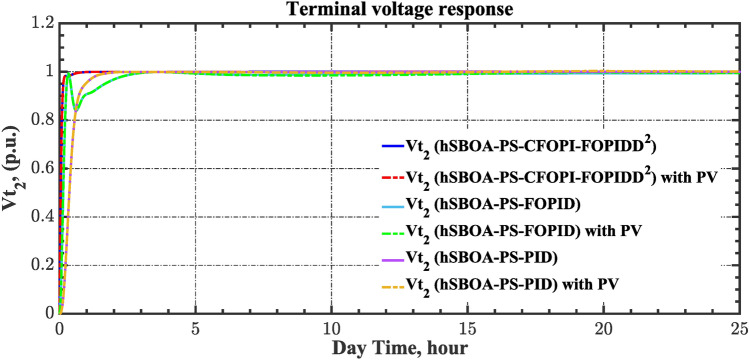
Terminal voltage responses for area-2 according to the fourth scenario.

The case four considers the influence of the random solar irradiation in the performance of the AGC-AVR loops. Figure 28 depicts the PV profile that is applied area 2 which is a real power generation profile of the PV system obtained through NASA/POWER CERES/MERRA2 Native Resolution Hourly Data in Yanbu, KSA (with Latitude 24.0895 and Longitude 38.0618) on 1 July 2024^48,49^. Also, a 5% SLD is occurred in region two at t = 0 s and the ${V}_{ref}$ is considered as 1 p.u. for both areas. Furthermore, wind power generation is inaccessible in this case. Table 10 illustrates the ITSE values obtained by the different controllers in this situation. Figures 29, 30, 31, 32 and 33 depict the system’s dynamic responses of the AGC-AVR loops. Table 10 shows that in comparison to the FOPID controller and the PID controller, the hSBOA-PS-CFOPI-FOPIDD^2^ controller achieved an ITSE value that was about 13.94 and 14.52 times lower, respectively. According to Figs. 29, 30, 31, 32 and 33, the actual solar profile generates random disturbances in AGC responses. However, when the hSBOA-PS-CFOPI-FOPIDD^2^ controller is used, the AGC performance is more stable than others. Thus, the PV system presence in the IPS significantly affected the performance of hSBOA-PS-FOPID and hSBOA-PS-PID controllers.

**Table 10:** ITSE value under scenario 4 influence utilizing various controllers.

Controller	ITSE					The total ITSE
	${\Delta f}_{1}$	${\Delta f}_{2}$	${\Delta P}_{tie12}$	${\Delta V}_{1}$	${\Delta V}_{2}$	
hSBOA-PS-CFOPI-FOPIDD^2^	**0.00793**	**0.008422**	**0.0008363**	**0.001308**	**0.001306**	**0.0198**
hSBOA-PS-FOPID	0.06695	0.1004	0.02324	0.04271	0.04265	0.276
hSBOA-PS-PID	0.06978	0.08606	0.02156	0.05499	0.05505	0.2875

The figures in bold are the best.

#### Scenario 5: Wind speed variation impact

**Fig. 34:**
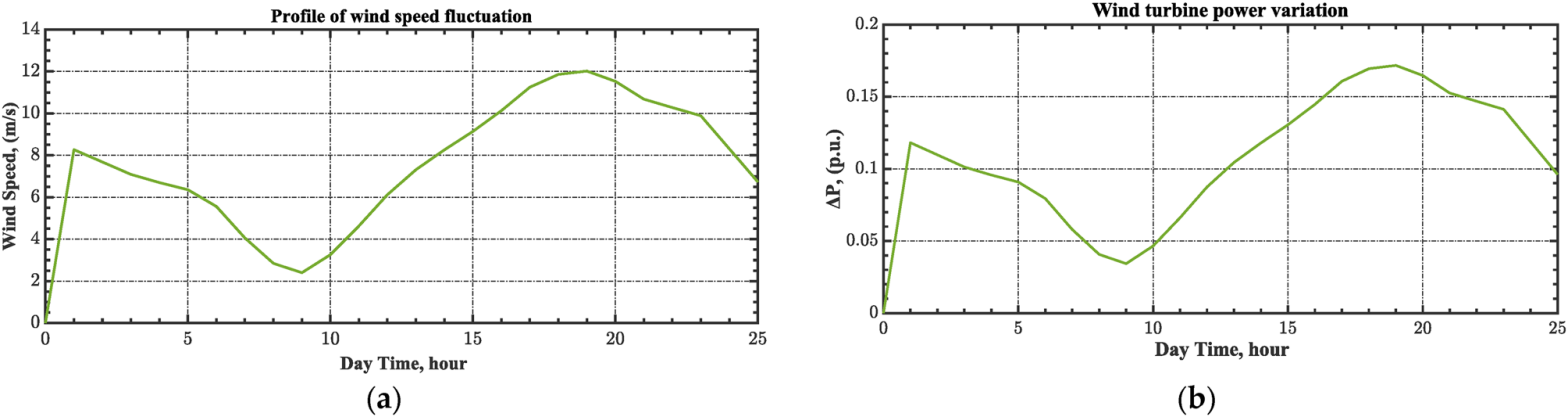
Actual wind profile (**a**) Wind speed, (**b**) Wind turbine power.

**Fig. 35:**
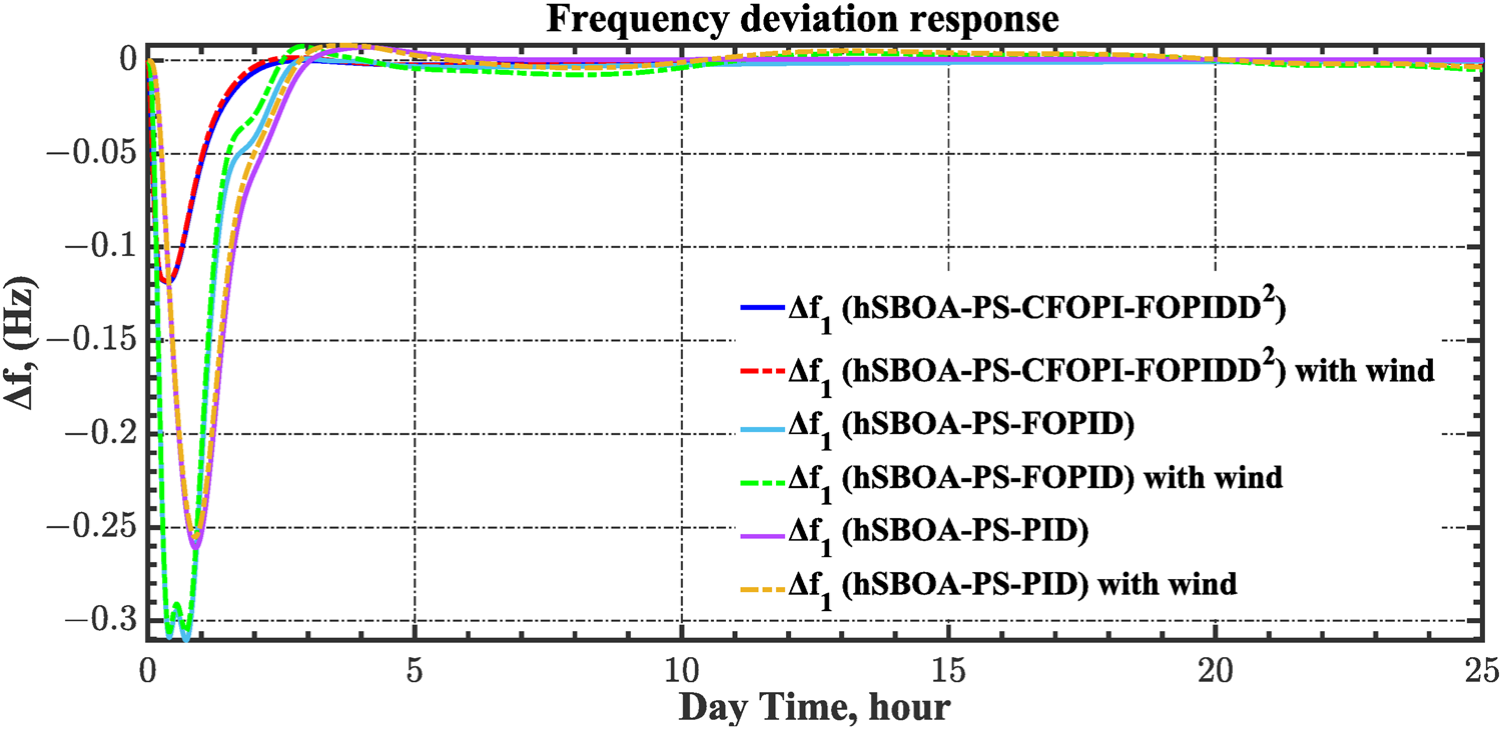
Area-1’s frequency deviation responses according to the fifth scenario.

**Fig. 36:**
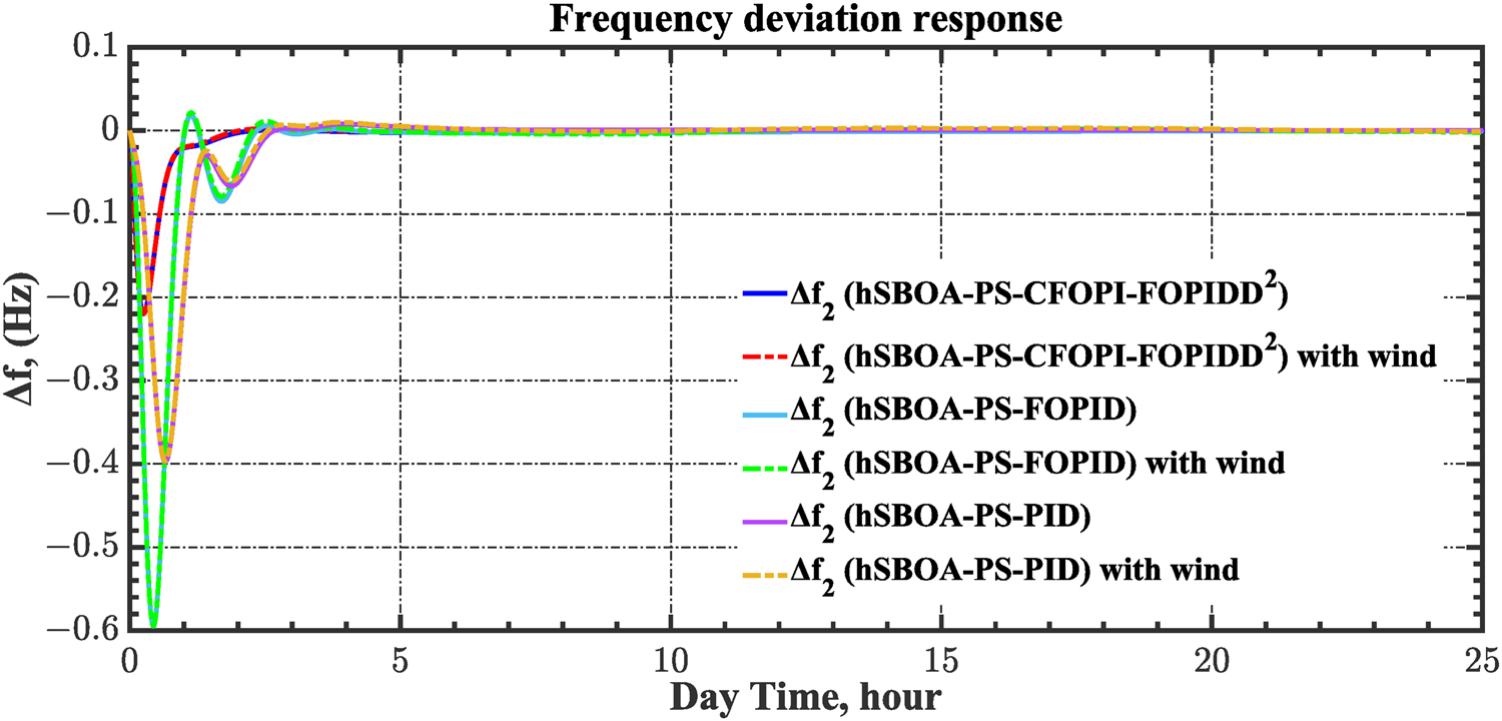
Area-2’s frequency deviation responses according to the fifth scenario.

**Fig. 37:**
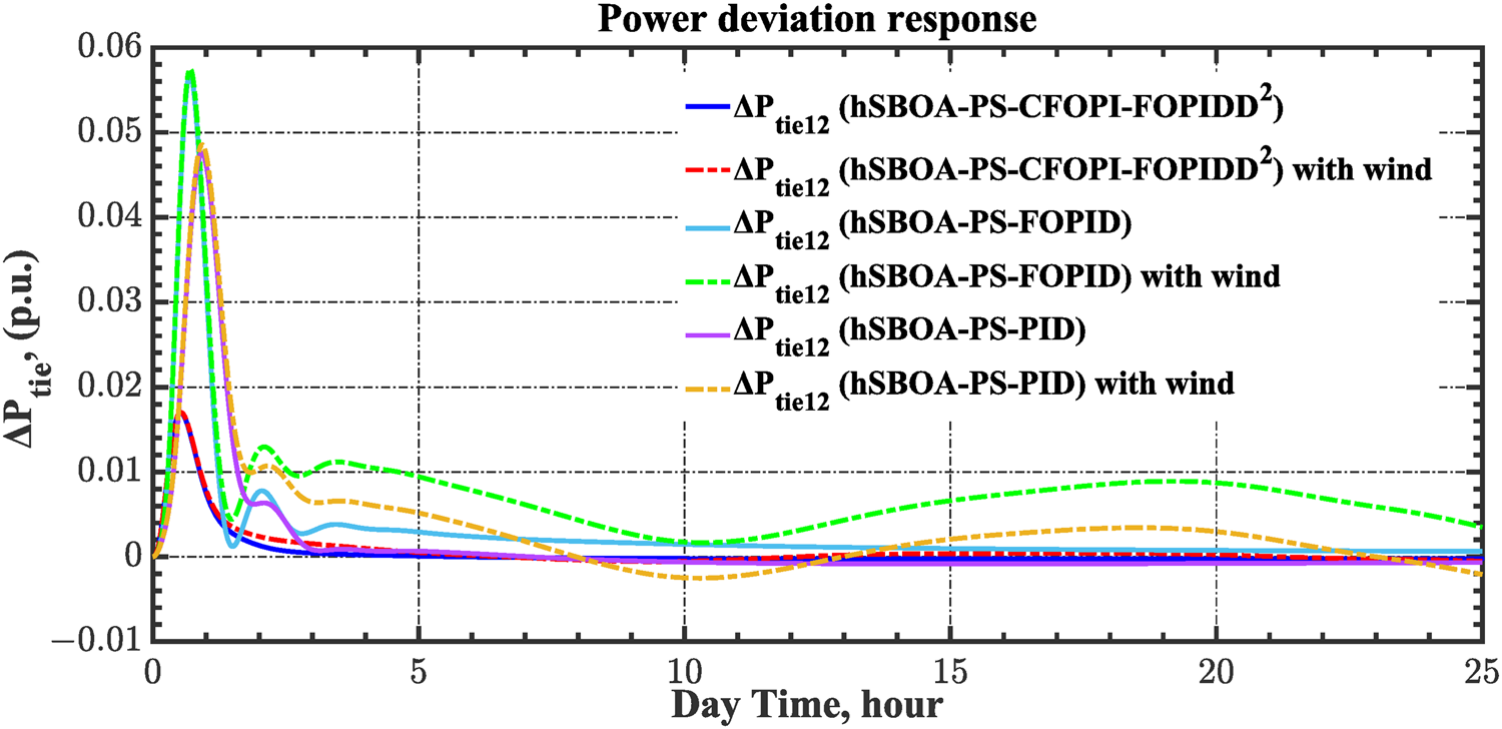
Power deviation responses according to the fifth scenario.

**Fig. 38:**
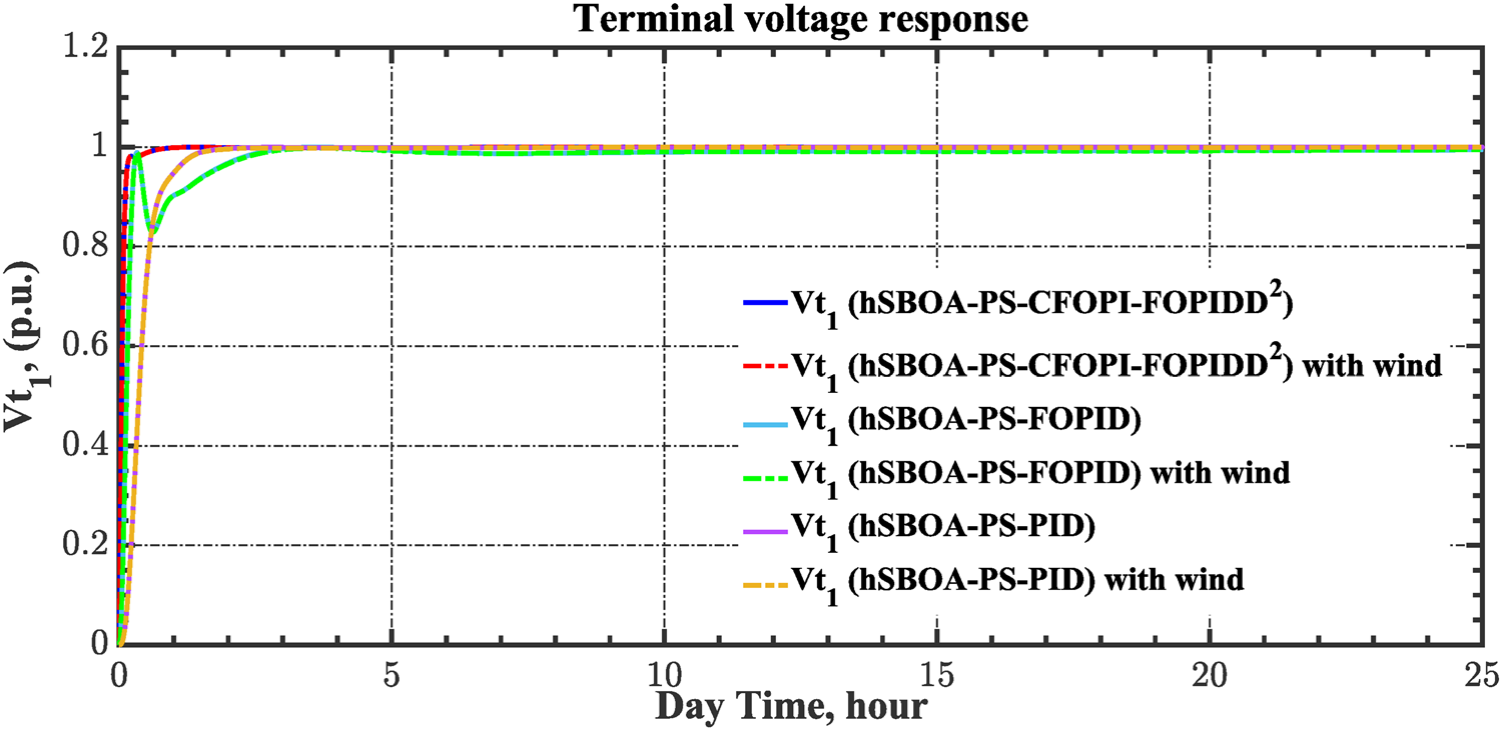
Terminal voltage responses for area-1 according to the fifth scenario.

**Fig. 39:**
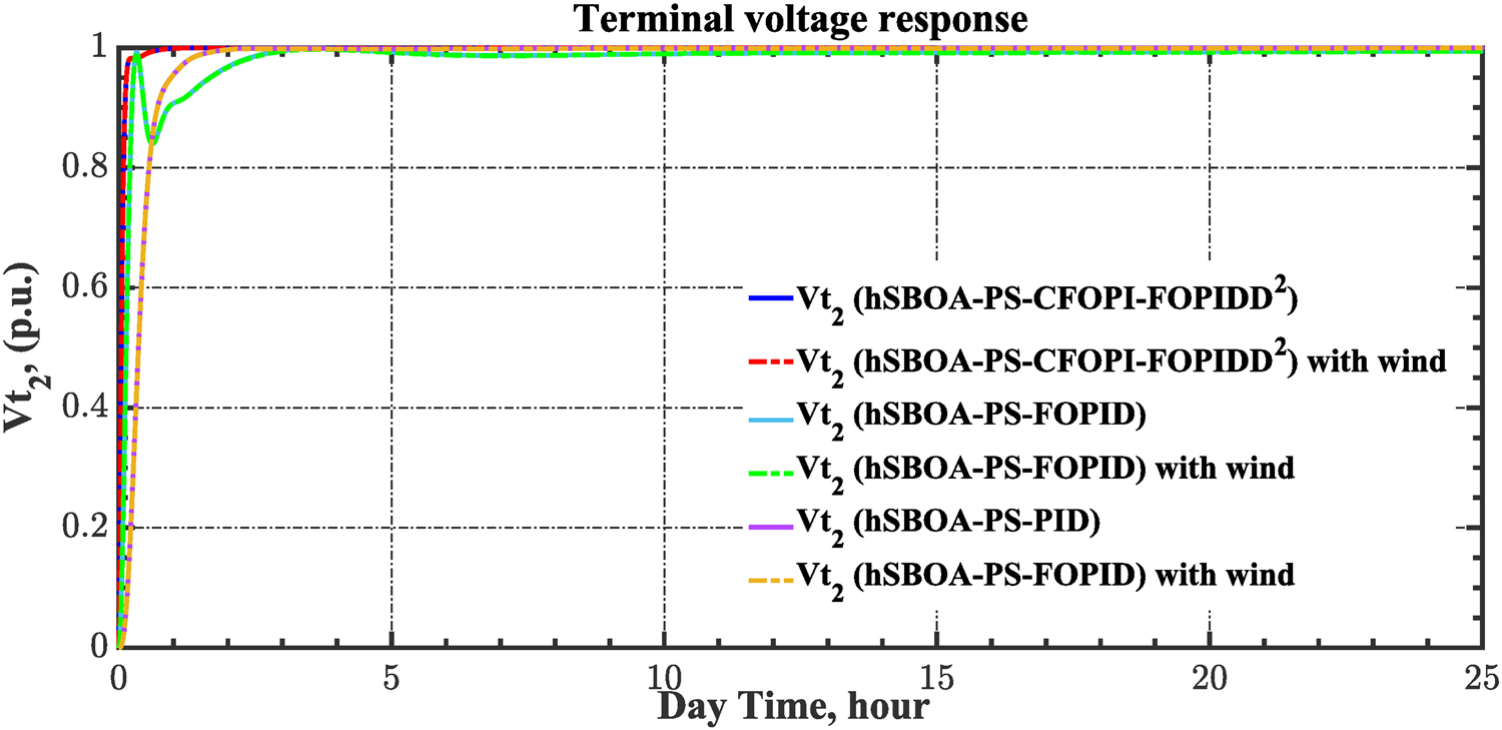
Terminal voltage responses for area-2 according to the fifth scenario.

The current case examines the influence of fluctuations in wind speed on AGC-AVR loop efficiency. Figure 34 depicts the wind profile that is applied area 1 which is a real power generation profile of the wind system obtained through NASA/POWER CERES/MERRA2 Native Resolution Hourly Data in Yanbu, KSA (with Latitude 24.0895 and Longitude 38.0618) on 1 July 2024^48,49^. Also, a 5% SLD is occurred in region two at t = 0 s and the ${V}_{ref}$ is considered as 1 p.u. for both areas. Furthermore, PV power generation is inaccessible in this case. Table 11 illustrates the ITSE values obtained by the different controllers in this case. Figures 35, 36, 37, 38 and 39 depict the system’s dynamic responses of the AGC-AVR loops. Table 11 indicates that the hSBOA-PS-CFOPI-FOPIDD^2^ controller attained an ITSE value of 16.26 and 16.37 times lower than the FOPID and PID controllers. The actual wind profile causes oscillations in AGC responses, as indicated by Figs. 35, 36, 37, 38 and 39. Nevertheless, the impact of wind is minimal when utilizing the hSBOA-PS-CFOPI-FOPIDD^2^ controller. Consequently, the presence of wind system in the IPS markedly influenced the efficacy of the hSBOA-PS-FOPID and hSBOA-PS-PID controllers.

**Table 11:** ITSE value under scenario 5 influence utilizing various controllers.

Controller	ITSE					The Total ITSE
	${\Delta f}_{1}$	${\Delta f}_{2}$	${\Delta P}_{tie12}$	${\Delta V}_{1}$	${\Delta V}_{2}$	
hSBOA-PS-CFOPI-FOPIDD^2^	**0.0052**	**0.00549**	**0.0001566**	**0.0013**	**0.001297**	**0.01344**
hSBOA-PS-FOPID	0.05182	0.06549	0.01522	0.04367	0.04228	0.2185
hSBOA-PS-PID	0.05249	0.05635	0.003395	0.05428	0.0535	0.22

The figures in bold are the best.

#### Scenario 6: Consideration of all RESs as well as RLD and TVDV

**Fig. 40:**
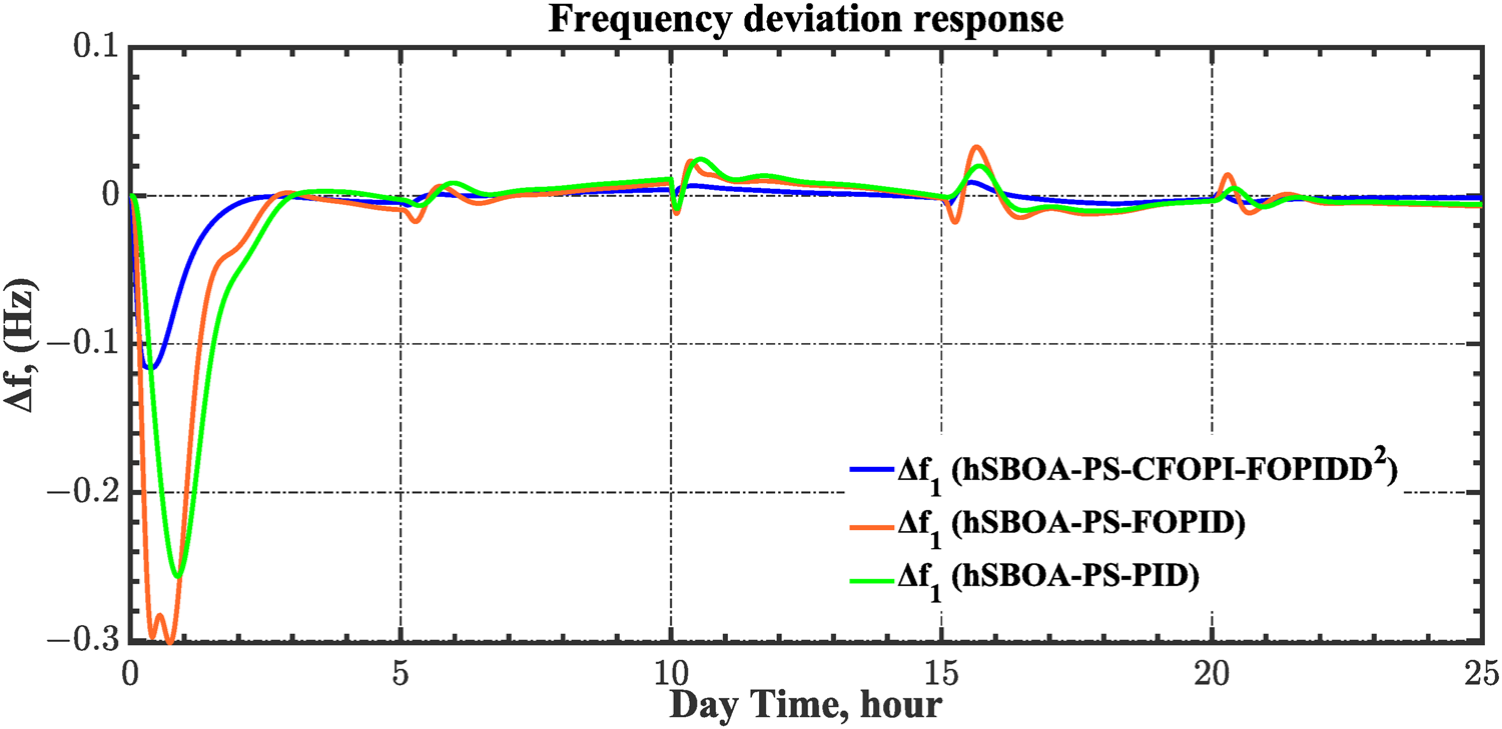
Area-1’s frequency deviation responses according to the sixth scenario.

**Fig. 41:**
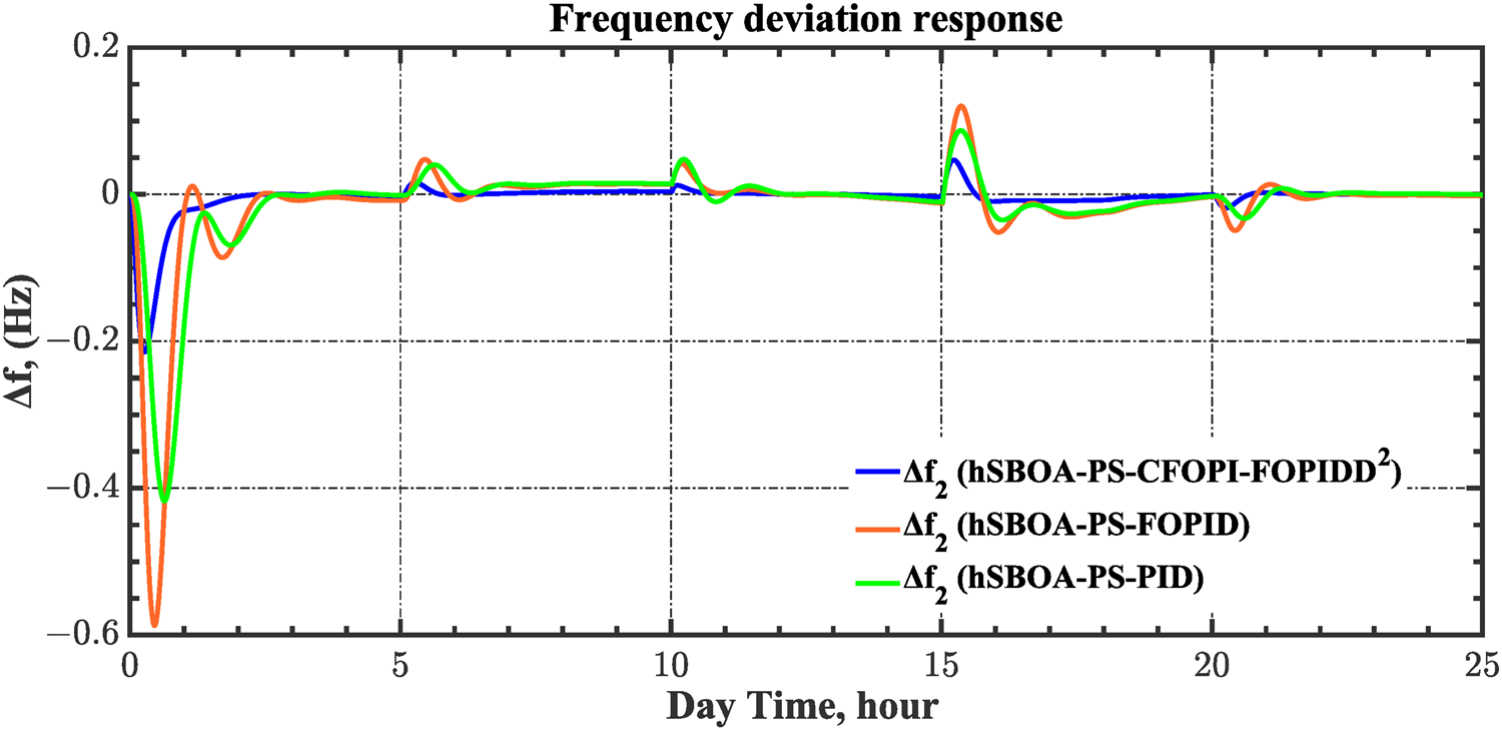
Area-2’s frequency deviation responses according to the sixth scenario.

**Fig. 42:**
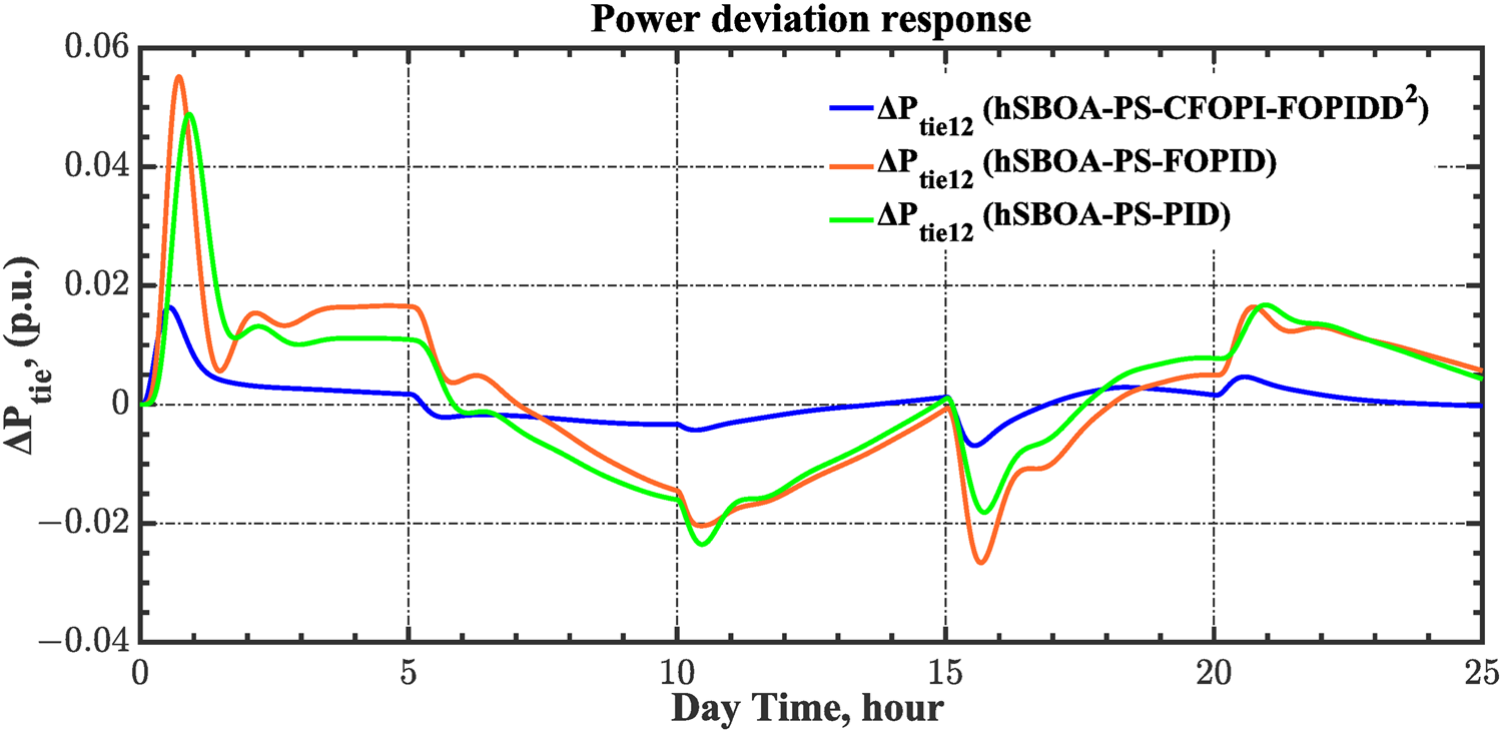
Power deviation responses according to the sixth scenario.

**Fig. 43:**
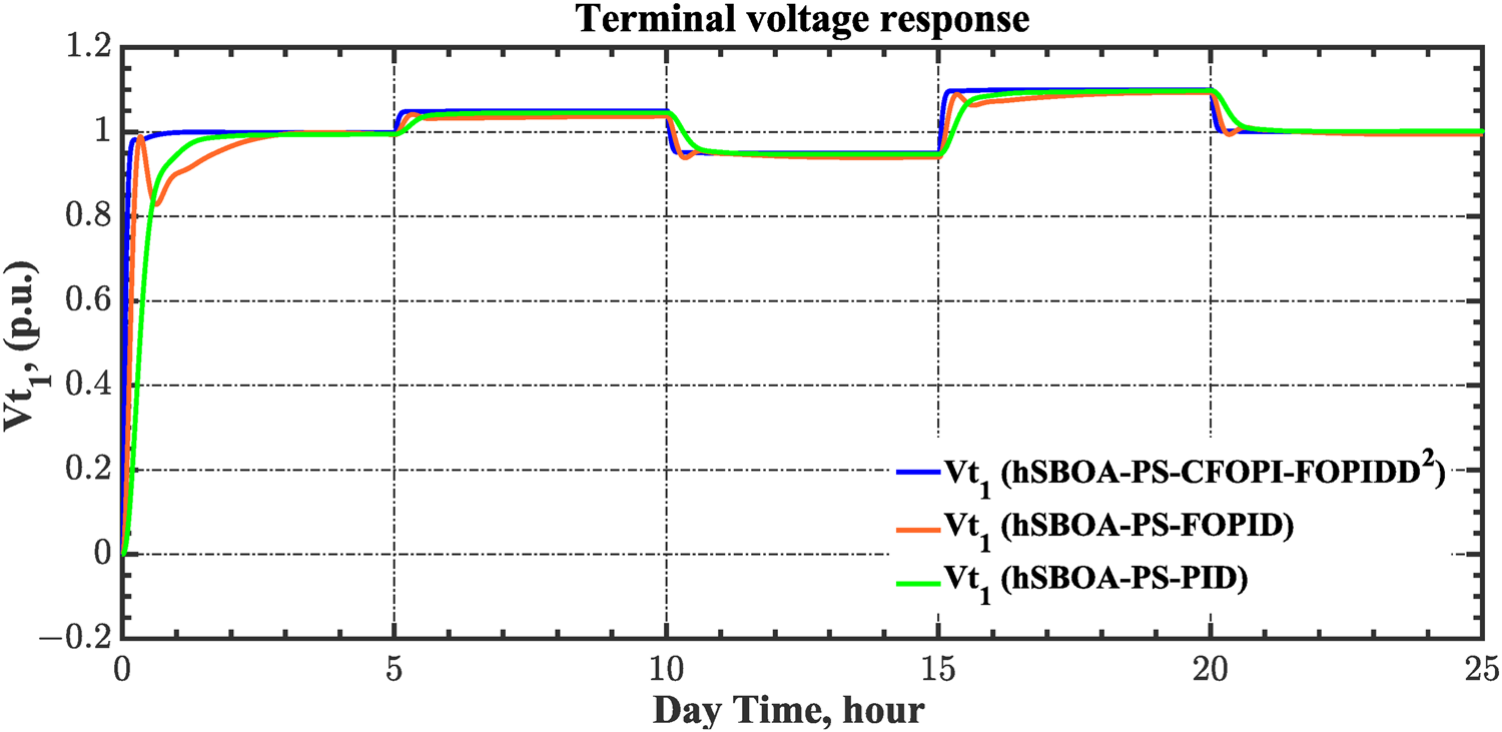
Terminal voltage responses for area-1 according to the sixth scenario.

**Fig. 44:**
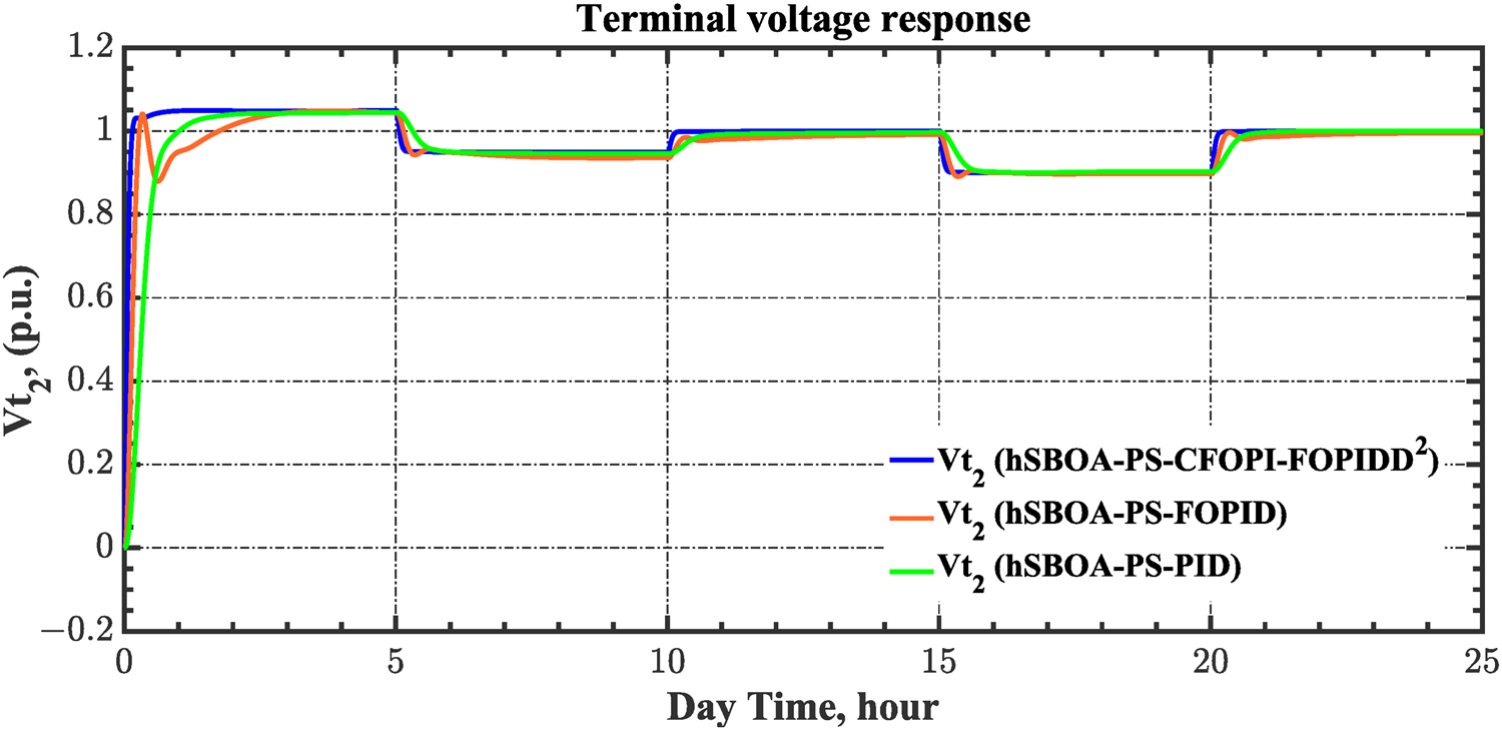
Terminal voltage responses for area-2 according to the sixth scenario.

Under the present instance, area 1 of the power system is affected by variations in wind speed, as previously depicted in Fig. 34, and experiences a 6% SLD at t = 10 h. Meanwhile, area 2 is influenced by variations in solar irradiance, shown earlier in Fig. 28, as well as by the RLD pattern illustrated in Fig. 21. Additionally, both areas are subjected to TVDVs, as described earlier in Fig. 22. This scenario aims to show the effectiveness of the suggested controller design compared to the current controllers in preserving system resilience in difficult circumstances. The outcomes are presented in Table 12 and Figs. 40, 41, 42, 43 and 44. Table 12 demonstrates that the hSBOA-PS-CFOPI-FOPIDD^2^ controller achieved an ITSE value that is 6.95 times less than the FOPID regulator and 7.79 times less than the PID regulator.

**Table 12:** ITSE value under scenario 6 influence utilizing various controllers.

Controller	ITSE					The total ITSE
	${\Delta f}_{1}$	${\Delta f}_{2}$	${\Delta P}_{tie12}$	${\Delta V}_{1}$	${\Delta V}_{2}$	
hSBOA-PS-CFOPI-FOPIDD^2^	**0.008089**	**0.02104**	**0.001878**	**0.02939**	**0.01987**	**0.08027**
hSBOA-PS-FOPID	0.07147	0.2145	0.03957	0.1339	0.09813	0.5576
hSBOA-PS-PID	0.06679	0.1576	0.03573	0.2131	0.1517	0.625

The figures in bold are the best.

The actual RESs as well as the RLD profiles introduce oscillations in the AGC responses, as shown in Figs. 40, 41 and 42. However, the impact is significantly reduced when the hSBOA-PS-CFOPI-FOPIDD^2^ controller is employed. Consequently, the incorporation of RESs units into IPS notably affects the performance of the hSBOA-PS-FOPID and hSBOA-PS-PID controllers. Furthermore, the use of the hSBOA-PS-CFOPI-FOPIDD^2^ controller led to fast response when TVDVs were applied, as illustrated in Figs. 43 and 44. This demonstrates that the proposed hSBOA-PS-CFOPI-FOPIDD^2^ enhanced the reliability and performance of the AGC-AVR loops in the difficult circumstances.

### Scenario 7: Consideration of AVR system as DISO

**Fig. 45:**
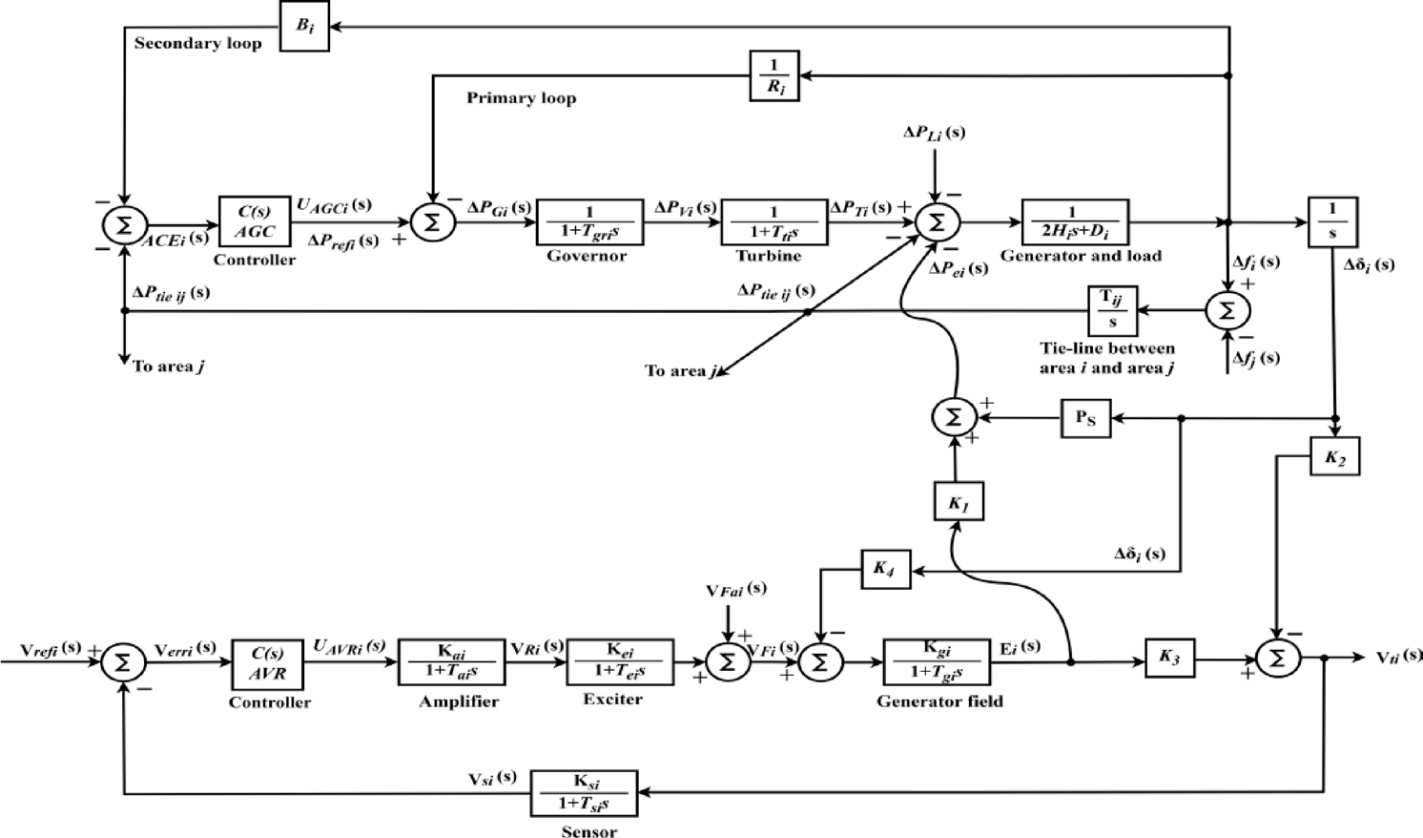
AGC-AVR_DISO_ loops of IPS Model.

**Fig. 46:**
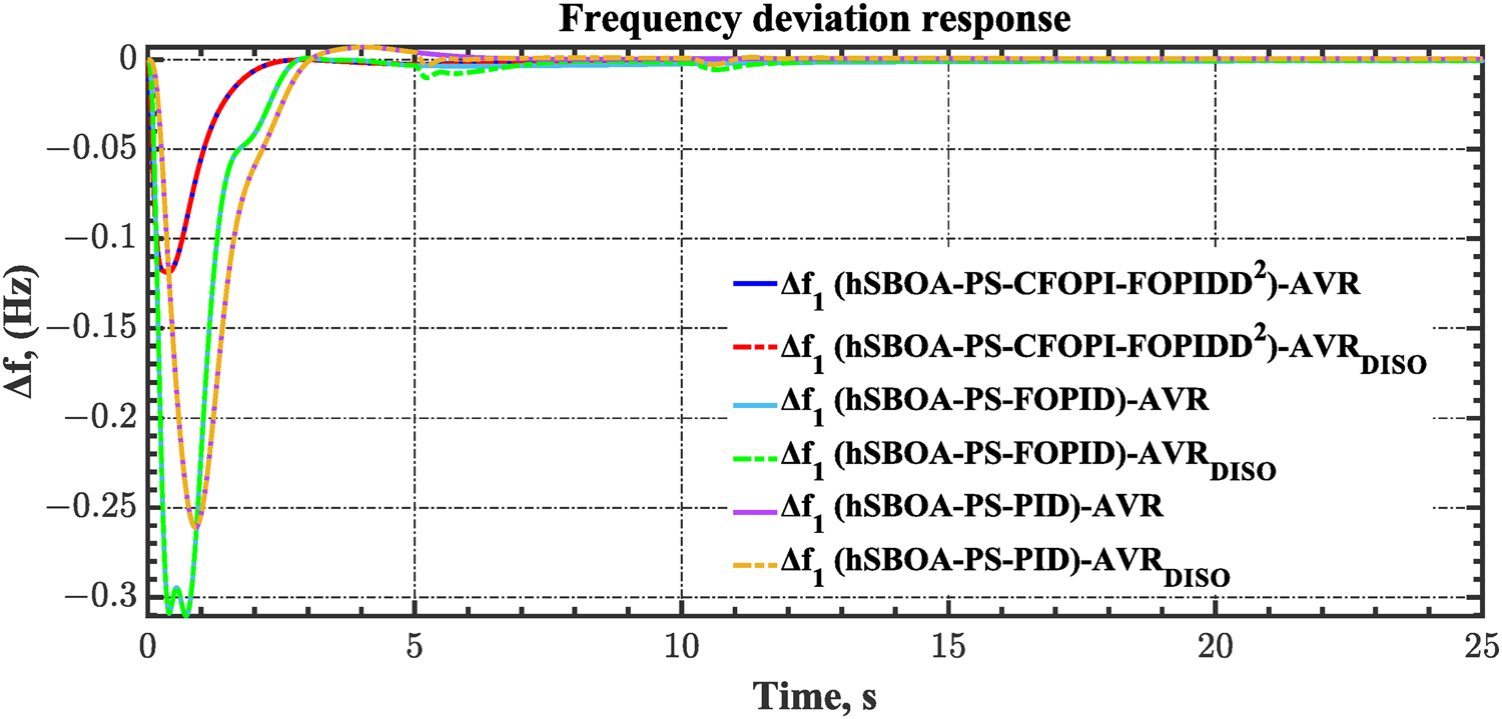
Area-1’s frequency deviation responses according to the seventh scenario.

**Fig. 47:**
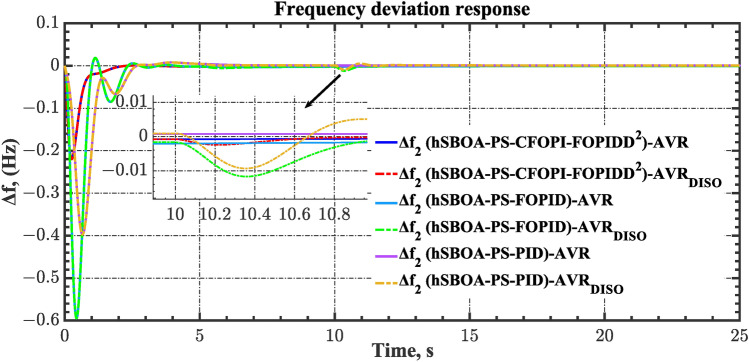
Area-2’s frequency deviation responses according to the seventh scenario.

**Fig. 48:**
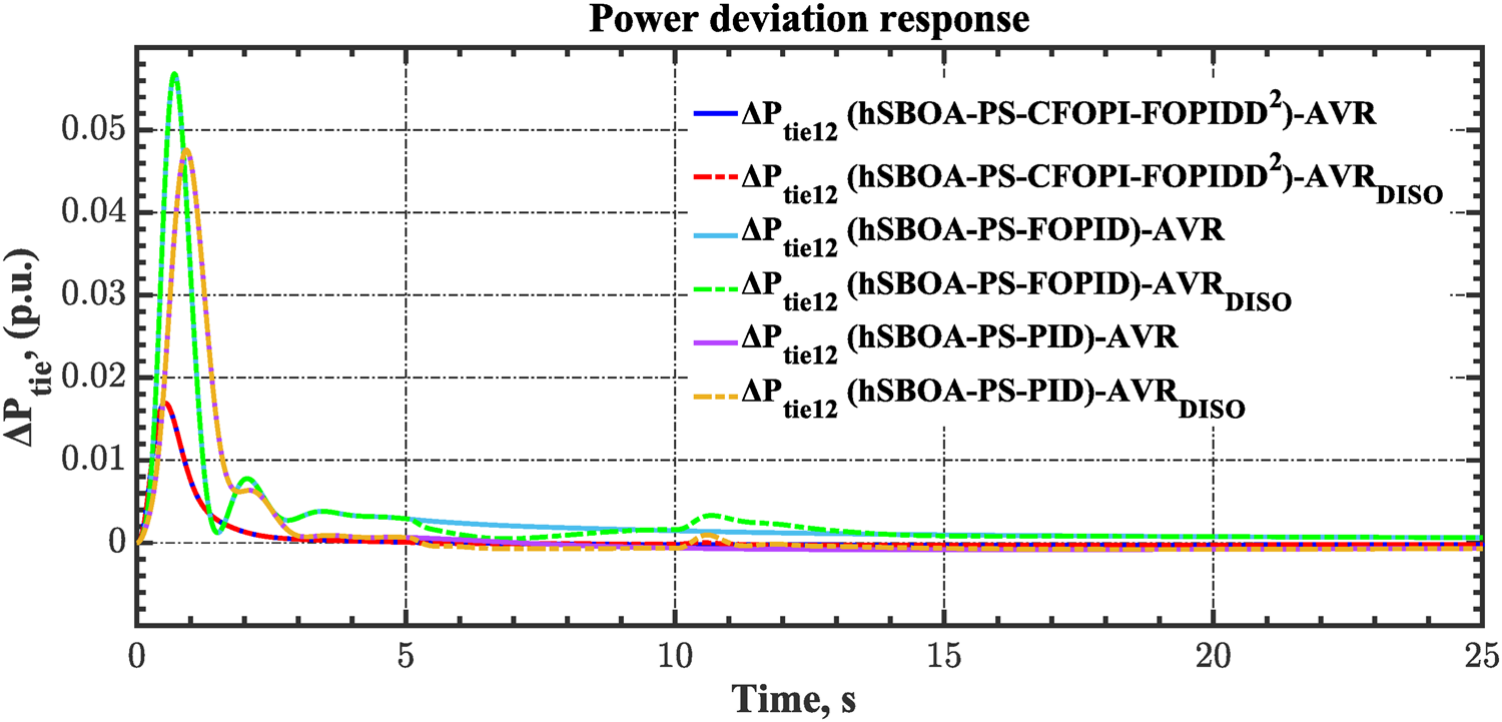
Power deviation responses according to the seventh scenario.

**Fig. 49:**
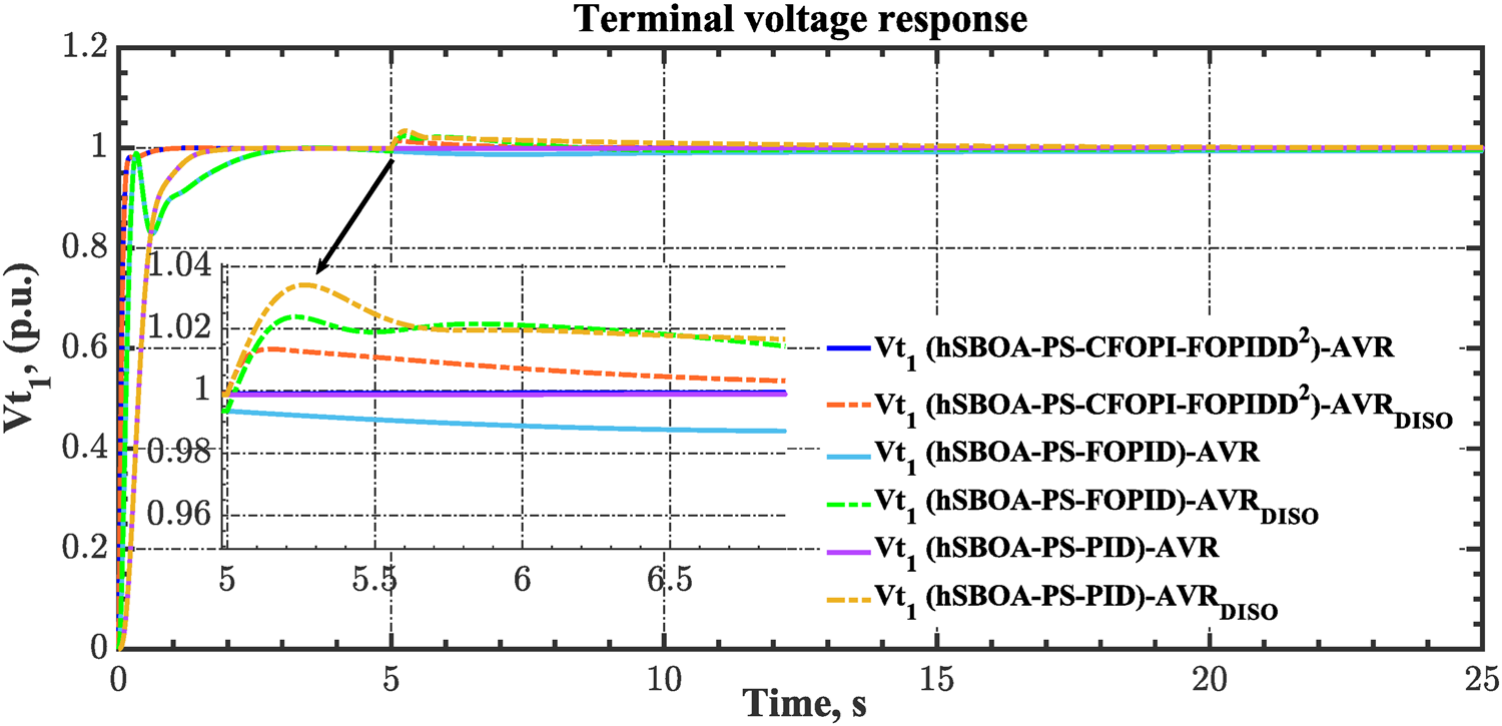
Terminal voltage responses for area-1 according to the seventh scenario.

**Fig. 50:**
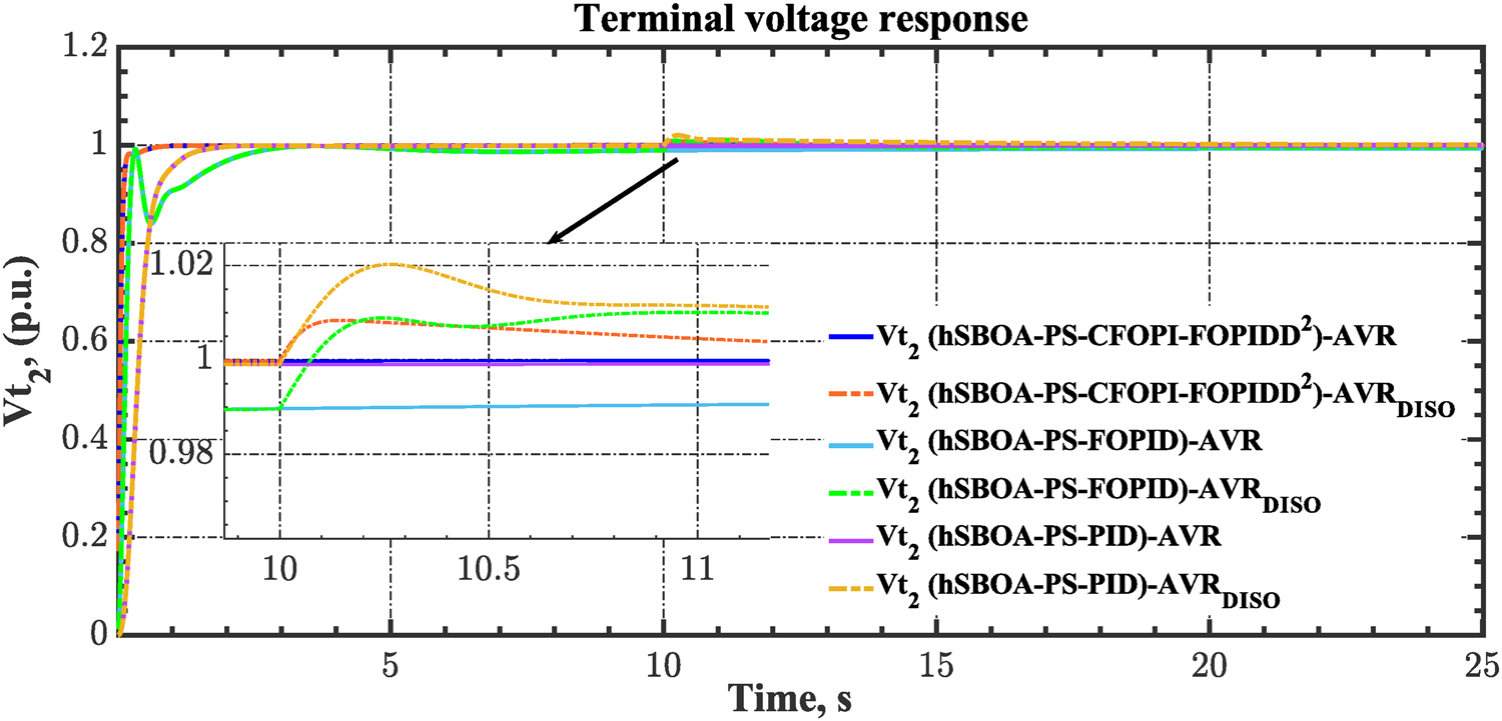
Terminal voltage responses for area-2 according to the seventh scenario.

The robustness of the proposed controllers against external disturbances in the AVR loop is investigated via the current case. Figure 45 demonstrates the block diagram for the AGC-AVR loops when AVR subjected to external disturbance. Here, this external disturbance is represented by ${V}_{Fa}$ signal which is an extra field voltage. The value of ${V}_{Fa}$ is 50% of the ${V}_{ref}$ at t = 5 s for area 1 and 30% of the ${V}_{ref}$ at t = 10 s for area 2. The ${V}_{ref}$ is considered as 1 p.u. for both areas. Also, in the current case, a 5% SLD is occurred in region two at t = 0 s. Additionally, the wind and solar units’ electricity generation are inaccessible. Table 13 summarizes the ITSE values obtained by different controllers under this scenario. On the other hand, the AGC-AVR loops’ dynamic responses are provided by Figs. 46, 47, 48, 49 and 50. Table 13 indicates that the hSBOA-PS-CFOPI-FOPIDD^2^ regulator achieved an ITSE value that is less than the FOPID regulator by 12.30 times and less than the PID regulator by 16.19, demonstrating its superior performance. It is clear that the both loops were affected by the disturbance that occurred in the AVR loop. However, the hSBOA-PS-CFOPI-FOPIDD^2^ controller had the ability to absorb the disturbance in a very fast time as shown in Figs. 46, 47, 48, 49 and 50. So, these figures thereby validate the robustness of the proposed IPS to the influence of varying generator and excitation voltage reference values.

**Table 13:** ITSE value under scenario 7 influence utilizing various controllers.

Controller	ITSE					The total ITSE
	${\Delta f}_{1}$	${\Delta f}_{2}$	${\Delta P}_{tie12}$	${\Delta V}_{1}$	${\Delta V}_{2}$	
hSBOA-PS-CFOPI-FOPIDD^2^	**0.005335**	**0.005475**	**0.0001154**	**0.002069**	**0.001863**	**0.01486**
hSBOA-PS-FOPID	0.05328	0.06654	0.001649	0.03001	0.03133	0.1828
hSBOA-PS-PID	0.05524	0.05689	0.001487	0.0667	0.0603	0.2406

The figures in bold are the best.

#### Scenario 8: System parameter variation impact

**Fig. 51:**
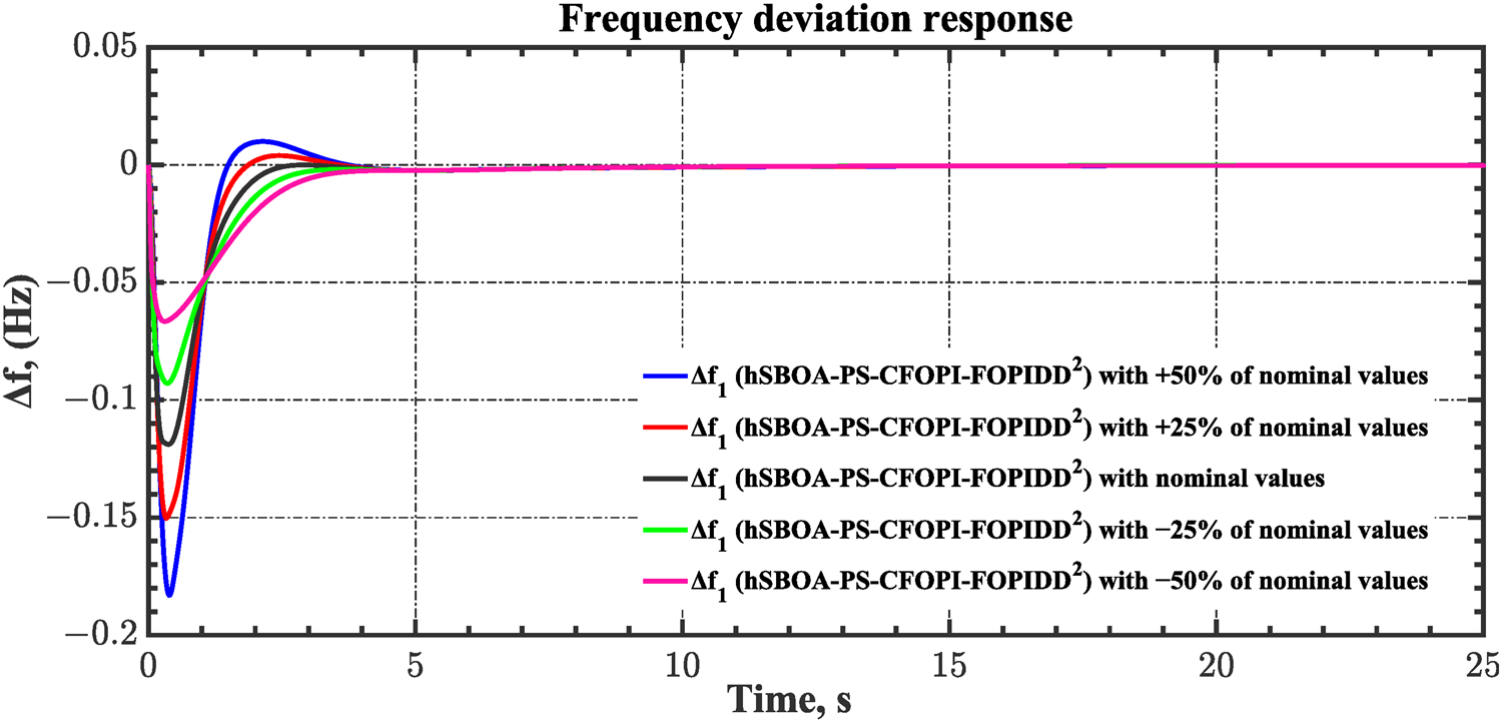
Area-1’s frequency deviation responses according to the eighth scenario.

**Fig. 52:**
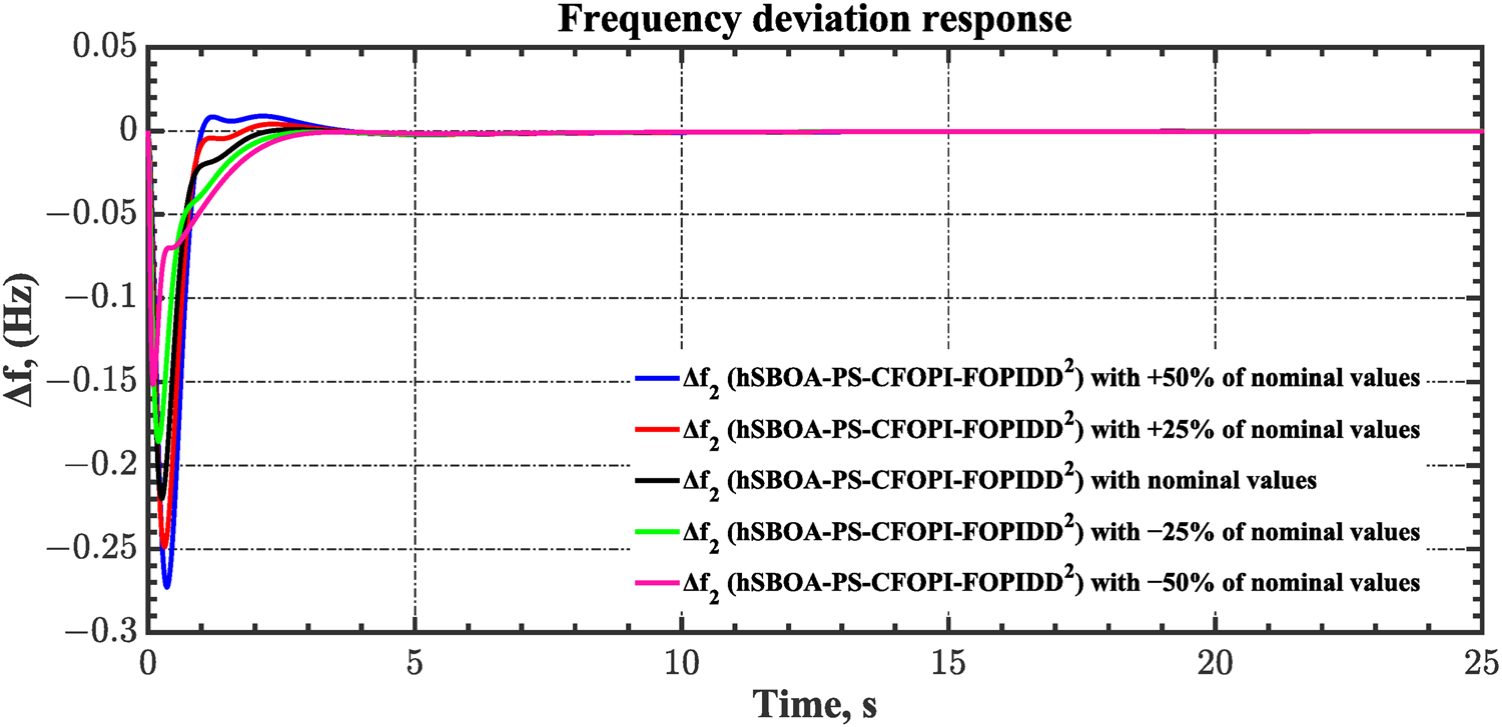
Area-2’s frequency deviation responses according to the eighth scenario.

**Fig. 53:**
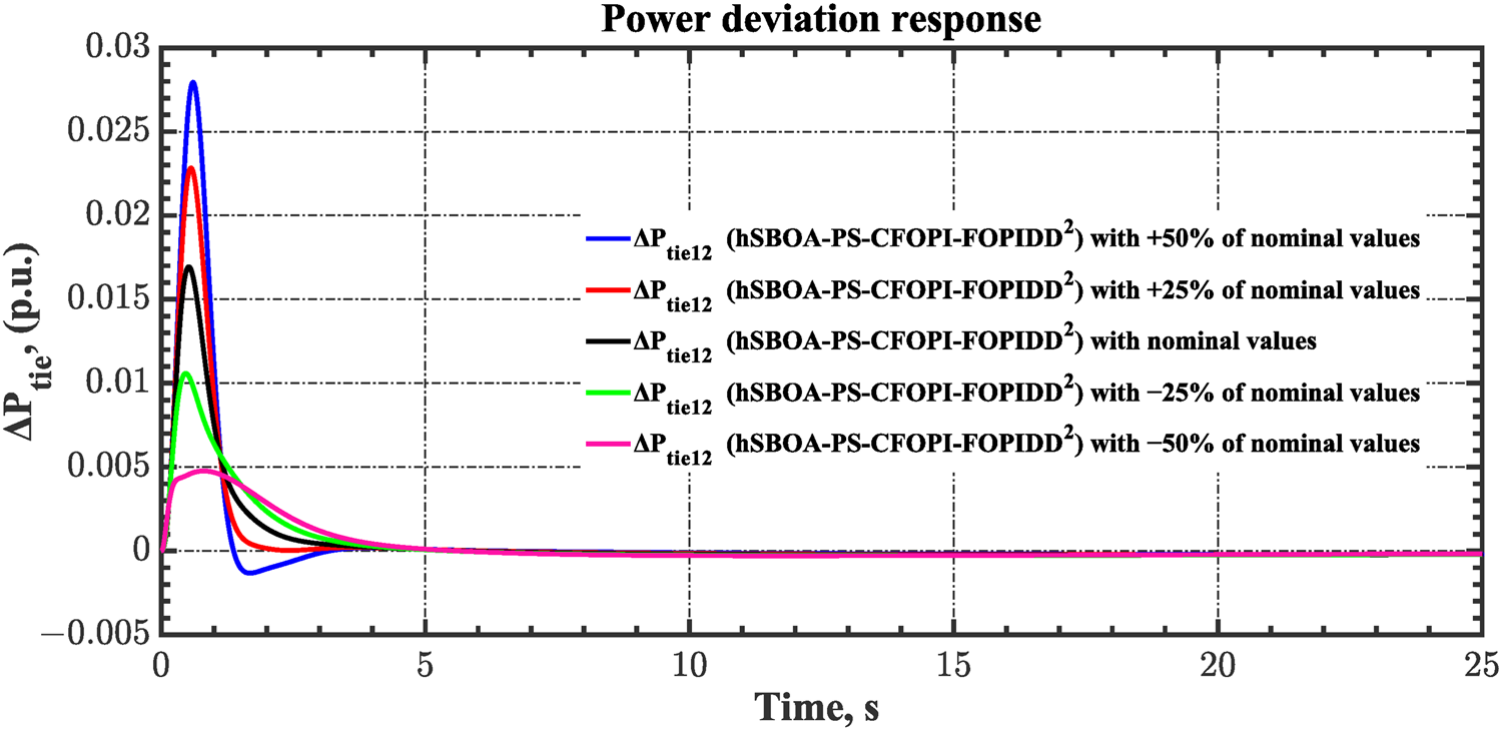
Power deviation responses according to the eighth scenario.

**Fig. 54:**
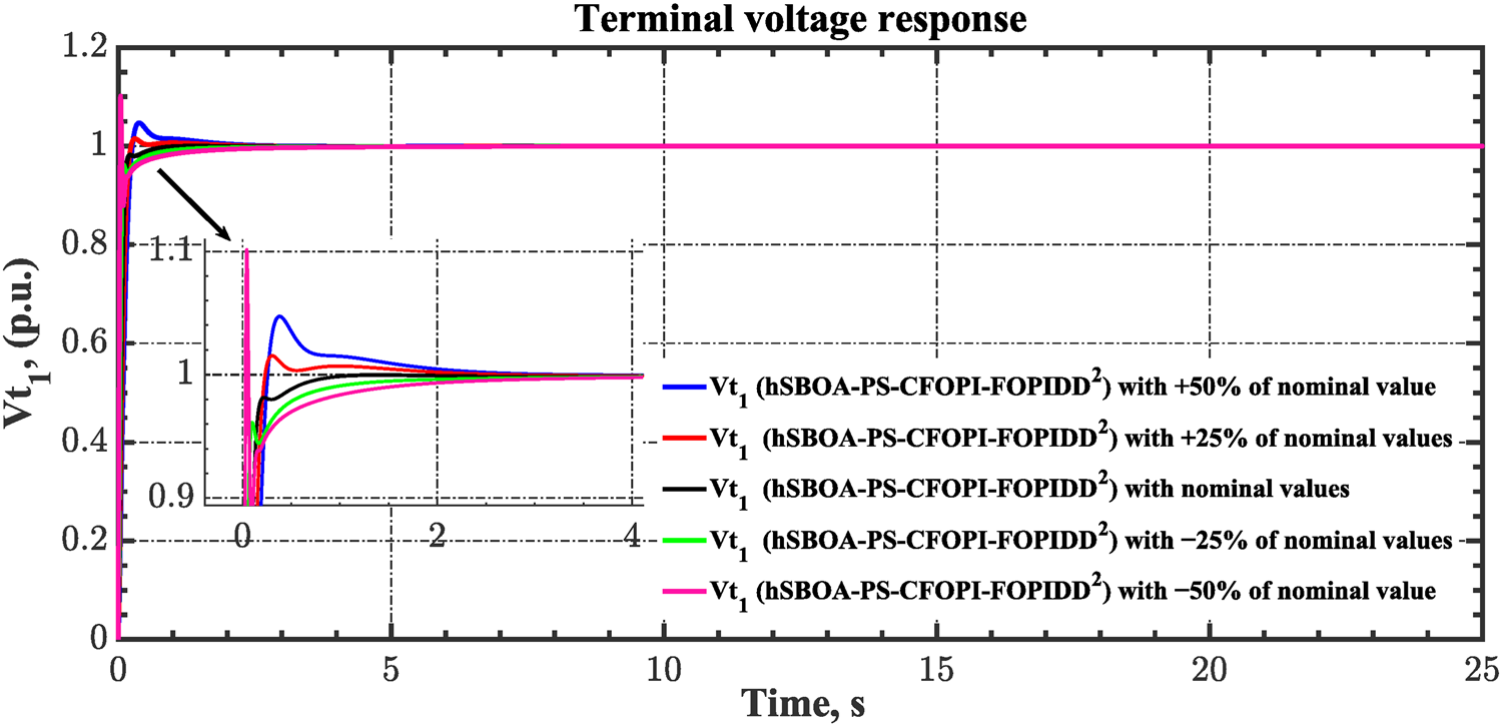
Terminal voltage responses for area-1 according to the eighth scenario.

**Fig. 55:**
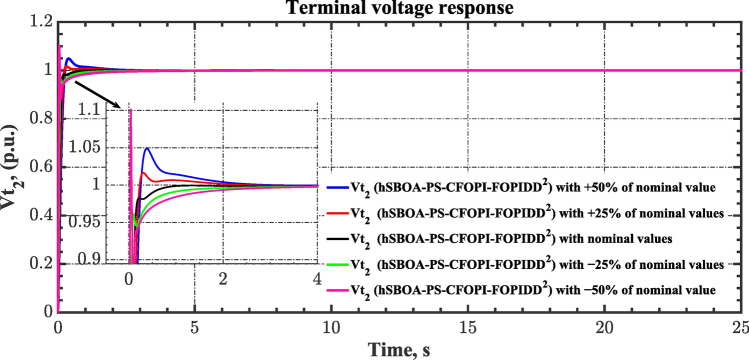
Terminal voltage responses for area-2 according to the eighth scenario.

Sensitivity analysis involves assessing how variations in system parameters or inputs affect the efficiency and stability of the AGC-AVR loops. So, in this scenario the first scenario conditions were used with varying some parameters from their nominal values. Here, the parameters ${T}_{tg}$, ${T}_{tt}$, ${T}_{hg}$, ${T}_{h2}$, ${T}_{h3}$, ${T}_{ev}$, ${K}_{LINE}$, ${T}_{a}$ and ${T}_{g}$ are varied by ± 50% from their nominal values. Table 14 summarizes the ITSE values obtained by different controllers under sensitivity analysis. Based on Table 14 the proposed controller showed its superiority over other controllers. It is observed that a reduction in the system parameters from their nominal values results in a decrease in the ITSE value, and conversely, an increase in the parameters results with the ITSE value rising. The dynamic responses of the AGC-AVR loops using the hSBOA-PS-CFOPI-FOPIDD^2^ controller are shown in Figs. 51, 52, 53, 54 and 55. These figures illustrate that the response speed of AGC-AVR system was similar across the five curves; however, there were variations in the $OS$ and $US$ values.

**Table 14:** ITSE value under scenario 8 influence utilizing various controllers (sensitivity analysis).

Controller	Parameter variation	ITSE					The total ITSE
		${\Delta f}_{1}$	${\Delta f}_{2}$	${\Delta P}_{tie12}$	${\Delta V}_{1}$	${\Delta V}_{2}$	
hSBOA-PS-CFOPI-FOPIDD^2^	Nominal	0.005329	0.005463	0.000115	0.001295	0.001291	0.01349
	− 50	0.003961	0.003499	0.00006	0.000932	0.000924	0.009376
	− 25	0.004396	0.003993	0.000084	0.0008912	0.000887	0.01025
	+ 25	0.007007	0.00781	0.000169	0.002268	0.002267	0.01952
	+ 50	0.009551	0.01098	0.000245	0.003957	0.003961	0.0287
hSBOA-PS-FOPID	Nominal	0.05299	0.06604	0.001601	0.04021	0.03949	0.2003
	− 50	0.0486	0.04458	0.000669	0.0421	0.04143	0.1774
	− 25	0.04863	0.05149	0.001041	0.04026	0.03965	0.1811
	+ 25	0.06057	0.08108	0.00229	0.04213	0.04126	0.2273
	+ 50	0.06992	0.09424	0.00298	0.04631	0.04528	0.2587
hSBOA-PS-PID	Nominal	0.05518	0.0565	0.001505	0.05397	0.05322	0.2204
	− 50	0.0514	0.0436	0.000581	0.04081	0.04021	0.1766
	− 25	0.05156	0.04633	0.000989	0.04629	0.04556	0.1907
	+ 25	0.06267	0.07069	0.002174	0.06405	0.06336	0.2629
	+ 50	0.07366	0.087	0.002937	0.07666	0.0761	0.3164

### Stability analysis

**Fig. 56:**
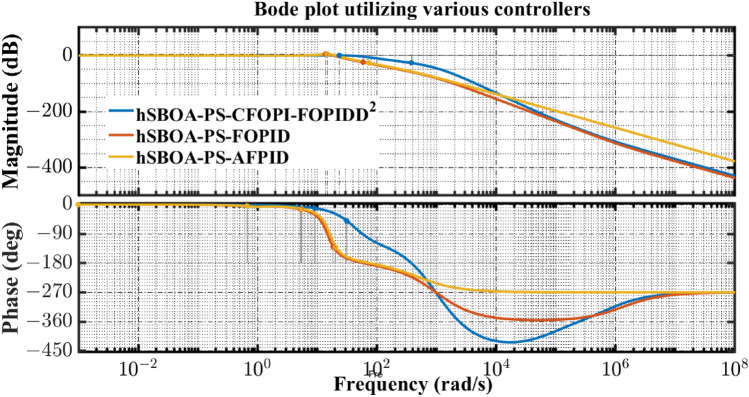
Bode diagram for controllers optimized via hSBOA-PS.

Here, the frequency stability of the proposed CFOPI-FOPIDD^2^ is evaluated through Bode plot analysis. The results of these analysis are shown by Fig. 56 and Table 15. As shown, the CFOPI-FOPIDD^2^ has the lowest peak gain, i.e. best damping and disturbance rejection. On the other hand, the AFPID has the highest phase margin (175°), suggesting extremely strong robustness, though it occurs at a very low frequency (0.671 rad/s), which could mean it’s slower to react to higher-frequency changes. Also, the CFOPI-FOPIDD^2^ has a high phase margin (169°) at a higher frequency (9.13 rad/s), suggesting a better balance of robustness and responsiveness. The CFOPI-FOPIDD^2^ has the lowest delay margin (0.322 s), i.e. less tolerant of delays, but this may be acceptable for fast, tightly-coupled systems. Moreover, the CFOPI-FOPIDD^2^ has the highest bandwidth, i.e. fastest and most agile control response. However, high bandwidth often comes at the cost of lower delay margin.

**Table 15:** The results obtained from the frequency domain analysis.

Controller	Peak gain (dB)	Phase margin (deg)	Delay margin (s)	Bandwidth (rad/s)	Stable
hSBOA-PS-CFOPI-FOPIDD^2^	0.341 (at 23.4 rad/s)	169 (at 9.13 rad/s)	0.322	52.6705	√
hSBOA-PS-FOPID	4.05 (at 13.6 rad/s)	165 (at 5.35 rad/s)	0.538	20.88	√
hSBOA-AFPID	4.44 (at 14.9 rad/s)	175 (at 0.671 rad/s)	4.55	23.10	√

### Real time validations

**Fig. 57:**
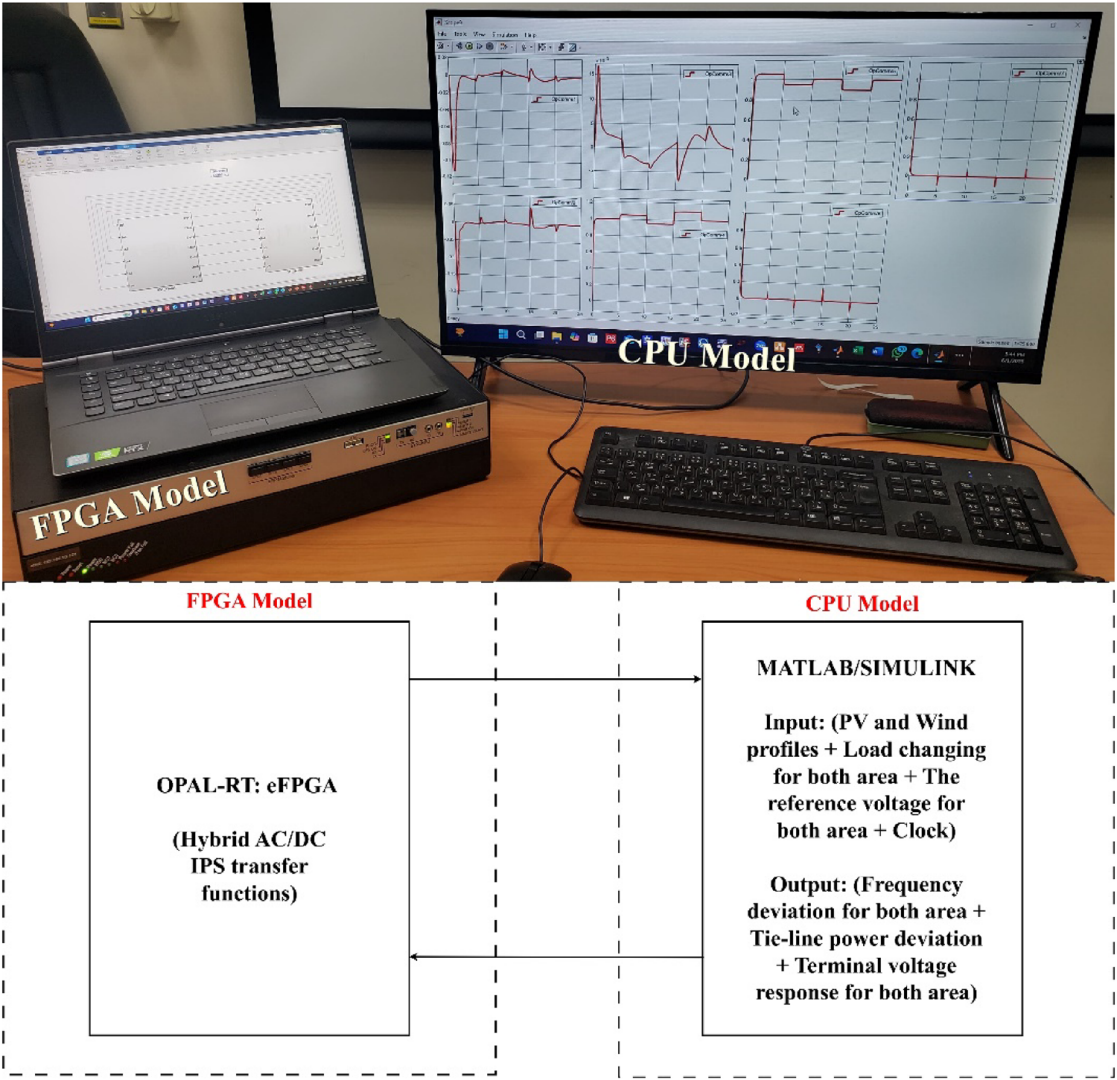
Experimental validation set-up.

**Fig. 58:**
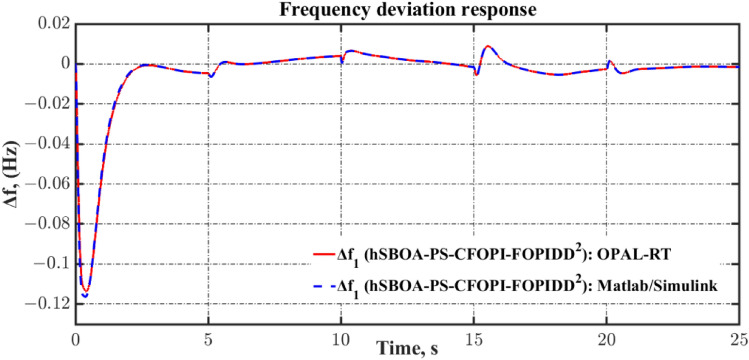
Area-1’s frequency responses according to the sixth scenario (real-time simulation).

**Fig. 59:**
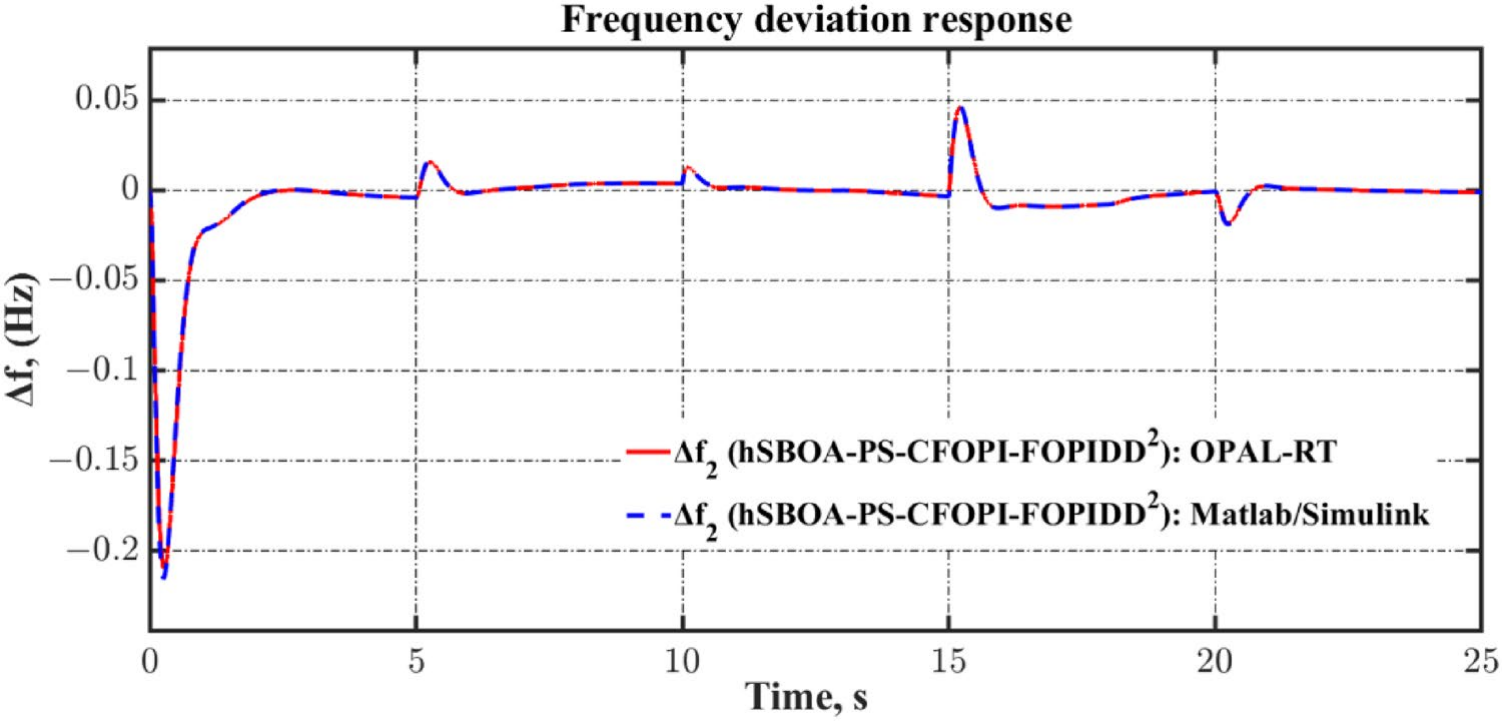
Area-2’s frequency deviation responses according to the sixth scenario (real-time simulation).

**Fig. 60:**
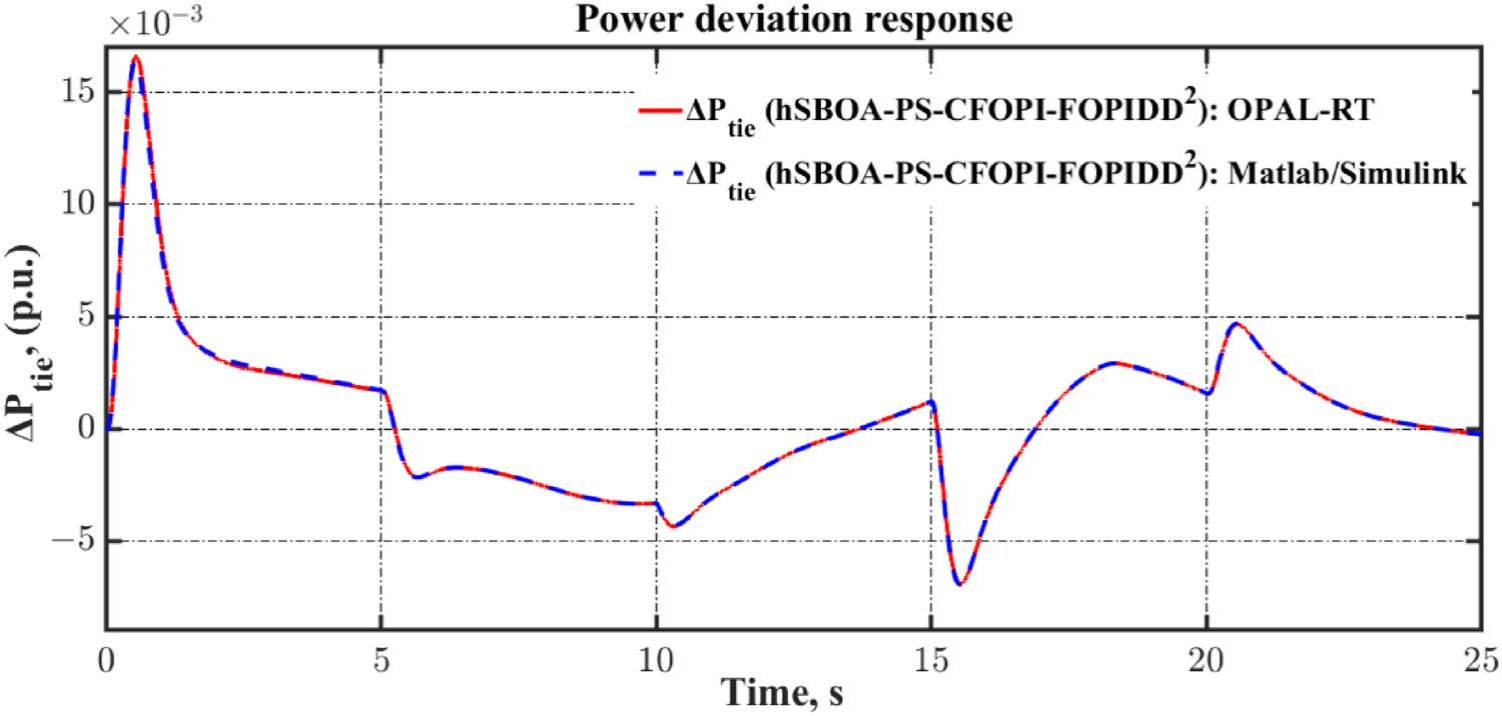
Power deviation responses according to the sixth scenario (real-time simulation).

**Fig. 61:**
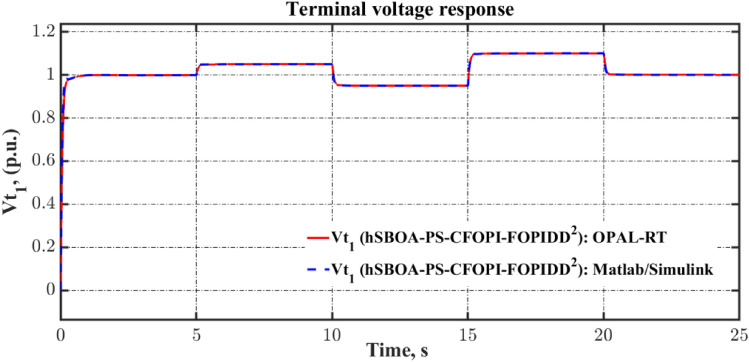
Terminal voltage responses for area-1 according to the sixth scenario (real-time simulation).

**Fig. 62:**
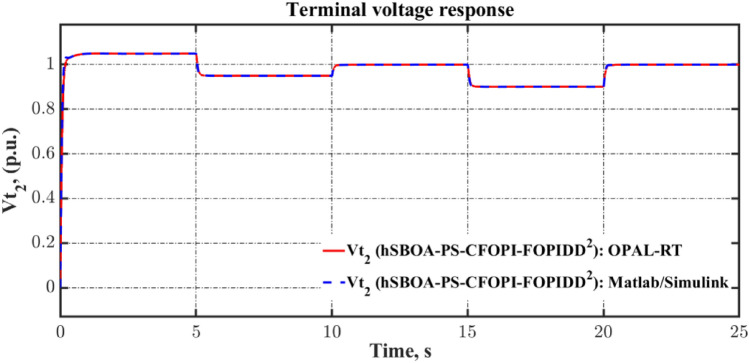
Terminal voltage responses for area-2 according to the sixth scenario (real-time simulation).

To validate the effectiveness of the proposed controller, a real-time simulation was conducted for Scenario 6. The OPAL-RT 4510 real-time simulator was employed to assess the performance of the proposed AGC-AVR strategy. The system model incorporating the hSBOA-PS-CFOPI-FOPIDD^2^ controller was developed using MATLAB/Simulink. The OPAL-RT subsequently takes over by converting the Simulink-based AGC-AVR model into a real-time executable program, which runs on its dedicated hardware. This facilitates real-time simulation, wherein the model operates synchronously with the actual time scale of a physical power system. Figure 57 illustrates the experimental setup. Figures 58, 59, 60, 61 and 62 depict the AGC-AVR dynamic response produced by the hSBOA-PS-CFOPI-FOPIDD^2^ controller under Scenario 6. As shown in Figs. 58, 59, 60, 61 and 62, the responses obtained from the real-time simulation match those derived from the MATLAB/Simulink environment, thereby confirming the efficiency of the proposed AGC-AVR strategy.

## Simulation results and discussion for test system-2

**Fig. 63:**
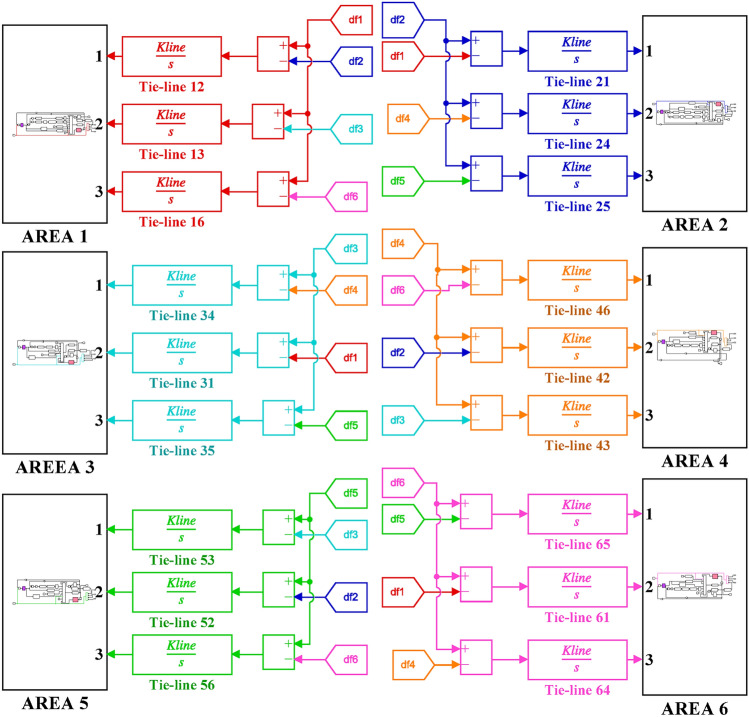
Simulink model of the Test System-2.

**Fig. 64:**
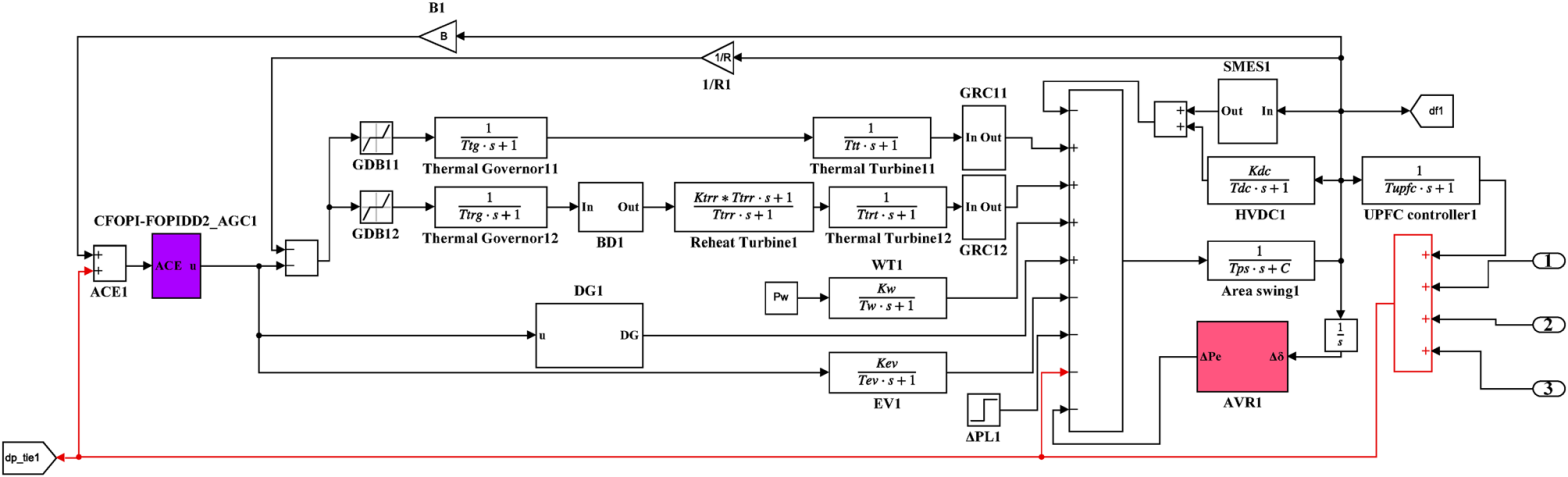
Simulink model for the Test System-2 (Region 1).

**Fig. 65:**
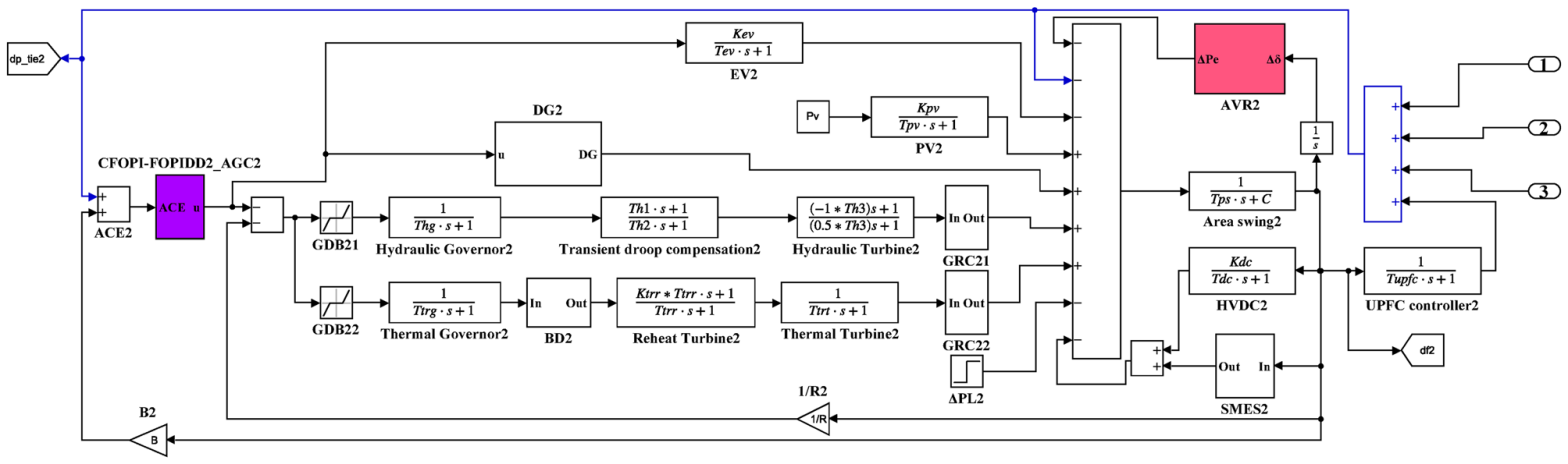
Simulink model for the Test System-2 (Region 2).

**Fig. 66:**
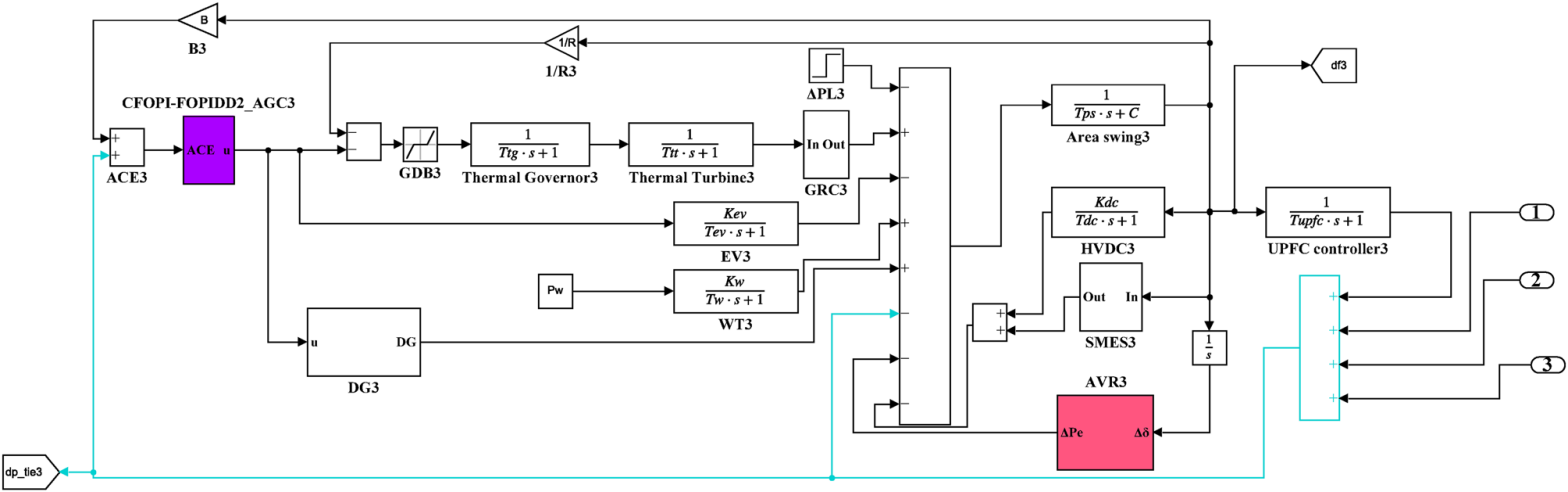
Simulink model for the Test System-2 (Region 3).

**Fig. 67:**
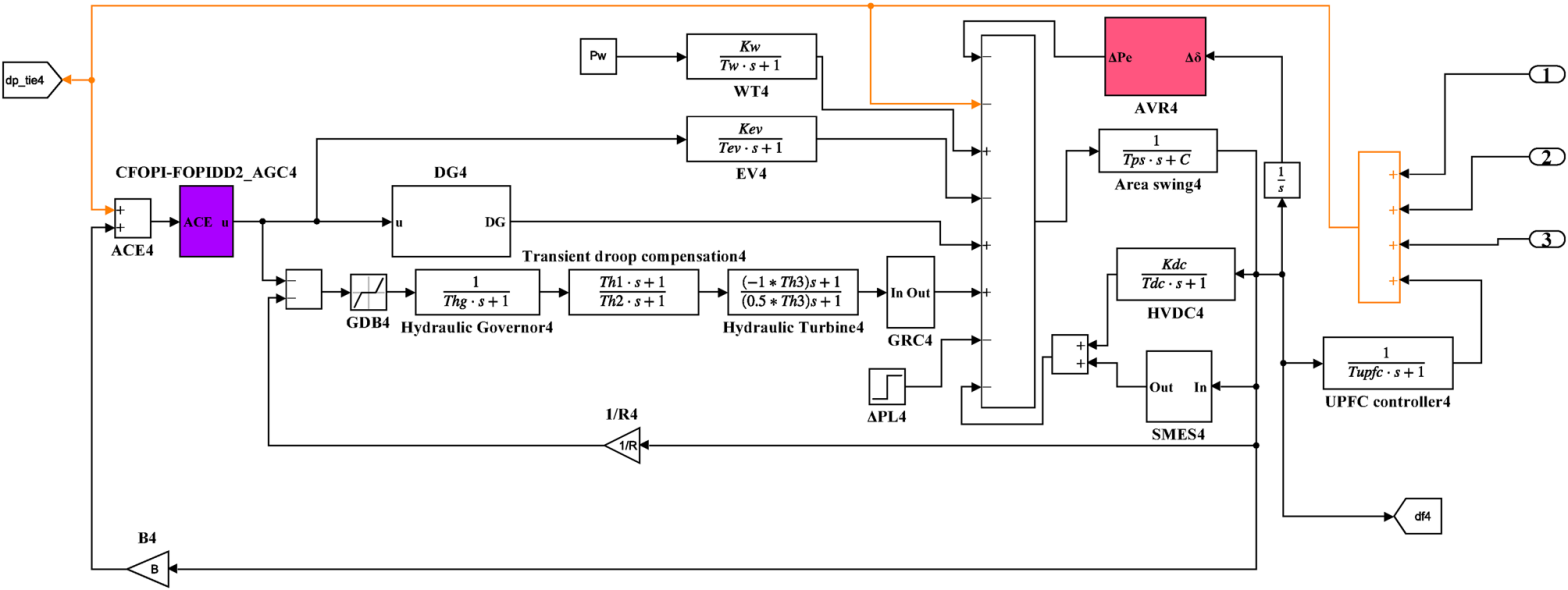
Simulink model for the Test System-2 (Region 4).

**Fig. 68:**
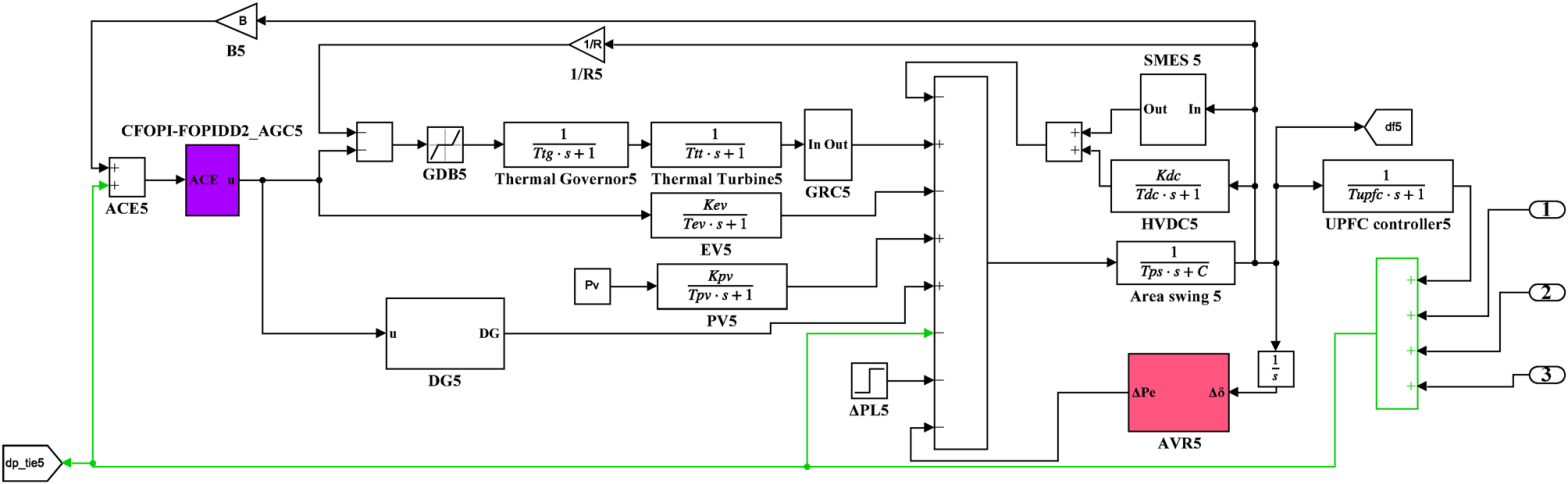
Simulink model for the Test System-2 (Region 5).

**Fig. 69:**
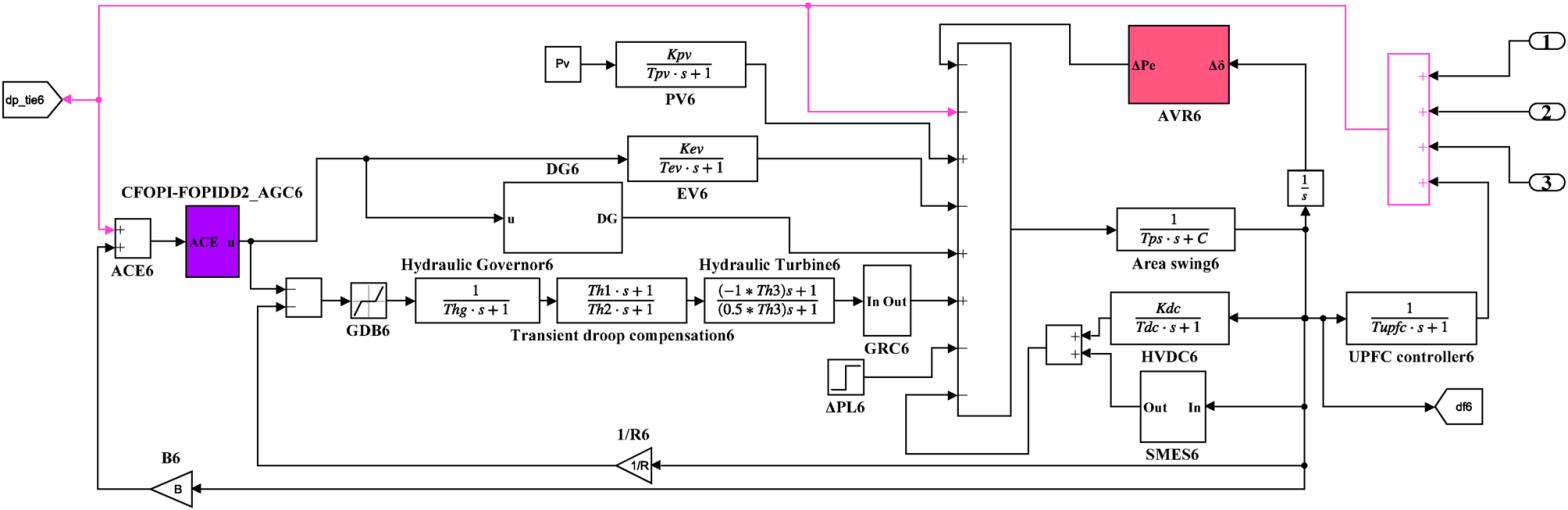
Simulink model for the Test System-2 (Region 6).

The Test System-2 that depicts in Fig. 63 is a nonlinear hybrid six-are multi-sources IPS, including RESs, ESSs and DG units. Area 1 contains non-reheat thermal unit with GDB and GRC, reheat thermal unit with GDB, BD and GRC, WT, DG units, EV, SMES, HVDC, and UPFC controller. Area 2 includes hydraulic unit with GDB and GRC, reheat thermal unit with GDB, BD and GRC, PV, DG units, EV, SMES, HVDC, and UPFC controller. Area 3 contains non-reheat thermal unit with GDB and GRC, WT, DG units, EV, SMES, HVDC, and UPFC controller. Area 4 includes hydraulic unit with GDB and GRC, WT, DG units, EV, SMES, HVDC, and UPFC controller. Area 5 contains non-reheat thermal unit with GDB and GRC, PV, DG units, EV, SMES, HVDC, and UPFC controller. Area 6 includes hydraulic unit with GDB and GRC, PV, DG units, EV, SMES, HVDC, and UPFC controller. The Simulink models of these areas are shown in Figs. 64, 65, 66, 67, 68 and 69. The transfer functions of the non-reheat thermal unit, hydraulic unit, WT, PV, EV, T-line, Area swing, $B$, $R$ and AVR system are shown in Table 1 while Table 16 shows the transfer functions of the reheat thermal unit, DG units^49^, SMES^50^, HVDC^40^, and UPFC controller^39^.

**Table 16:** Test System-2’s parameters.

Unit	Model	Transfer function	Parameter value
DG units	HAE	$\frac{{K}_{hae}}{{T}_{hae}\cdot s+1}$	${K}_{hae}=0.002$ and ${T}_{hae}=0.5\text{ s}$
	FESS	$\frac{{K}_{fess}}{{T}_{fess}\cdot s+1}$	${K}_{fess}=-0.01$ and ${T}_{fess}=0.1\text{ s}$
	FC	$\frac{{K}_{fc}}{{T}_{fc}\cdot s+1}$	${K}_{fc}=0.01$ and ${T}_{fc}=4\text{ s}$
	DEG	$\frac{{K}_{deg}}{{T}_{deg}\cdot s+1}$	${K}_{deg}=0.003$ and ${T}_{deg}=2\text{ s}$
	MTG	$\frac{{K}_{mtg}}{{T}_{mtg}\cdot s+1}$	${K}_{mtg}=1$ and ${T}_{mtg}=1.5\text{ s}$
Reheat thermal	Governor	$\frac{1}{{T}_{trg}\cdot s+1}$	${T}_{trg}=0.08\text{ s}$
	Reheat turbine	$\frac{\left({K}_{trr}\cdot {T}_{trr}\right)\cdot s+1}{{T}_{trr}\cdot s+1}$	${K}_{trr}=0.3$ and ${T}_{trr}=10\text{ s}$
	Steam turbine	$\frac{1}{{T}_{trt}\cdot s+1}$	${T}_{trt}=0.3\text{ s}$
SMES		$\frac{{T}_{1}\cdot s+1}{{T}_{2}\cdot s+1}$	${T}_{1}=0.2333\text{ s}$ and ${T}_{2}=0.016\text{ s}$
		$\frac{{T}_{3}\cdot s+1}{{T}_{4}\cdot s+1}$	${T}_{3}=0.7087\text{ s}$ and ${T}_{4}=0.2481\text{ s}$
		$\frac{{K}_{smes}}{{T}_{smes}\cdot s+1}$	${K}_{smes}=0.2035$ and ${T}_{smes}=0.03\text{ s}$
Other models	HVDC	$\frac{{K}_{dc}}{{T}_{dc}\cdot s+1}$	${K}_{dc}=1$ and ${T}_{dc}=0.2\text{ s}$
	UPFC controller	$\frac{1}{{T}_{upfc}\cdot s+1}$	${T}_{upfc}=0.1\text{ s}$

**Fig. 70:**
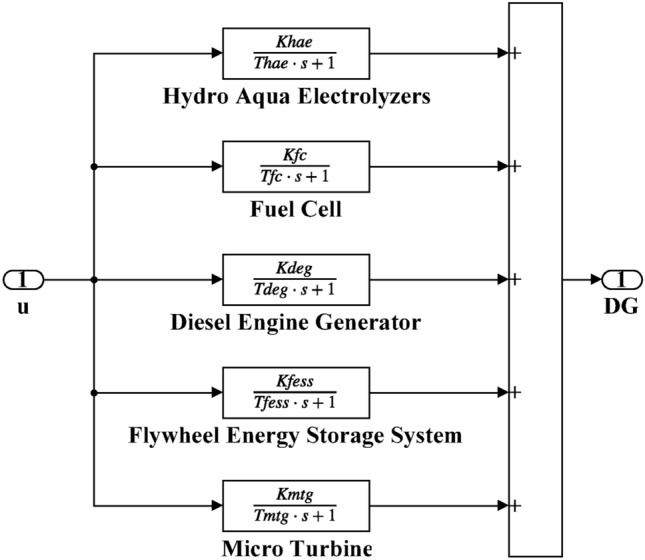
DG System’s Simulink model.

DGs denote a range of technologies that produce electricity at or in proximity to the point of consumption. They play a vital role in providing clean and reliable power to more customers while minimizing electricity losses during transmission and distribution. Here, the DG system involves hydro-aqua electrolyzers (HAE), FESS, fuel cells (FC), diesel engine generators (DEG), and microturbines (MTG). Figure 70 depicts the DG system block diagram.

**Fig. 71:**
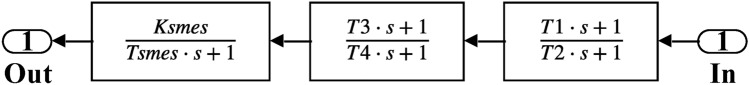
Simulink model of the SMES system.

ESSs such as BESS, FESS and SMES are implemented in the power grid to retain surplus energy produced by power plants. SMES facilitates the augmentation of generated energy to reduce the stabilization time of the system. So, compared with BESS the SMES is better for AGC system^50^. The SMES system block diagram is shown in Fig. 71.

The AGC-AVR designing is improved by FACTS such as Advanced Thyristor Controlled Series Capacitor (ATCSC)^51^ and UPFC controller^39^. Here, the UPFC controller is used. The UPFC is considered to be among the FACTS’ most flexible devices. It can improve transient stability and reduce system oscillations^52^. Also, the HVDC is used to enhance the reliability and functionality of the IPS^40^.

**Fig. 72:**
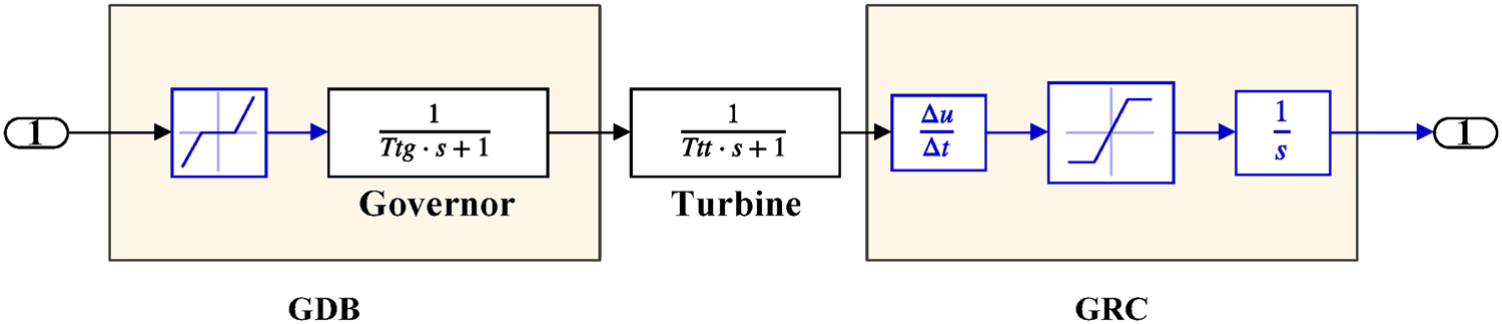
Simulink model of the GDB and GRC.

**Fig. 73:**
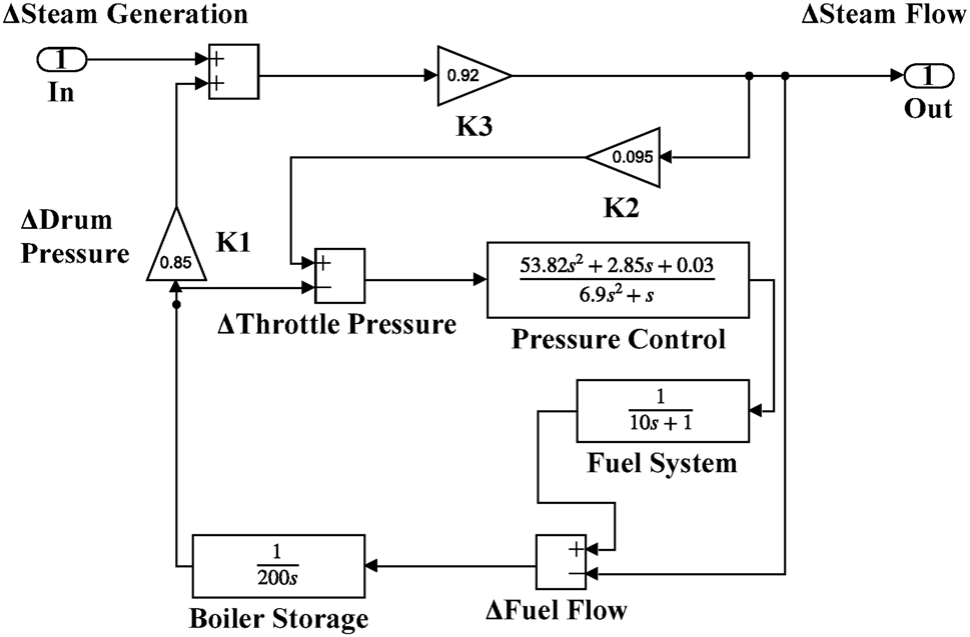
Simulink model of the BD System.

The limitations of thermal and mechanical movements in IPS including the GRC, GDB, BD are affecting the performance of the AGC-AVR loops. Here, these three limitations are included in the test system. GDB is a metric for any steady state velocity changes that don’t include the governor valve^33^. To put it another way, the GDB is the range that the AGC may allow the frequency to vary without implementing any controls^3^. In the current investigation, a GDB of ± 0.0336 Hz is used for thermal units and a GDB of ± 0.012 Hz is used for hydraulic units. In steam power plants, power generation can only change at a predetermined maximum rate known as GRC. If this rate is disregarded, the AGC system may experience disruptions and substantial transient changes^3^. The GRC for hydropower plants is 270% per minute during high generation and 360% per min during low generation. In contrast, the GRC for thermal power plants is capped at 3% per min^33^. Here, a GRC of 3% per min is used for thermal units, and a GRC of 270% per min is used for hydraulic units. The GDB and GRC block diagrams are shown in Fig. 72. Turbine control valves are used in the majority of steam power plants for implement modifications in generation. Upon detecting changes in pressure and steam flow, the boiler control system promptly implements the requisite modifications. The transfer function for BD is illustrated in Fig. 73^33^.

### hSBOA-PS testing for test system-2

The purpose of this testing is to show the ability of hSBOA-PS for finding the excellent ${K}_{\text{p}1}$, ${K}_{\text{i}1}$, ${K}_{p2}$, ${K}_{i2}$, ${K}_{\text{d}1}$, ${K}_{d2}$, ${\uplambda }_{1}$, ${\uplambda }_{2}$, ${\mu }_{1}$ and ${\mu }_{2}$ that obtain the minimum $\text{Obj}$. The optimization is done based on the scenario of 5% SLD occurs in region 2 at t = 0 s with TFTV equals to 1 p.u for the Six Areas. Furthermore, the RESs units’ power generation is considered as a uniform profile and accessible with 20% for area 1, 25% for area 2, 15% for area 3, 25% for area 4, 15% for area 5 and 20% area 6. The controllers’ parameters which hSBOA-PS achieved are listed in Table 17. To minimize the decision variables, identical controller parameters are employed for the AVR loop throughout the six areas. Additionally, identical controller parameters are used for the AGC loop in areas 1, 3, and 5 (thermal units). In contrast, identical controller parameters are used for the AGC loop in areas 2, 4, and 6 (hydraulic units). Table 18 shows the comparison between CFOPI-FOPIDD^2^ and FOPID controllers based on ITSE and $\text{Obj}$. According to Table 18, the proposed controller produced an $\text{Obj}$ value which is roughly 1.77 times less than the FOPID regulator and an ITSE that is 3.67 times lower.

**Table 17:** Suggested parameters for the two controllers gotten via hSBOA-PS for Test System-2.

Controller	${K}_{\text{p}1}$/${K}_{\text{p}}$	${K}_{\text{i}1}$/${K}_{\text{i}}$	${K}_{p2}$	${K}_{i2}$	${K}_{\text{d}1}$/${K}_{\text{d}}$	${K}_{d2}$	${\lambda }_{1}$	${\uplambda }_{2}$	${\mu }_{1}$	${\mu }_{2}$
AGC (Area 1, 3, and 5)										
CFOPI-FOPIDD^2^	1.9942	1.9967	1.9993	1.9973	1.9449	0.0956	0.8926	0.3371	0.9483	0.95
FOPID	1.5676	2	–	–	1.4272	–	0.95	–	0.8687	–
AGC (AREA 2, 4, and 6)										
CFOPI-FOPIDD^2^	1.9923	1.7415	1.9762	0.1025	1.9987	0.0992	0.6887	0.2687	0.95	0.9486
FOPID	1.9882	1.9905	–	–	1.9987	–	0.6776	–	0.9464	–
AVR (All areas)										
CFOPI-FOPIDD^2^	1.9468	1.2499	1.6584	0.1006	1.1217	0.1	0.95	0.4057	0.95	0.95
FOPID	1.1260	0.6845	–	–	0.5838	–	0.95	–	0.95	–

**Table 18:** ITSE and Obj values using CFOPI-FOPIDD^2^ and FOPID controllers under hSBOA-PS testing.

Controller	ITSE	$\text{Obj}$
hSBOA-PS-CFOPI-FOPIDD^2^	**0.0899**	**398.8345**
hSBOA-PS-FOPID	0.3299	704.8574

The figures in bold are the best.

### Simulation results for test system-2

**Fig. 74:**
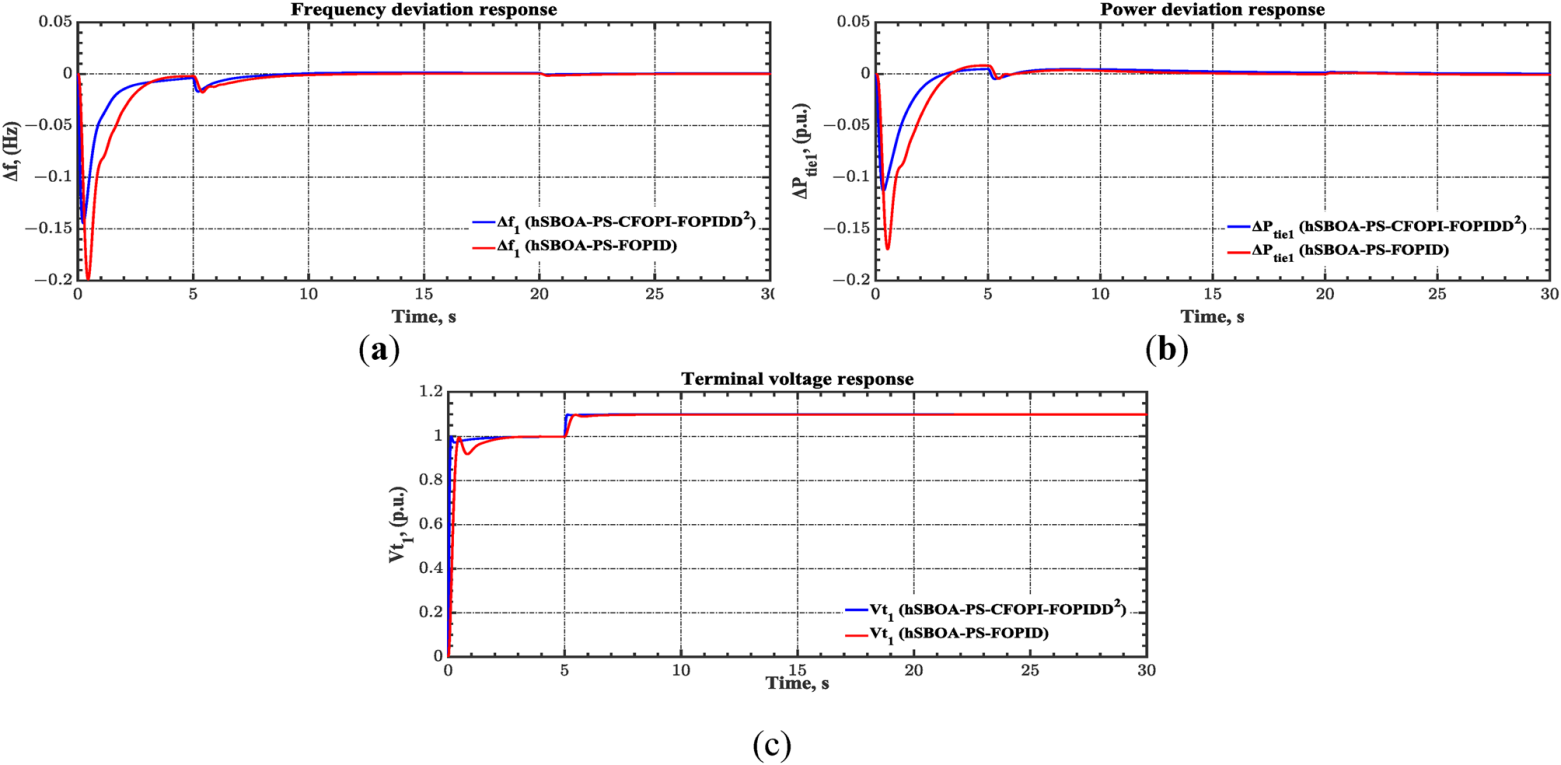
Dynamic response curves of the Test System-2: (**a**) Area-1’s frequency deviation; (**b**) Area-1’s power deviation; (**c**) Area-1’s terminal voltage.

**Fig. 75:**
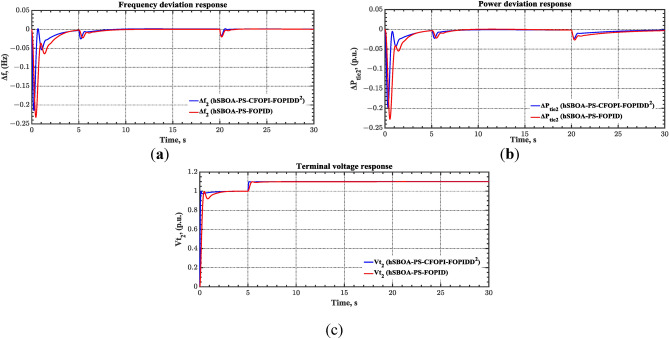
Dynamic response curves of the Test System-2: (**a**) Area-2’s frequency deviation; (**b**) Area-2’s power deviation; (**c**) Area-2’s terminal voltage.

**Fig. 76:**
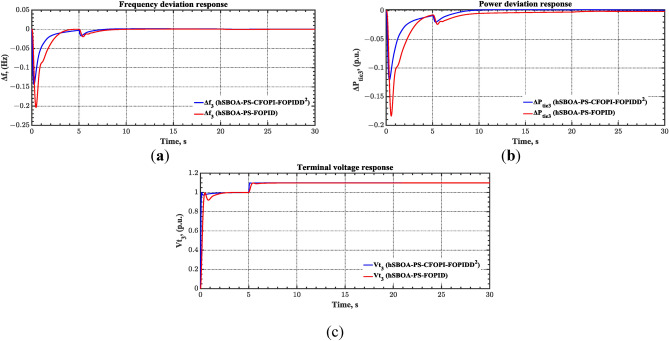
Dynamic response curves of the Test System-2: (**a**) Area-3’s frequency deviation; (**b**) Area-3’s power deviation; (**c**) Area-3’s terminal voltage.

**Fig. 77:**
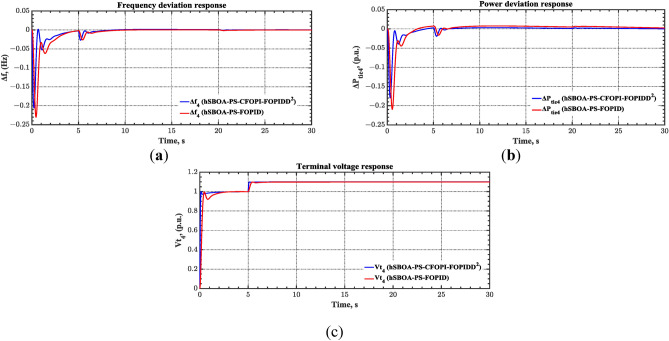
Dynamic response curves of the Test System-2: (**a**) Area-4’s frequency deviation; (**b**) Area-4’s power deviation; (**c**) Area-4’s terminal voltage.

**Fig. 78:**
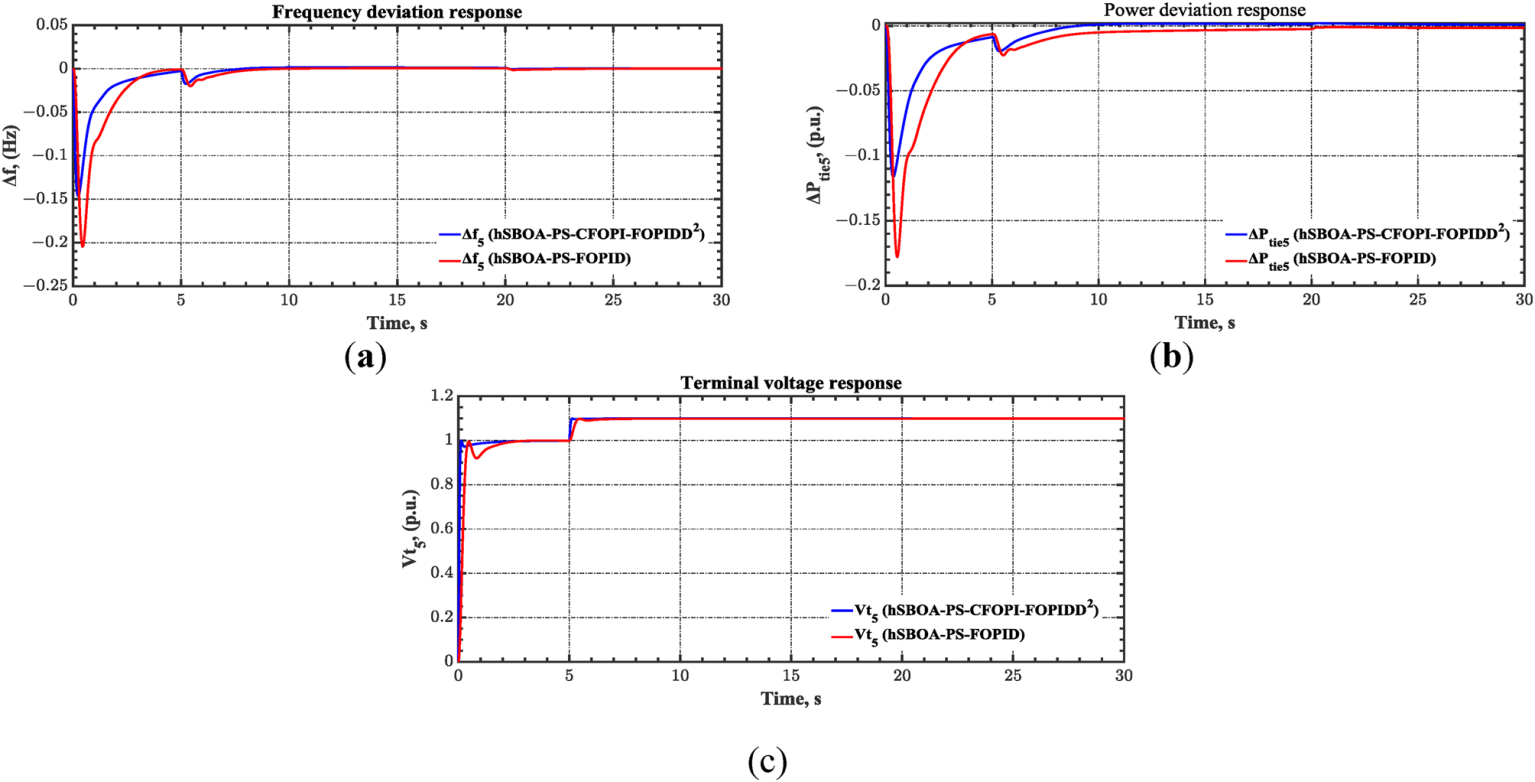
Dynamic response curves of the Test System-2: (**a**) Area-5’s frequency deviation; (**b**) Area-5’s power deviation; (**c**) Area-5’s terminal voltage.

**Fig. 79:**
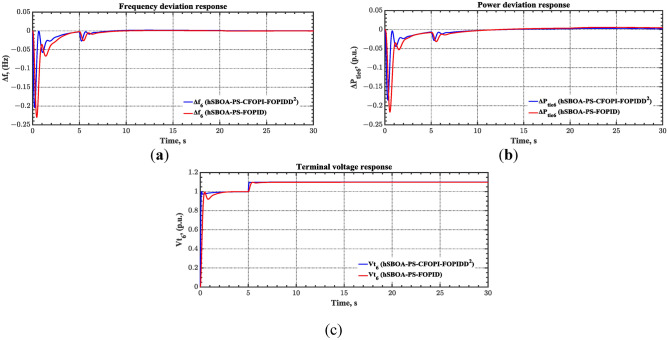
Dynamic response curves of the Test System-2: (**a**) Area-6’s frequency deviation; (**b**) Area-6’s power deviation; (**c**) Area-6’s terminal voltage.

The simulation is done based on the scenario of 5% SLD occurs in region two at t = 0 s then this SLD is increased to 10% at t = 20 s. The TFTV is equaled 1 p.u. then it is increased to 1.1 p.u. at t = 5 s for all areas. Furthermore, the RESs units’ power generation is considered as a uniform profile and accessible with 20% for area 1, 25% for area 2, 15% for area 3, 25% for area 4, 15% for area 5 and 20% area 6. Table 19 summarizes the ITSE values obtained by CFOPI-FOPIDD^2^ and FOPID controllers under this simulation. Figures 74, 75, 76, 77, 78 and 79 display the system’s dynamic responses. According to Table 19, the proposed controller achieved the total ITSE value approximately 3.4 times lower than that of the FOPID controller. ITSE is an index that measures the time-varying output error and converges to 0 when the control is stable. Therefore, the purpose of the CFOPI-FOPIDD^2^ controller is to obtain the lowest ITSE value as well as best AGC-AVR system response. Also, Figs. 74, 75, 76, 77, 78 and 79 demonstrate that the usage of the CFOPI-FOPIDD^2^ regulator led to reduced peak amplitudes of the deviations.

**Table 19:** ITSE value using CFOPI-FOPIDD^2^ and FOPID controllers under simulation testing.

ITSE	hSBOA-PS-CFOPI-FOPIDD^2^	hSBOA-PS-FOPID
Area 1		
${\Delta f}_{1}$	**0.006311**	0.01688
${\Delta P}_{tie1}$	**0.007357**	0.0171
$\Delta {Vt}_{1}$	**0.002853**	0.02471
Area 2		
${\Delta f}_{2}$	**0.008511**	0.0188
${\Delta P}_{tie2}$	**0.01813**	0.04111
${\Delta Vt}_{2}$	**0.002851**	0.02467
Area 3		
${\Delta f}_{3}$	**0.00675**	0.01844
${\Delta P}_{tie3}$	**0.01185**	0.03297
${\Delta Vt}_{3}$	**0.002845**	0.02472
Area 4		
${\Delta f}_{4}$	**0.007176**	0.01678
${\Delta P}_{tie4}$	**0.006464**	0.02571
$\Delta {Vt}_{4}$	**0.002853**	0.02472
Area 5		
${\Delta f}_{5}$	**0.006882**	0.01837
${\Delta P}_{tie5}$	**0.01139**	0.03055
${\Delta Vt}_{5}$	**0.002842**	0.02468
Area 6		
${\Delta f}_{6}$	**0.007518**	0.01756
${\Delta P}_{tie6}$	**0.01139**	0.02861
$\Delta {Vt}_{6}$	**0.002848**	0.02472
The Total ITSE	**0.1268**	0.4311

The figures in bold are the best.

It is clear from Figs. 74, 75, 76, 77, 78 and 79, that the dynamic response with BD, GRC and GDB nonlinearity has a long $ST$ and a great $US$ in particular in the AGC loop. Both controllers performed well, however the CFOPI-FOPIDD^2^ controller outperformed the FOPID controller.

## Conclusions

The goal of the presented paper is to propose an efficient designing for AGC-AVR loops in a hybrid AC/DC IPS with nonlinear structures. In this regard, a novel controller configuration CFOPI-FOPIDD^2^ was proposed. Further, a novel hybrid algorithm named hSBOA-PS which is based on SBOA and PS was used to optimize the controller’s settings via assistance of a fitness objective function. This fitness function was created using the dynamic response characteristics such as ITSE, OS, US, and ST. The CFOPI-FOPIDD^2^ controller enhances AGC-AVR performance by 60.5% over the DO-FOPI-PIDD^2^ controller and 81% AFPID controller. Moreover, a comparison analysis was carried out between the CFOPI-FOPIDD^2^, PID, and FOPID controllers under various conditions. As expected, CFOPI-FOPIDD^2^ showed its superiority over those controllers. Unlike previous studies, the proposed approach was examined in the AGC-AVR system where AVR as DISO and in a hybrid six-area IPS with nonlinearity. The outcomes demonstrated that the suggested approach can handle these types of IPS effectively. In future work, the proposed CFOPI-FOPIDD^2^ controller for AGC-AVR loops will be evaluated within a large-scale IMG. Furthermore, an AVR model with a Multi-Input Single-Output (MISO) configuration will also be incorporated. Also, our future research can be directed toward incorporating the state of charge (SOC) of EVs to more accurately reflect their dynamic behavior in control strategies. In this paper, the EV gain is assumed to be constant. However, when the battery’s SOC is considered, the EV gain becomes time-varying, which may influence the controller’s performance.

## Supplementary Information

Supplementary Information 1.

Supplementary Information 2.

## Data Availability

The wind and PV real profiles used in this work are available in references^48,49^. The models of fractional-order transfer functions are taken from the FOMCON Toolbox for MATLAB and are available here: https://www.mathworks.com/matlabcentral/fileexchange/66323-fomcon-toolbox-for-matlab. The code of the PS method is taken from the MATLAB Global Optimization Toolbox and is available here: https://www.mathworks.com/help/gads/patternsearch.html. Other data generated or analysed during this study are included in this published article.

## List of symbols

CFOPI-FOPIDD^2^: A cascaded fractional order proportional integral-fractional order proportional integral derivative double derivative
ITSE: Integral of time square error
AGC: Automatic generation control
AVR: Automatic voltage regulator
IPS: Interconnected power system
PID: Proportional-integral-derivative
FOPID: Fractional order PID
RESs: Renewable energy sources
ESSs: Energy storage systems
EVs: Electric vehicles
HVDC: High voltage direct current
MGs: Microgrids
SGs: Smart grids
DGs: Distributed generations
FACTS: Flexible AC transmission systems
UPFC: Unified power flow controller
SEMS: Superconducting magnetic energy storage
BESS: Battery energy storage system
FESS: Flywheel energy storage system
PV: Photovoltaics
WEG: Wind energy generators
GRC: Generation rate constraint
GDB: Governor dead band
BD: Boiler dynamics
CTD: Communication time delay
SISO: Single input single output
DISO: Double input single output
SBOA: Secretary bird optimization algorithm
PS: Pattern search
hSBOA-PS: Hybrid optimization method using SBOA and PS
WT: Wind farm
SLD: Step load disturbance
TFTV: Time-fixed desired voltage
RLD: Random load disturbance
TVDV: Time-varying desired voltage
LDC: Load dispatch centre
HAE: Hydro-aqua electrolyzers
FC: Fuel cells
DEG: Diesel engine generators
MTG: Microturbines
+ H: High-positive
$\text{ACE}$: Area control error
${B}_{i}$: The frequency bias parameter (p.u./Hz)
$\Delta {f}_{i}$: The change in frequency (Hz)
${R}_{i}$: The governor speed regulation parameter (Hz/p.u.)
${\Delta P}_{refi}$: The output from the controller of the AGC loop
$\Delta {P}_{Li}$: The load demand change (p.u.)
$\Delta {P}_{tie ij}$: The incremental change in tie line power (p.u.)
${U}_{AGCi}$: The controller signal of the AGC loop
$i$ and $j$: Subscripts referred to the corresponding area
${V}_{ti}$: The generator terminal voltage
${V}_{refi}$: The reference voltage
${V}_{erri}$: The error voltage
${U}_{AVRi}$: The controller signal of the AVR loop
$Obj$: The proposed objective function/performance index
$ST$: Settling time of the response (s)
$RT$: Rise time of the response (s)
${U}_{s}$: The peak undershoot of the response (Hz)—(p.u.)
${O}_{s}$: The peak overshoot of the response (Hz)—(p.u.)
$t$: The simulation time (s)
